# Supplementary material for: Integration of academic and health education for the prevention of physical aggression and violence in young people: systematic review, narrative synthesis and intervention components analysis
Source: BMJ Open. 2018 Sep 21;8(9):e020793. doi: 10.1136/bmjopen-2017-020793 (PMC6157571; doi:10.1136/bmjopen-2017-020793)
Supplement: Supplementary file 2 [file bmjopen-2017-020793supp002.pdf]

How can interventions integrating health and academic education in schools help prevent substance misuse and violence, and reduce health inequalities among young people? Systematic review and evidence synthesis

Chris Bonell, James Thomas, Adam Fletcher, Rona Campbell, GJ Melendez-Torres, Claire Stansfield, Tara Tancred

Citation

Chris Bonell, James Thomas, Adam Fletcher, Rona Campbell, GJ Melendez-Torres, Claire Stansfield, Tara Tancred. How can interventions integrating health and academic education in schools help prevent substance misuse and violence, and reduce health inequalities among young people? Systematic review and evidence synthesis. PROSPERO 2015 CRD42015026464 Available from: http://www.crd.york.ac.uk/PROSPERO/display_record.php?ID=CRD42015026464

Review question

RQ1. What types of curriculum interventions integrating health and academic education in schools addressing substance use and violence have been evaluated?
RQ2. What theories of change inform these interventions and what do these suggest about their potential mechanisms and effects?
RQ3. What characteristics of interventions, deliverers, participants and school contexts facilitate or limit successful implementation and receipt of such interventions, and what are the implications of these for delivery in the UK?
RQ4. How effective are such interventions in reducing alcohol consumption, smoking, drug use and violence, and increasing attainment, when compared to usual treatment, no treatment, or other interventions, and does this vary according to students' socio-demographic characteristics?
RQ5. What characteristics of interventions, deliverers, school contexts and students appear to moderate or are necessary and sufficient for the effectiveness of such interventions?

Searches

Our search strategy will be informed by those used in previous systematic reviews focused on school interventions addressing alcohol, smoking, drug use and violence. The studies sought by this review are not likely to be reliably indexed in databases with controlled vocabularies. So we anticipate our searches involving a large number of free text terms. We will take the following three key concepts from the inclusion criteria to develop the search string: health education; integration with academic learning; and children and young people or schools. The combination of these concepts is sensitive enough to include all available studies regardless of study design. The three concepts will be linked by the Boolean operator "AND". Our searches will involve different free text and controlled vocabulary terms for each of these two concepts linked by the Boolean operator "OR". In our use of terms relating to health education, we will use a very broad array of terms to minimise the risk of publication bias. We will not restrict the searches by date, language or publication type. We will search the following databases from inception to present: ASSIA; Australian Educational Index; BiblioMap (Database of health promotion research); British Educational Index; Cochrane Central Register of Controlled Trials; Cochrane Database of Systematic Reviews; Database of Abstracts of Reviews of Effects; Database of Promoting Health Effectiveness Reviews; Econlit; ERIC; Health Technology Assessments; IBSS (International Bibliography of the Social Sciences); Medline; NHS Economic Evaluation Database; Proquest Dissertation Abstracts; PsycInfo; Social Policy and Practice including Child Data & Social Care Online; Social Science Citation Index/Web of Knowledge; ; and Trials Register of Promoting Health Interventions. We will carefully search reference lists from all studies that meet the inclusion criteria. We will hand-search journals that published included studies which we found only via reference checking and which are not indexed on databases we have searched (initially for the last 5 years and if these elicit >1 new included studies, for a further 5 years). We will search the following websites: the Campbell Library;

Digital Education Research Archive; OpenGrey (System for Information on Grey Literature in Europe); Database of Educational Research; International Clinical Trials Registry Platform; Schools and Students Health Education Unit Archive. We will contact subject experts to identify relevant ongoing or completed research. We will search all available clinical trials registers (e.g. clinicaltrials.gov) for relevant ongoing and unpublished trials.

Types of study to be included

In order to address RQ 1 and 3, we will include studies reporting on process evaluations. This would include studies reporting on planning, delivery, receipt or causal pathways using quantitative and/or qualitative data. These studies may report exclusively on process evaluations or report process alongside outcome data. In order to address RQ 1 and 4, we will include studies reporting on outcome evaluations, using randomized controlled trials allocating schools, classes or individuals. Controls will be students, classes or schools allocated randomly to a control group in which no or usual school health and academic education is delivered, or to a control group including another ‘active’ intervention. In order to address RQ2 we will draw on included process and outcome evaluations as defined above which include descriptions of intervention theories of change or logic models. In order to address RQ5, we will draw on syntheses of all of the above study types.

Condition or domain being studied

The proposed review focuses on substance use (alcohol consumption, smoking and drug use) and violence since these are important, inter-correlated outcomes which are addressed by interventions sharing common theories of change. Alcohol has been suggested to be the most harmful substance in the UK. Treating alcohol-related diseases costs the NHS in England an estimated £3.5 billion annually. The total annual societal costs of alcohol use in England are estimated at £21 billion. Alcohol related harms are strongly stratified by socioeconomic status (SES). Early initiation of alcohol use and excessive drinking are linked to later heavy drinking and alcohol-related harms and poor health. Alcohol use among young people is associated with truancy, exclusion, and poor attainment, as well as unsafe sexual behaviour, unintended pregnancies, youth offending, accidents/ injuries and violence. Preventing young people from taking up smoking is another key public health objective with 80,000 deaths due to smoking each year. In 2005-6, smoking cost the NHS £5.2 billion and wider costs amounted to £96 billion. Of smokers, 40% start in secondary school and early initiation is associated with heavier and more enduring smoking and greater mortality. Smoking among young people is a major source of health inequalities. Among UK 15-16 years olds 25% have used cannabis and 9% have used other illicit drugs. Early initiation and frequent use of ‘soft’ drugs may be a potential pathway to more problematic drug use in later life. Drugs such as cannabis and ecstasy are associated with increased risk of mental health problems, particularly among frequent users. Young people’s drug use is also associated with accidental injury, self-harm, suicide and other ‘problem’ behaviours. The proposed review’s other primary outcome is violence. The prevalence, harms and costs of violence among young people mean that addressing this is a public health priority. One UK study found that 10% of young people aged 11-12 reported carrying a weapon and 8% admitted attacking someone with intent to hurt them seriously. By age 15-16, 24% of students report that they have carried a weapon and 19% reported attacking someone with the intention to hurt them seriously. There are also links between aggression and anti-social behaviours in youth and violent crime in adulthood. As well as leading to further health inequalities, the economic costs to society of youth aggression, bullying and violence are high. For example, the total cost of crime attributable to conduct problems in childhood has been estimated at about £60 billion a year in England and Wales.

Participants/population

We will include studies conducted where a majority of participants are children and young people aged 4-18 years attending schools.

Intervention(s), exposure(s)

We will include school-based health curriculum interventions integrating health and academic education targeting young people age 4-18. Academic education is defined as: education in specific academic subjects; literacy; numeracy; or study skills. It does not include: social conduct in the classroom; relationships

with peers or staff; attitudes to education, school or teachers; or aspirations and life goals. Interventions may involve either incorporate health education into other, mainstream school subjects or aim for health education lessons to include academic education as well as teaching health knowledge and skills. Interventions may be delivered by teachers or other school staff such as teaching assistants, but may also be delivered by external providers, for example from the health, voluntary or youth service sectors. Our definition excludes interventions which: are delivered in mainstream subject lessons but do not aim to integrate health and academic education; train teachers in classroom management without student curriculum components; or are delivered exclusively outside of classrooms.

Comparator(s)/control

In order to address RQ 1 and 4, we will include studies reporting on outcome evaluations, using randomized controlled trials allocating schools, classes or individuals.
Controls will be students, classes or schools allocated randomly to a control group in which no or usual school health and academic education is delivered, or to a control group including another ‘active’ intervention.

Context

Schools serving students age 4-18 years.

Primary outcome(s)

We will include studies addressing one or more of the following primary review outcomes: smoking; alcohol use; legal or illegal drug use; and violence.

Timing and effect measures
We will include studies addressing one or more of the following primary review outcome measures: smoking (e.g. salivary cotinine, carbon monoxide levels, self-reported use of cigarettes); alcohol use (e.g. self-reported alcohol consumption via questionnaires or diaries); legal or illegal drug use (e.g. self-reported drug use); and violence (self-reported violence perpetration - for example, carried weapon, got into a fight - and victimisation). Informed by existing systematic reviews focused on substance use and violence among young people, outcome measures may draw on dichotomous or continuous variables, and self-report or observational data. They may use measures of frequency (monthly, weekly or daily), the number of episodes of use or an index constructed from multiple measures. Alcohol measures may examine alcohol consumption or problem drinking. Drug outcomes may examine drugs in general or specific illicit drugs, including drug convictions. Measures of violent and aggressive behaviour may examine the perpetration or victimization of physical violence including convictions for violent crime.
We will regard follow-up times of less than three months, three months to one year and more than one year post-intervention as different outcomes.

Secondary outcome(s)

Though not an inclusion criterion, we will assess academic attainment as a secondary outcome.

Timing and effect measures
Academic attainment might be measured as e.g. student standardised academic test scores, IQ tests or other validated scales; school academic performance.

Data extraction (selection and coding)

Selection of studies
Search results will be downloaded into EPPI-Reviewer 4. An inclusion criteria worksheet with guidance notes will be prepared and piloted by two reviewers screening the same 50 references. Where the two reviewers disagree, they will meet to discuss this and if possible reach a consensus. If the reviewers cannot reach consensus regarding inclusion of a specific article, judgement for selection will be referred to a third reviewer. If necessary, we will organise translation of papers published in languages in which we are not proficient. After piloting and any refinements, each reference will be screened on the basis of title and abstract for potential inclusion by one reviewer, using text-mining to prioritise screening the most relevant studies first. Full reports will be obtained for those references judged as meeting our inclusion criteria or where there is

insufficient information from the title and abstract to judge inclusion. A second round of screening will then occur focused on full study reports to determine which studies are included in the review. We will maintain a record of the selection process for all screened material.
Data extraction and management
Two reviewers will independently extract data from all studies meeting the inclusion criteria, using a piloted data extraction form with guidance developed for this review. Where the two authors disagree, they will meet to discuss this and if possible reach a consensus. If the reviewers cannot reach consensus regarding the particulars of data extraction for a specific study, judgement will be referred to a third reviewer.
Included studies will be described using the EPPI-Centre classification system for health promotion and public health research, supplemented by additional codes developed for this review. For all studies where relevant, we will extract information pertaining to: basic study details (individual and organizational participant characteristics, study location, timing and duration, research questions or hypotheses); study design and methods (design, allocation, blinding, sample size, control of confounding, accounting for data clustering, data collection, attrition, analysis); intervention characteristics (timing and duration, programme development, theoretical framework/logic model, content and activities, providers and details of any intervention offered to the control group); process evaluation of the intervention (feasibility, fidelity/quality, intensity, coverage/accessibility, acceptability, mechanism and context using an adapted version of an existing tool); outcome measures at follow-up(s) (reliability of measures, effect size both overall and where available by age, sex, socio-economic status and ethnic sub-group). The two reviewers will independently enter data from the data extraction forms into EPPI-Reviewer 4. If included studies are reported in languages that cannot be translated by the review team, a review author will complete the data extraction form in conjunction with a translator.
Published reports may be incomplete in a wide range of ways. For example: they may not report sufficient detail about their participants for our equity analysis; they may not present information on all the outcomes that were measured (possibly resulting in outcome reporting bias); they may not provide sufficient information about the intervention for accurate characterisation; and they may not report the necessary statistical information for the calculation of effect sizes. In all cases where there is a danger of missing data affecting our analysis, we will contact authors of papers wherever possible to request additional information. If authors are not traceable or sought information is unavailable from the authors within two months of contacting them, we will record that the study information is missing on the data extraction form, and this will be captured in our risk of bias assessment of the study.

Risk of bias (quality) assessment

We will assess the quality of theories of change using a modified version of the criteria developed in our ongoing NIHR-funded systematic review of positive youth development interventions, which for example assess the clarity with which constructs are defined and inter-related. We will assess the quality of the qualitative and quantitative elements of process evaluations using standard Critical Appraisal Skills Program and EPPI-Centre tools. These address the rigour of: sampling; data collection; data analysis; the extent to which the study findings are grounded in the data; whether the study privileges the perspectives of participants; the breadth of findings; and depth of findings. These are then used to assign studies to two categories of ‘weight of evidence’. First, reviewers will assign a weight (low, medium or high) to rate the reliability or trustworthiness of the findings (the extent to which the methods employed were rigorous/could minimise bias and error in the findings). Second, reviewers will assign an additional weight (low, medium, high) to rate the usefulness of the findings for shedding light on factors relating to the research questions. Guidance will be given to reviewers to help them reach an assessment on each criterion and the final weight of evidence. The two reviewers will then meet to compare their assessments, resolving any differences through discussion and, where necessary, by calling on a third reviewer. For outcome evaluations, we will assess risk of bias within each included study using the tool outlined in the Cochrane Handbook for Systematic Reviews of Interventions. For each study, two reviewers will independently judge the likelihood of bias in seven domains: sequence generation; allocation concealment; blinding (of participants, personnel, or outcome assessors); incomplete outcome data; selective outcome reporting; and other sources of bias (e.g. recruitment bias in cluster-randomised studies); and intensity/type of comparator. Each study will subsequently be identified as ‘high risk’, ‘low risk’ or ‘unclear risk’ within each domain. In cases of

disagreement, the reviewers will meet to seek consensus but where they cannot, we will refer judgement to a third reviewer. We will assess reporting bias according to Sterne's guidance. We will reduce the effect of reporting bias by focusing synthesis on studies rather than publications, avoiding duplicated data. Following the Cho statement on redundant publications, we will attempt to detect duplicate studies and, if multiple articles report on the same study, we will extract data only once. We will prevent location bias by searching across multiple databases. We will prevent language bias by not excluding any article based on language.

Strategy for data synthesis

RQ1 and 2: Thematic synthesis of intervention descriptions and process data:
Using thematic synthesis methods we will undertake a number of syntheses. Intervention descriptions (RQ1) and theories of change (RQ2) will first be analysed to develop a taxonomy of interventions integrating health and academic education. Syntheses of theories of change (RQ2) and process evaluations (RQ3) will be used to understand potential mechanisms of action. Syntheses of process evaluations (RQ3) will be used to understand: characteristics of interventions, participants and context acting as potential barriers and facilitators of implementation and receipt (RQ2); and an assessment of potential applicability to the UK. These syntheses will not be restricted to studies judged to be of high quality. Instead conclusions drawing on poorer quality evidence will be given less interpretive weight. First, the reviewers will prepare detailed evidence tables to describe: the methodological quality of each study; details of the intervention examined; study site/population; and full findings. Second, the two reviewers will undertake pilot analysis of two studies. The reviewers will read and re-read data contained within the evidence tables relating to the two high-quality studies, applying line-by-line codes to capture the content of the data. They will draft memos explaining these codes. Coding will begin with in-vivo codes which closely reflect the words used in findings sections. The reviewers will then group and organise codes, applying axial codes reflecting higher-order themes. The two reviewers will meet to compare and contrast their coding of these first two high-quality studies, developing an overall set of codes. Third, the two reviewers will go on to code the remaining studies drawing in the agreed set of codes but developing new in-vivo and axial codes as these arise from the analytical process, and again writing memos to explain these codes. At the end of this process, the two reviewers will meet to compare their sets of codes and memos. They will identify commonalities, differences of emphasis and contradictions with the aim of developing an overall analysis which draws on the strengths of the two sets of codes and which resolves any contradictions or inconsistencies, drawing on a third reviewer if necessary to achieve this. Through this process will be developed an explanatory framework to understand factors affecting implementation. Results will be presented to PPI stakeholders who will determine which interventions they think are applicable to the UK.
RQ4: Synthesis of outcome data:
We will first produce a narrative account of the effectiveness of these types of interventions. This narrative synthesis will be ordered by outcome then within this by age group, intervention type and follow-up time. Outcomes will be categorised into violence, smoking tobacco, drinking alcohol, using other drugs and academic attainment. Age will be categorised by the key-stage age-ranges used in the English educational system. Categorisation by intervention type will be informed by our prior thematic synthesis of intervention descriptions and theories of change through which we will have produced a taxonomy of interventions. This taxonomy may refer to: whether interventions incorporate health education into other, mainstream school subjects or aim for health education lessons to include teaching of academic as well as health knowledge and skills; lesson frequency; style of delivery; or other aspects of interventions which appear to be critical from our preliminary synthesis. We will describe study results in the 'characteristics of included studies' table, or enter the data into additional tables. We will then produce forest plots for each of our review outcomes, with separate plots for different outcomes and follow-up times, age groups and intervention types. Plots will include point estimates and standard errors for each study, such as risk ratios for dichotomous outcomes or standardised mean differences for continuous outcomes. Once we know the number of studies and the extent of heterogeneity among the studies (as determined both by a Cochran's Q test and inspection of the I2), we will make a decision whether to calculate pooled effect sizes. The results of statistical tests will be evaluated in accordance with the Cochrane handbook. If an indication of substantial heterogeneity is determined (e.g. study-level I^2 value greater than 50%) that cannot be explained through meta-regressions, then we will not produce a pooled estimate and will present only the narrative summary.

When studies are found to be statistically heterogeneous, we will use a random-effects model; otherwise we will use a fixed-effects model. When using the random-effects model, we will conduct a sensitivity check by using the fixed-effect model to reveal differences in results. If we do produce pooled estimates, we will consider using a multilevel meta-analysis model to synthesise effect sizes. This is because outcome evaluations are likely to include multiple measures of conceptually related outcomes and multi-level meta-analysis improves on previous strategies for dealing with multiple relevant effect sizes per study, such as meta-analysing within studies or choosing one effect size, by including all relevant effect sizes but adjusting for inter-dependencies within studies. Unlike multivariate meta-analysis, it does not require the variance-covariance matrix of included effect sizes to be known. We will estimate separate models for substance use, violence and educational attainment outcomes. We will estimate separate models for substance use, violence and educational attainment outcomes, and for different age-ranges. We will examine substance use outcomes together in one analysis, as well as separated into smoking, alcohol, illicit drug use and any 'omnibus' measures of substance use. We will regard follow-up times of less than three months, three months to one year and more than one year post-intervention as different outcomes. We will run these models for interventions overall and where sufficient studies are found we will run separate models for different intervention categories and comparators. This categorisation will be informed by the taxonomy derived from our prior synthesis of intervention descriptions and theories of change. Where meta-analyses are performed, we will include pooled effect sizes in forest plots, with the individual study point estimates weighted by a function of their precision.
Prior to synthesis, we will check for correct analysis (where appropriate) by cluster and report values of: intra-cluster correlation coefficients (ICC), cluster size, data for all participants or effect estimates and standard errors. Where proper account has not been taken of data clustering, we will correct for this by inflating the standard error by the square root of the design effect. Where ICCs are not reported, we will contact authors to request this information or impute one, based on values reported in other studies. Where imputation is necessary, we will undertake sensitivity analyses to assess the impact of a range of possible values. In other instances of missing data (such as missing population information), it may not be possible to include a study in a particular analysis if, for example, it is impossible to classify the population using our equity tool. We will use the GRADE approach as described in the Cochrane Handbook for Systematic Reviews of Interventions to present the quality of evidence and 'Summary of findings' tables. The downgrading of the quality of a body of evidence for a specific outcome will be based on five factors: limitations of study; indirectness of evidence; inconsistency of results; precision of results; and publication bias. The GRADE approach specifies four levels of quality (high, moderate, low and very low). If sufficient studies are found, we will draw funnel plots to assess the presence of possible publication bias (trial effect versus standard error). While funnel plot asymmetry may indicate publication bias, this can be misleading with a small number of studies. We will discuss possible explanations for any asymmetry in the review in light of our number of included studies. We will undertake a sensitivity analysis to explore whether the findings of the review are robust in light of the decisions made during the review process. We will also assess the impact of risk of bias in the included studies via restricting analyses to studies deemed to be at low risk of selection bias, performance bias and attrition bias. Where data allow, we will undertake additional exploratory meta-analyses to determine intervention effects on theorised intermediate outcomes (such as knowledge, skills, social norms) to examine the plausibility that these might mediate or otherwise precede behavioural effects. Such analyses will be informed by the synthesis of theories of change and process evaluation findings to avoid data-dredging.

Analysis of subgroups or subsets

If we consider that we have unexplained statistical heterogeneity in any of our study groupings, we will investigate this further using subgroup and sensitivity analyses. We will analyse the effectiveness of the subset of interventions identified by stakeholders as relevant to the UK context. Where possible we will examine intervention effects by participant sub-groups (for example in terms of age, socioeconomic status, sex and ethnicity) and contexts (for example in terms of school-level deprivation) in order to examine potential impacts on health inequalities. This will draw on existing methods involving an 'equity lens' employing meta-analyses of subgroup effects from included studies and/or meta-regression drawing on studies with different participant or site characteristics to assess whether these moderate effects.
RQ5: Meta-regression and qualitative comparative analysis:

If at least ten studies are found, we will employ meta-regression using Stata to investigate what factors moderate intervention effects in order to examine what characteristics of intervention, deliverers, contexts and students moderate effectiveness (RQ5). It may not be feasible to apply this method if we judge there are too many confounders or insufficient data, or if meta-regression is unable to account for interdependencies in complex interventions. Hence, we will complement meta-regression with qualitative comparative analysis, adapted for use in research synthesis to assess necessary and sufficient conditions for intervention effectiveness. As with our current review of positive youth development, the use of initial hypotheses derived from work addressing RQ 2 and 3 will protect us from 'dredging' the data for spurious statistically significant results. The required steps of 'qualitatively anchoring' outcomes in qualitative comparative analysis will ensure that changes in outcomes are meaningful and not simply statistical artefacts with little relevance for decision-making. We should stress that meta-regression and qualitative comparative analysis will be exploratory, hypothesis-building analyses since these will draw on observational rather than experimental comparisons.

Contact details for further information

Chris Bonell
c.bonell@ioe.ac.uk

Organisational affiliation of the review

University College London Institute of Education
http://www.ioe.ac.uk/index.html

Review team members and their organisational affiliations

Professor Chris Bonell. UCL Institute of Education
Professor James Thomas. UCL Institute of Education
Dr Adam Fletcher. Cardiff University
Professor Rona Campbell. University of Bristol
Dr GJ Melendez-Torres. University of Warwick
Ms Claire Stansfield. UCL Institute of Education
Dr Tara Tancred. UCL Institute of Education

Collaborators

Dr Rob Anderson. University of Exeter

Anticipated or actual start date

01 January 2016

Anticipated completion date

31 August 2017

Funding sources/sponsors

National Institute for Health Research Public Health Research Programme Grant Number 14/52/15

Conflicts of interest

None known

Language

English

Country

England, Wales

Published protocol

http://www.crd.york.ac.uk/PROSPEROFILES/26464_PROTOCOL_20160011.pdf

Stage of review

Review_Ongoing

Subject index terms status

Subject indexing assigned by CRD

Subject index terms

Drug Users; Health Education; Humans; Prescription Drug Misuse; Schools; Socioeconomic Factors; Substance-Related Disorders; Violence

Date of registration in PROSPERO

22 September 2015

Date of publication of this version

11 January 2016

Revision note for this version

Modifications made 11/1/16Searches amended slightly - reflecting advice of information scientist Claire Stansfield.Inclusion criteria modified slightly - interventions integrating health and academic biology education no longer excluded

Details of any existing review of the same topic by the same authors

Stage of review at time of this submission

The review has not started

Stage	Started	Completed
Preliminary searches	No	No
Piloting of the study selection process	No	No
Formal screening of search results against eligibility criteria	No	No
Data extraction	No	No
Risk of bias (quality) assessment	No	No
Data analysis	No	No

Revision note

Modifications made 11/1/16Searches amended slightly - reflecting advice of information scientist Claire Stansfield.Inclusion criteria modified slightly - interventions integrating health and academic biology education no longer excluded

Versions

22 September 2015
20 November 2015
11 January 2016
