# Supplementary material for: Integration of academic and health education for the prevention of physical aggression and violence in young people: systematic review, narrative synthesis and intervention components analysis
Source: BMJ Open. 2018 Sep 21;8(9):e020793. doi: 10.1136/bmjopen-2017-020793 (PMC6157571; doi:10.1136/bmjopen-2017-020793)
Supplement: Supplementary file 1 [file bmjopen-2017-020793supp001.pdf]

Additional search and synthesis methods

We searched 19 electronic databases. The original list of databases was amended after consultation with our information scientist, as informed by initial searches.

- ASSIA via Proquest
- Australian Educational Index via Proquest
- BiblioMap (Database of health promotion research) via EPPI-Centre
- British Educational Index via EBSCOhost
- Cochrane Central Register of Controlled Trials via the Cochrane Library
- Cochrane Database of Systematic Reviews via the Cochrane Library
- Database of Abstracts of Reviews of Effects via the Cochrane Library
- Database of Promoting Health Effectiveness Reviews (DoPHER) via EPPI-Centre
- Dissertation Abstracts (UK theses, all dates; global theses 2010-2015) via Proquest
- Econlit via EBSCO
- Educational Research Index Citations via EBSCO
- Health Technology Assessment Database via the Cochrane Library
- International Bibliography of the Social Sciences via Proquest
- MEDLINE via OVID
- NHS Economic Evaluation Database
- PsycINFO via OVIDa
- Social Policy and Practice Including Child Data & Social Care Online via OVID
- Social Science Citation Index via Web of Knowledge
- Trials Register of Promoting Health Interventions via EPPI-Centre

We also searched the following 32 websites:

- Cambridge Journals
- Centers for Disease Control and Prevention: Smoking & Tobacco Use
- Child and Adolescent Research Unit
- Childhoods Today
- Children in Scotland
- Children in Wales
- Community Research and Development Information Service
- Database of Educational Research (EPPI-Centre)
- Drug and Alcohol Findings Effectiveness Bank
- Google
- Google Scholar
- Government of Wales
- Government of Scotland
- Joseph Rowntree Foundation
- National Criminal Justice Reference Service
- National Society of the Prevention of Cruelty to Children
- National Youth Agency
- Northern Ireland Executive
- OpenGrey
- Personal Social Services Research Unit
- Project Cork
- UCL-IOE Digital Education Resource Archive
- UK Clinical Research Net Study Portfolio
- University of Illinois at Urbana Champaign
- US Centre for Substance Abuse Prevention
- Social Issues Research Centre
- The Campbell Library
- The Children's Society
- The Open Library
- The Schools and Students' Health Education Unit Archive
- WHO International Clinical Trials Registry Platform
- Young Minds: Child & Adolescent Mental Health

PsycINFO search string

1. ((substance? or drug? or drinking or alcohol* or solvent?) adj1 ("use" or abus* or misuse*)).ti,ab.
2. ((substance? or drug? or drinking or alcohol* or solvent?) adj1 (usage or intake or using or taking or behavio* or user?)).ti,ab.
3. (drinking adj1 (alcohol* or behavio*)).ti,ab.
4. Alcohol.ti,ab.
5. (smoke or smoking or tobacco or cigarette? or smoker? or cannabis or marijuana).ti,ab.
6. (aggression or aggressive or bully* or delinquen* or "conduct problem*" or "conduct disorder?" or "antisocial" or "anti social" or violence or violent or (volatile adj behavio*) or victimi* or hostile or hostility or perpetrat*).ti,ab.
7. (Externalising or externalizing).ti,ab.
8. emotion*.ti,ab.
9. PSHE.ti,ab.
10. ("Health literacy" or "health education" or "health promotion" or "preventive health" or "primary prevention" or "health information" or "promoting health" or "health promoting" or "health promotion" or "health maintenance").ti,ab.
11. "Public health".ti,ab.
12. ("wellbeing" or "well being").ti,ab.
13. "mental health".ti,ab.
14. 1 or 2 or 3 or 4 or 5 or 6 or 7 or 8 or 9 or 10 or 11 or 12 or 13
15. ((curric* or lesson? or classes or classroom? or subject? or intervention? or program* or education or initiative? or learn or learning or teach or teaching or outcome* or attainment or achievement or assessment or effect* or impact* or score? or scoring* or skill? or knowledge or competen* or performance) adj3 (Academic or academically or Scholastic or scholar* or Mainstream or "main stream")).ti,ab.
16. ((curric* or lesson? or classes or classroom? or subject? or learn or learning or teach or teaching or attainment or achievement or assessment or score? or scoring* or skill? or knowledge or competen* or performance) adj3 School?).ti,ab.
17. ((intervention? or program* or initiative? or effect* or impact* or education) adj1 School?).ti,ab.
18. (class adj1 (Academic or academically or Scholastic or scholar* or School? or Mainstream or "main stream")).ti,ab.
19. ((curric* or lesson? or classes or classroom? or subject? or education or learn or learning or teach or teaching or attainment or achievement or score? or scoring* or skill? or knowledge or competen*) adj3 (study or core or generic)).ti,ab.
20. (class adj1 (study or core or generic)).ti,ab.
21. ((curric* or lesson? or classes or classroom? or subject? or attainment or achievement or assessment or score? or scoring* or competenc* or performance) adj3 ((Education not ("patient education" or "continuing education")) or educational)).ti,ab.
22. (class adj1 ((Education not ("patient education" or "continuing education")) or educational)).ti,ab.
23. (outcome* adj1 (education or educational)).ti,ab.
24. ((curric* or lesson? or classroom? or classes or subject? or intervention? or program* or initiative? or education or teach* or outcome* or attainment or achievement or assessment or effect* or impact* or score? or scoring* or skill? or knowledge or competen* or performance) adj3 (learn or learning)).ti,ab.
25. (class adj1 (learn or learning)).ti,ab.
26. ((curric* or lesson? or classes or classroom or class or subject? or education or teach* or learning or teach or teaching or learn or attainment or achievement or assessment or score? or scoring* or skill? or knowledge or competen* or performance) adj3 (art or arts or math* or science? or humanities or chemistry or physics or language* or geography or (history not ("medical history" or "health history" or "familial history" or "family history")) or numeracy or (literacy not "health literacy") or grammar or grammer or reading or writing)).ti,ab.
27. (((curric* or lesson? or classroom or classes or subject? or skill?) adj3 literature) or "literature class").ti,ab.
28. ("Education reform" or "Instructional support" or "School reform" or "Classroom organi*" or (Commit* adj3 (school or education or learning)) or (Engag* adj3 (school or education or learning)) or "Character development" or "Whole school" or "School level" or "School wide" or schoolwide).ti,ab.
29. ((Comprehensive adj3 school) and (intervention? or program* or initiative? or outcome* or effect* or impact*)).ti,ab.
30. ((Integrat* or Combin* or Infuse or infused or infusion or sustainable) adj3 (curric* or lesson? or classes or classroom or syllabus or subject? or education or learn or learning or teach or teaching)).ti,ab.
31. (((Integrat* or Combin* or Infuse or infused or infusion or sustainable) adj3 (intervention* or program* or initiative*)) and school?).ti,ab.
32. ((school or education or core or generic or teaching or learning) adj3 syllabus).ti,ab.

33. 15 or 16 or 17 or 18 or 19 or 20 or 21 or 22 or 23 or 24 or 25 or 26 or 27 or 28 or 29 or 30 or 32
34. (child* or schoolchild* or youth* or "young people*" or "young person" or teen* or adolescen* or juvenile* or preadolescen* or boy? or girl?).ti,ab.
35. (curric* or lesson? or classes or classroom? or subject? or school? or syllabus or "junior high" or "senior high" or "junior education" or "elementary education" or "primary education").ti,ab.
36. 34 and 35
37. ("secondary school?" or "primary school?" or "comprehensive school?" or "school education" or "high school?" or "grammar school?" or "private school?" or "public school?" or "mainstream school*" or "compulsory education" or "statutory education" or "middle school?" or "junior school?" or "senior school?" or "primary education" or "secondary education" or "elementary school?" or "elementary education" or "mainstream education" or "compulsory school*" or "statutory school*" or "sixth form college?" or "post-16 education" or "junior high" or "senior high" or "reception class" or "post primary").ti,ab.
38. ((school? or junior? or elementary or senior? or primary or "sixth form" or grade) adj10 student?).ti,ab.
39. pupil?.ti,ab.
40. 36 or 37 or 38
41. (University or universities or freshmen or sophomore? or "higher education" or "tertiary education" or ((registrar* or workplace? or clinical or medical or nursing or nurse? or doctor? or continuing or adult? or patient?) adj1 (education or educating or profession* or student?)) or "professional education").ti.
42. 40 not 41
43. 14 and 33 and 42
44. "Elementary School Students"/ or "Intermediate School Students"/ or "Primary School Students"/ or "Middle School Students"/ or "High School Students"/ or "Junior High School Students"/ or "Kindergarten Students"/ or "High School Education"/ or "Middle School Education"/ or "Secondary Education"/ or "Junior High Schools"/ or "High Schools"/ or "Schools"/ or "Elementary Schools"/ or "Middle Schools"/
45. "Drug Abuse Prevention"/ or "Health Education"/ or "Drug Education"/ or "Health Promotion"/ or "Public Health"/ or "Health Promotion"/ or "Preventive Medicine"/ or Health behaviour/ or Harm reduction/ or Health literacy/ or exp Health screening/ or Primary Mental health prevention/ or Prevention/ or Public health/ or Lifestyle changes/ or Lifestyle/ or Health literacy/
46. "Tobacco Smoking"/ or "Smoking Cessation"/ or "Marijuana Usage"/ or "Drinking Behavior"/ or "Social Drinking"/ or "Binge Drinking"/ or "Underage Drinking"/ or "Alcohol Abuse"/ or "Alcohol Drinking Patterns"/ or "Alcohol Intoxication"/ or "Alcoholism"/ or "Heroin Addiction"/ or "Drug Addiction"/ or "Drug Dependency"/ or "Drug Usage"/ or "Inhalant Abuse"/ or "Drug Abuse"/ or "Glue Sniffing"/ or "Predelinquent Youth"/ or "Cyberbullying"/ or "School Violence"/ or "Teasing"/ or "Juvenile Delinquency"/ or "Physical Abuse"/ or "Verbal Abuse"/ or "Violence"/ or "Harassment"/ or "Antisocial Behavior"/ or "Bullying"/ or "Perpetrators"/ or "Threat"/ or "Victimization"/ or "Relational Aggression"/ or "Aggressive Behavior"/ or "Behavior Problems"/ or "Behavior Disorders"/ or "Conduct Disorder"/ or "Drug Education"/ or "Drug Abuse Prevention"/ or "Harm Reduction"/
47. emotions/ or emotional development/
48. emotional adjustment/ or emotional disturbances/ or emotional control/
49. mental health/ or primary mental health prevention/ or well being/
50. "Curriculum"/ or "Curriculum Based Assessment"/ or "Curriculum Development"/ or "School Learning"/ or "Classroom Environment"/ or "Academic Environment"/ or "Teacher Effectiveness"/ or "Teacher Effectiveness Evaluation"/ or "Educational Program Evaluation"/ or "Course Evaluation"/ or "learning environment"/
51. 14 or 45 or 46 or 47 or 48 or 49
52. 33 or 50
53. 42 or 44
54. 51 and 52 and 53
