# Supplementary material for: Integration of academic and health education for the prevention of physical aggression and violence in young people: systematic review, narrative synthesis and intervention components analysis
Source: BMJ Open. 2018 Sep 21;8(9):e020793. doi: 10.1136/bmjopen-2017-020793 (PMC6157571; doi:10.1136/bmjopen-2017-020793)
Supplement: Author's manuscript [file bmjopen-2017-020793.draft_revisions.pdf]

BMJ Open

BMJ Open is committed to open peer review. As part of this commitment we make the peer review history of every article we publish publicly available.

When an article is published we post the peer reviewers' comments and the authors' responses online. We also post the versions of the paper that were used during peer review. These are the versions that the peer review comments apply to.

The versions of the paper that follow are the versions that were submitted during the peer review process. They are not the versions of record or the final published versions. They should not be cited or distributed as the published version of this manuscript.

BMJ Open is an open access journal and the full, final, typeset and author-corrected version of record of the manuscript is available on our site with no access controls, subscription charges or pay-per-view fees (http://bmjopen.bmj.com).

If you have any questions on BMJ Open's open peer review process please email editorial.bmjopen@bmj.com

BMJ Open

Integration of academic and health education for the prevention of violence in young people: systematic review, narrative synthesis and intervention components analysis

Journal:	*BMJ Open*
Manuscript ID	bmjopen-2017-020793
Article Type:	Research
Date Submitted by the Author:	24-Nov-2017

Complete List of Authors:	Melendez-Torres, G.J.; Cardiff University, DECIPHer, School of Social Sciences<br>Tancred, Tara; London School of Hygiene and Tropical Medicine, Department of Social and Environmental Health Research<br>Fletcher, Adam; British Heart Foundation Cymru<br>Campbell, Rona; University of Bristol, DEICPHer, Bristol Medical School<br>Thomas, James; University College London Social Science Research Unit<br>Bonell, Christopher; London School of Hygiene and Tropical Medicine, Department of Social and Environmental Health Research

Keywords:	systematic review, violence, Community child health < PAEDIATRICS

SCHOLARONE™
Manuscripts

Integration of academic and health education for the prevention of violence in young people: systematic review, narrative synthesis and intervention components analysis

Dr G.J. Melendez-Torres [1]*

Dr Tara Tancred [2]

Prof Adam Fletcher [3]

Prof Rona Campbell [4]

Prof James Thomas [5]

Prof Chris Bonell [2]

[1] DECIPHer, School of Social Sciences, Cardiff University, Cardiff, UK

[2] Department of Social and Environmental Health Research, London School of Hygiene and Tropical Medicine, London, UK

[3] British Heart Foundation Cymru, Cardiff, UK

[4] DECIPHer, Bristol Medical School, University of Bristol, Bristol, UK

[5] EPPI-Centre, UCL Institute of Education, University College London, London, UK

*Corresponding author
G.J. Melendez-Torres
DECIPHer
School of Social Sciences
Cardiff University
1-3 Museum Place
Cardiff CF10 3BD
UNITED KINGDOM
melendez-torresg@cardiff.ac.uk
+44 (0) 29208 79106

Word count: 2,992

For peer review only

Abstract

Objectives. To systematically review evidence on the effectiveness of interventions including integration of academic and health education for reducing violence, and describe the content of these interventions.

Data sources. Between November and December 2015, we searched 19 databases and 32 websites and consulted key experts in the field.

Eligibility criteria. We included randomised trials of school-based interventions integrating academic and health education in students aged 4-18 and not targeted at health-related subpopulations (e.g. learning or developmental difficulties). We included evaluations reporting a measure of interpersonal violence or aggression.

Data extraction and analysis. Data were extracted independently in duplicate, interventions were analysed to understand similarities and differences, and outcomes were narratively synthesised by key stage (KS).

Results. We included 10 evaluations of eight interventions were reported in 14 papers. Interventions included either full or partial integration, incorporated a variety of domains beyond the classroom, and used literature, local development or linking of study skills and health promoting skills. Evidence was concentrated in KS2, with few evaluations in KS3 or KS4, and evaluations had few consistent effects; evaluations in KS3 and KS4 did not suggest effectiveness.

Discussion. Integration of academic and health education may be a promising approach, but more evidence is needed. Future research should consider the 'lifecourse' aspects of these interventions; that is, do they have a longitudinal effect? Evaluations did not shed light on the value of different approaches to integration.

For peer review only

Strengths and limitations of this study

- We used an exhaustive search including 19 databases and 32 websites.
- We used an innovative method to describe key components in this class of interventions.
- However, it was challenging to identify studies for inclusion.
- Meta-analysis was not possible because of the diversity of outcomes and raters.

For peer review only

INTRODUCTION

Violence among young people is a public-health priority due to its prevalence and harms to young people and wider society.[1 2] One UK study found that 10% of young people aged 11–12 reported carrying a weapon and 8% admitted attacking someone with intent to hurt them seriously.[3] By age 15–16, 24% of students reported they have carried a weapon and 19% reported attacking someone with the intention to hurt them seriously.[3] Early aggression and anti-social behaviour are strongly linked to adult violent behaviour.[4 5]

School-based health education can be effective in reducing violence.[6-8] However, school-based health education is increasingly marginal in many high-income countries, partly because of schools increasing focus on attainment-based performance metrics. In England specifically, health education is not a statutory subject,[9-11] and school inspectors have a limited focus on how schools promote student health.[12]

One way to avoid such marginalisation is to integrate health education into academic lessons. For example, health-related content can be reflected in academic lessons, by incorporating anti-violence messages within academic subjects, or by linking academic study skills with health promotion skills. This strategy may bring other benefits because: larger ‘doses’ may be delivered; students may be less resistant to health messages weaved into other subjects; and lessons in different subjects may reinforce each other.[13 14] Conversely, those teaching academic subjects may be uninterested or unqualified to teach health topics. Though theories of change in this class of interventions are diffuse, one important way in which they could be effective is by promoting developmental cascades involving the interplay of cognitive and non-cognitive skills.[15 16] Interventions integrating academic and health education could address violence by developing: social and emotional skills such as self-awareness, self-regulation, motivation, empathy and communication;[17] healthier social support or norms among students[15 18 19]; knowledge of the costs [20] and consequences [21] of

For peer review only

substance use; media literacy skills to critique harmful media messages; and modifying students' social norms about antisocial behaviours.[13 20 22-24]

Despite policy interest in these interventions, they have not previously been the subject of a specific systematic review. Our focus on violence is informed by preliminary consultation, scoping work and logic model development suggesting that violence is an outcome especially amenable to these interventions. In the present review, we examined the characteristics of interventions that integrate academic and health education to prevent violence, and synthesised evidence for their effectiveness.

METHODS

This review was part of a larger evidence synthesis project on theories of change, process evaluations and outcome evaluations of integration of academic and health education for substance use and violence. We registered the protocol for this review on PROSPERO (CRD42015026464, https://www.crd.york.ac.uk/prospero/).

Inclusion and exclusion

Studies were included regardless of publication date or language. We included randomised controlled trials of interventions integrating academic and health education, the former defined as specific academic subjects or general study skills. We included school-based interventions that incorporated health education into academic lessons and interventions that included health education lessons with academic components.

For this review, we focus on violence outcomes, defined as the perpetration or victimisation of physical violence including convictions for violent crime. We included outcomes that were a composite of physical and non-physical (e.g. emotional) interpersonal violence, but excluded composite measures that included items not focused on interpersonal violence, such as damage to property.

For peer review only

Interventions focusing on targeted health-related sub-populations (e.g. children with cognitive disabilities) were excluded as we were interested in universal interventions. We excluded interventions that trained teachers in classroom management.

Search strategy

We searched 19 databases and 32 websites, and contacted subject experts (see Online File 1 for full details).

Study selection

Pairs of researchers double-screened titles and abstracts in sets of 50 references until 90% agreement was reached. Subsequently, single reviewers screened each reference. We located the full texts of remaining references and undertook similar pairwise calibration followed by single screening.

Using an existing tool[25] we extracted data independently in duplicate from included studies and assessed trials for risk of bias using a modified version of the Cochrane assessment tool.[26]

We undertook an intervention components analysis.[27] This was undertaken inductively by one researcher and audited by two other researchers, and used intervention descriptions to draw out similarities and differences in intervention design using an iterative method. Finally, we synthesised outcomes narratively due to the heterogeneity in included outcome measurement. We categorised the timing of intervention effect by period of schooling, defined in terms of English schools' key-stage (KS) system. KS1 includes school years 1–2 (age 5–7 years); KS2 includes years 3–6 (age 7–11 years); KS3 includes years 7–9 (age 11–14 years); KS4 includes years 10–11 (age 14–16 years); and KS5 includes years 12–13 (age 16–18 years).

We could not formally assess publication bias because heterogeneity in outcome measurement precluded meta-analysis.

For peer review only

RESULTS

We found and screened 76,979 references, of which we retained 702 for full-text screening and were able to assess 690. Of 62 relevant reports included in the overall project, 10 evaluations of eight interventions were reported in 14 papers that considered violence and are reported in this review (Figure 1).

Included studies and their quality

All trials randomised schools except the Bullying Literature Project, which randomised classrooms (Table 1). All evaluations were conducted in the USA, except for Gatehouse,[28] which was an Australian study. All control arms consisted of education-as-usual or waitlist controls.

Interventions were diverse and are summarised below in the intervention components analysis. Only two interventions (Bullying Literature Project,[29] Youth Matters[30]) were wholly delivered by external staff. Several (Gatehouse,[28] Positive Action,[31] Steps to Respect[32]) linked classroom-based delivery to school-level work to support and reinforce implementation. PATHS[33] and 4Rs[19] also emphasised teachers' professional development.

Evaluation quality varied (Table 2). Appraisal was hampered by poor reporting of some aspects of trial methods. Only three studies reported evidence of low risk of bias for random generation of allocation sequence; the remainder were unclear. No evaluations reported information on concealed allocation. In LIFT,[34] outcome assessors were blinded, resulting in low risk of bias in this domain, but all other interventions were of unclear risk of bias. All interventions included reasonably complete outcome data, and in only one evaluation did unit of analysis issues pose a risk of bias. In some studies such as Steps to Respect, follow-up was shorter than intervention length.

For peer review only

Intervention components analysis

This identified four themes describing included interventions: approach to integration, position of integration, degree of integration and point of integration. Included interventions are described in Table 1, and the components analysis is summarised in Table 3.

Approach to integration

Interventions approached the rationale for and strategy of integration in different and overlapping ways. Several (4Rs, Bullying Literature Project, Steps to Respect, Youth Matters) focused on *literature* as a focus for integration, using children's books as a prompt for social-emotional learning. Another approach to integration emphasised *local development*, where teachers were encouraged to decide the most appropriate way to integrate activities into daily instruction (PATHS, Positive Action). A third approach was *linking to developmental concerns*, emphasising not so much the comprehensive integration of academic and health education but rather the interrelationships between academic success and broader development, health and wellbeing (Positive Action, Gatehouse). These interventions viewed academic education through a 'health' lens, in addition to viewing health education through an 'academic' lens.

Domains of integration

Some interventions (4Rs, Bullying Literature Project and Youth Matters) were exclusively *classroom-focused* while others (Gatehouse, Steps to Respect) used *classroom and whole-school* strategies to reinforce and extend learning. For example, Gatehouse involved school implementation-support teams, while Steps to Respect involved revision of anti-bullying policies. Other interventions, (PATHS, Positive Action) used *classroom, whole school environment and external domain* (parent information) strategies consistent with the health promoting schools approach promulgated particularly by the WHO which in the US is known as the Comprehensive School Health Program (CSHP) model.[35]

For peer review only

Degree of integration

In some interventions, health education was fully integrated (woven seamlessly) into everyday academic lessons (Gatehouse, 4Rs, Youth Matters), while in partially integrated interventions, health education involved distinct lessons, albeit also covering academic learning (Positive Action).

Timing of integration

Most interventions were multi-year, though two involved only one school year (LIFT, Bullying Literature Project).

Intervention effects

Perpetration measures included bullying (physical, or physical/verbal), aggression against peers and others, and violent behaviours including injuring others (Table 4). Measures involved different raters, including students, teachers and observers. Victimisation measures ranged from physical violence specifically to interpersonal aggression more generally. Heterogeneity of definition, measurement and form of effect sizes precluded meta-analysis.

Violence perpetration: KS2

Across the nine evaluations reporting outcomes in this KS, effects were inconsistent, including within studies by rater.

In LIFT,[34] effects at the end of the first intervention year on observed physical aggression in the playground were similar for students with different levels of baseline aggression(d=-0.14 at mean, 1 SD and 2 SD above the pre-intervention mean); these findings being described as 'statistically significant'. However, after the first intervention year of 4Rs,[19] there were no effects on teacher-reported aggression (b=0.02, SE=0.05, based on a 1-4 scale). After the second intervention year,[15] there were effects on teacher-reported student aggression (d=-0.21, p<0.05). The Bullying Literature Project also reported no effects on physical aggression rated by teachers for individual students (IG: M=1.12, SD=0.47, n=95 vs.

For peer review only

CG: 1.19, SD=0.47, n=55; *p*=0.67) or student self-reports (1.20, 0.44, n=90 vs. 1.14, 0.36, n=42; *p*=0.84) at one week post-intervention.[29]

Findings for Steps to Respect differed by type of rater. At the end of the first intervention year, the first evaluation of Steps to Respect[32] reported evidence of decreased bullying based on playground observation ($F(91.3)=5.02$, $p<0.01$) but not direct aggression based on student report ($F(68.7)=2.05$, $p>0.05$). The second evaluation of Steps to Respect[36] revealed a similar pattern. While teacher reports of physical bullying perpetration were less in intervention schools than in control schools at the end of the first intervention year (OR=0.61, $t(29)=-3.12$, $p<0.01$), student reports suggested no difference between schools on bullying perpetration ($t(29)=-1.06$). Moreover, in PATHS,[33] small positive effects of the intervention on student-reported aggression at the end of the first intervention year ($d=-0.048$, 95% CI [-0.189, 0.092]) and at the start (-0.064, [-0.205, 0.076]) and end (-0.048, [-0.188, 0.093]) of the second intervention year gave way to a small deleterious intervention effect at the end of the third year (0.082, [-0.060, 0.224]). Opposite effects were found on teacher-reported aggression, with initially small, negative intervention effects at the end of the first (0.036, [-0.105, 0.178]) and start of the second intervention year (0.035, [-0.107, 0.178]) but progressively greater effects at the end of the second (-0.005, [-0.146, 0.136]) and the third (-0.199, [-0.338, -0.060]) intervention years.

In contrast, two evaluations showed consistently positive results across different measures. In Positive Action Chicago,[31] students reported lower counts of bullying behaviours (IRR=0.59, 95% CI [0.37, 0.92]) and of serious violence-related behaviours, including cutting or stabbing someone on purpose (0.63, [0.45, 0.88]). Findings from Positive Action Hawaii[37] were similar for student-reported violent behaviours (IRR=0.42, 90% CI [0.24, 0.73]) and teacher-reported violent behaviours (0.54, [0.30, 0.77]). For students in the fourth or fifth intervention year, intervention recipients were less likely to report cutting or

For peer review only

stabbing someone (OR=0.29, 90% CI [0.16, 0.52]) or shooting someone (0.24, [0.14, 0.40]). Teachers were less likely to report that students hurt others (0.61, [0.38, 0.97]) or got into lots of fights (0.63, [0.47, 0.84]).

However, in Youth Matters,[30] students in intervention schools were not less likely to report bullying perpetration (OR=0.85, 95% CI [0.29, 1.47], *p*=0.585) after the second intervention year. Evaluators explored use of latent class analyses to classify intervention recipients as victims, bullies or bully-victims. Proportions of intervention and control recipients classified as bullies or bully-victims were not significantly different by study arm at the end of the first (IG: 21%, n=356 vs CG: 22%, n=392) or second (19%, n=244 vs 23%, n=293) intervention years.[38]

Violence perpetration: KS3

The two evaluations examining violence perpetration outcomes in KS3 had dissimilar results. At the end of the sixth intervention year of Positive Action Chicago,[39] students receiving the intervention reported lower counts of violence-related behaviours than no-treatment controls (IRR=0.38, 95% CI [0.18, 0.81]; equivalent to *d*=-0.54). Students also reported fewer bullying behaviours (*d*=-0.39), and parents reported that their children engaged in fewer bullying behaviours (*d*=-0.31). Significance values for these estimates were not presented, but both were supported by significant condition-by-time interactions in multilevel models, indicating that the intervention group showed an improved trajectory over time as compared to the control group. In contrast, after the third year from baseline in Youth Matters,[38] proportions of students were not different in the collective bully and bully-victim groups (both groups 16%; IG n=283, CG n=289).

Violence victimisation: KS2

While the five evaluations reporting outcomes in this KS were similar in follow-up period, they did not point to a clear effect. Students receiving the Bullying Literature Project

For peer review only

were not different from their peers in physical victimisation by teacher report on individual students (IG: M=1.04, SD=0.23, n=95 vs. CG: 1.04, SD=0.21, n=55; *p*=0.39) or student self-report (1.35, 0.54, n=90 vs. 1.43, 0.66, n=42; *p*=0.57) one week post-intervention.[29] PATHS measured student-reported victimisation using standardised mean differences, and found small, non-significant increases relative to the control arm at: the end of the first intervention year (*d*=0.044, 95% CI [-0.098, 0.185]); the start (0.074, [-0.067, 0.216]) and end (0.092, [-0.050, 0.234]) of the second year; and the end of the third year (0.089, [-0.053, 0.231]) of intervention implementation.[33] Steps to Respect, evaluated in two different trials, also found no differences in student-reported bullying victimisation at the end of the first intervention year in the first (IG: M=0.80, SD=1.51 vs CG: M=0.86, SD=1.44; *F*<1)[32] or second trial (2.11, 1.03 vs. 2.18, 1.06; *t*(29)=-1.15).[36] The first trial included playground observation at the end of the first intervention year, which was suggestive of lower levels in bullying victimisation, though these differences were marginally non-significant (0.9, 0.82 vs. 1.01, 0.83; *F*(72.4)=3.74, *p*<0.10).[32] Finally, Youth Matters examined bullying victimisation through continuous and dichotomous measures. At the end of the second intervention year, the difference in log-transformed continuous scores suggested a decrease (difference=-0.171, SE=0.083, *p*=0.049), as did the difference in dichotomous scores (OR=0.61, *p*=0.098).[30] However, the latent class analysis did not suggest a difference between groups at this point.[38]

Violence victimisation: KS3 and KS4

Intervention evaluations reporting violence victimisation outcomes in KS3 (Youth Matters [38 40] and Gatehouse[28]) and KS4 (Gatehouse[28]) suggested no evidence of effectiveness. In Youth Matters, differences in the log-transformed scores for bullying victimisation suggested a decrease in victimisation in intervention recipients as compared to controls, but this difference was not significant (difference=-0.123, SE=0.068, *p*=0.08).[40] However, at the end of the third intervention year, fewer students in the intervention than control group were

For peer review only

members of the victim or bully-victim classes (36%, n=283 vs 45%, n=289).[38] Based on our own chi-square test, this difference was significant (*p*=0.029). Gatehouse,[28] which was implemented from year 9, found no evidence of a change in bullying victimisation at the end of the first (OR=1.03, 95% CI [0.86, 1.26]), second (1.03, [0.78, 1.34]) or third (0.88, [0.68, 1.13]) intervention years, which corresponded to the first two years of KS4.

DISCUSSION

While the integration of academic and health education remains a promising model for the delivery of school-based health education, randomised evaluations were variable in quality and did not consistently report evidence of effectiveness in reducing violence victimisation or perpetration. Evidence was concentrated in KS2, with few evaluations in KS3 or KS4.

Though a formal moderator analysis was not possible, certain intervention models appear more effective than others. Specifically, evaluations of Positive Action in both Chicago[39] and Hawaii[37] showed consistently positive results across diverse measures. This may reflect the involvement of the intervention developer, a factor often associated with improved intervention fidelity (although Positive Action was not unique in this respect among interventions included in our review). It may also reflect that Positive Action included classroom, whole-school and external domain strategies delivered over multiple school years. Though Gatehouse[28] was similar to Positive Action in its focus on multiple systems, Gatehouse targeted adolescents, whereas Positive Action was delivered from KS2 and also included work with parents. Another possible explanation for our results is that effects for these interventions may take time to emerge. This is plausible given the developmental focus of many of these interventions, and evidence of links between early aggressive behaviour and later violence.[4 5] For example, there was some evidence that effects on aggressive behaviour in 4Rs began to emerge after the second intervention year.[19] While findings were somewhat

For peer review only

contradictory across different outcomes for PATHS, there was some evidence that teachers of intervention students reported less aggression in later years of the intervention.[33]

This systematic review has strengths and limitations. Identifying relevant studies was challenging often because of poor intervention description. We were unable to undertake meta-analysis or assessment of publication bias, though the preponderance of null results suggests that projects with non-significant findings are being published. Finally, the diversity of outcome measures and of raters precludes a complete and consistent picture of the effectiveness of these interventions via standardised measures. This is especially important as ‘core outcome sets’ become relevant in planning evaluations in public health and social science. Most studies focused on bullying, while evaluations of Positive Action[37 39] generally provided the most direct test of violent behaviours specifically.

Future research should seek to understand better the life course aspects of these interventions: that is, how does early school-based intervention impact later-life violent behaviours? From a policy perspective, it is clear that the integration of academic and health education, while possibly an effective intervention, will need to be considered alongside interventions involving other systems to prevent violence. Future evaluations will also contribute by considering the effects of integration in a diversity of ways and mechanisms of action for integration in different types of academic education. For example, contrasts between full and partial integration, which included evaluations did not address, could inform an understanding of how much integration is necessary to support health education messages.

FUNDING

This work was funded through National Institute for Health Research Public Health Research Programme grant 14/52/15.

The work was undertaken with the support of The Centre for the Development and Evaluation of Complex Interventions for Public Health Improvement (DECIPHer), a UKCRC

For peer review only

Public Health Research Centre of Excellence. Joint funding (MR/KO232331/1) from the British Heart Foundation, Cancer Research UK, Economic and Social Research Council, Medical Research Council, the Welsh Government and the Wellcome Trust, under the auspices of the UK Clinical Research Collaboration, is gratefully acknowledged.

COMPETING INTERESTS

Authors have no competing interests to disclose.

CONTRIBUTIONS

GJMT undertook study screening and selection, led the meta-analyses and drafted the initial manuscript. TT undertook study screening and selection, extracted data and contributed to drafting the initial manuscript. AF undertook study screening and selection. JT and RC provided methodological and substantive advice. CB undertook study screening and selection, extracted data, and contributed to drafting the initial manuscript. All authors revised the manuscript and approved the final manuscript as submitted.

The authors acknowledge Ms Claire Stansfield for her assistance in designing and conducting the searches.

DATA SHARING STATEMENT

All data are publicly available.

For peer review only

References

1. Krug EG, Mercy JA, Dahlberg LL, et al. The world report on violence and health. *The Lancet* 2002;360(9339):1083-88.
2. Scott S, Knapp M, Henderson J, et al. Financial cost of social exclusion: follow up study of antisocial children into adulthood. *BMJ* 2001;323(7306):191.
3. Beinart S, Anderson B, Lee S, et al. Youth at Risk?: A National Survey of Risk Factors, Protective Factors and Problem Behaviour among Young People in England, Scotland and Wales (JRF Findings 432). York: Joseph Rowntree Foundation 2002.
4. Bender D, Lösel F. Bullying at school as a predictor of delinquency, violence and other anti-social behaviour in adulthood. *Criminal Behaviour and Mental Health* 2011;21(2):99-106.
5. Olweus D. Bullying at school: What we know and what can we do. *Malden, MA: Blackwell* 1993
6. Farrington DP, Ttofi MM. School-based programs to reduce bullying and victimization: Campbell Systematic Reviews 2010.
7. Hahn R, Fuqua-Whitley D, Wethington H, et al. Effectiveness of universal school-based programs to prevent violent and aggressive behavior: A systematic review. *American Journal of Preventive Medicine* 2007;33(2):S114-S29.
8. Vreeman RC, Carroll AE. A systematic review of school-based interventions to prevent bullying. *Archives of Pediatrics & Adolescent Medicine* 2007;161(1):78-88.
9. Fletcher A, Bonell C, Sorhaindo A. "We don't have no drugs education": the myth of universal drugs education in English secondary schools? *International Journal of Drug Policy* 2010;21(6):452-8.
10. NASUWT. English Baccalaureate Survey Summary. Birmingham: NASUWT 2011.
11. PSHE Association. Comments on the National Curriculum proposals published in February 2013 from the PSHE education Strategic Partners' Group. PHSEA2013.
12. The Office for Standards in Education Children's Services and Skills. School inspection handbook: Handbook for inspecting schools in England under section 5 of the Education Act 2005. Manchester: The Government of the United Kingdom, 2016.
13. Bier MC, Zwarun L, Warren VF. Getting Universal Primary Tobacco Use Prevention Into Priority Area Schools A Media Literacy Approach. *Health Promotion Practice* 2011;12(6 suppl 2):152S-58S.
14. Pearson M, Chilton R, Wyatt K, et al. Implementing health promotion programmes in schools: a realist systematic review of research and experience in the United Kingdom. *Implementation Science* 2015;10(1):1.
15. Jones SM, Brown JL, Lawrence Aber J. Two-year impacts of a universal school-based social-emotional and literacy intervention: An experiment in translational developmental research. *Child Development* 2011;82(2):533-54.
16. Masten AS, Cicchetti D. Developmental cascades. *Development and Psychopathology* 2010;22(03):491-95.
17. Goleman D. Emotional intelligence. New York: Bantam 1995.
18. Jones SM, Brown JL, Aber JL. Three Year Cumulative Impacts of the 4Rs Program on Children's Social-Emotional, Behavioral, and Academic Outcomes. *Society for Research on Educational Effectiveness* 2010
19. Jones SM, Brown JL, Hoglund WL, et al. A school-randomized clinical trial of an integrated social–emotional learning and literacy intervention: Impacts after 1 school year. *Journal of Consulting and Clinical Psychology* 2010;78(6):829.

For peer review only

20. The British Heart Foundation. Money to Burn lesson plan 2014 [cited 2017 11 July]. Available from: http://teachers.theguardian.com/teacher-resources/15410/Money-to-burn----lesson-plan.
21. Wright G, Ainsworth P. Plastered evaluation: part of It's Not OK! Violence Prevention Education Programme. Liverpool: Ariel Trust, 2008.
22. Flay BR, Graumlich S, Segawa E, et al. Effects of 2 prevention programs on high-risk behaviors among African American youth: a randomized trial. *Archives of Pediatrics & Adolescent Medicine* 2004;158(4):377-84.
23. Kupersmidt JB, Scull TM, Benson JW. Improving media message interpretation processing skills to promote healthy decision making about substance use: the effects of the middle school media ready curriculum. *Journal of Health Communication* 2012;17(5):546-63.
24. Patton G, Bond L, Carlin JB, et al. Promoting social inclusion in schools: group-randomized trial of effects on student health risk behaviour and well-being. *American Journal of Public Health* 2006;96(9):1582-87.
25. Peersman G, Oliver S, Oakley A. EPPI-Center review guidelines: data collection for the EPIC database. London: EPPI-Centre Social Science Research Unit, 1997.
26. Higgins JPT, Green S. Cochrane Handbook for Systematic Reviews of Interventions Version 5.1.0 [updated March 2011]. Oxford: The Cochrane Collaboration 2011.
27. Sutcliffe K, Thomas J, Stokes G, et al. Intervention component analysis (ICA): a pragmatic approach for identifying the critical features of complex interventions. *Systematic Reviews* 2015;4(1):140.
28. Bond L, Patton G, Glover S, et al. The Gatehouse Project: can a multilevel school intervention affect emotional wellbeing and health risk behaviours? *Journal of Epidemiology and Community Health* 2004;58(12):997-1003. doi: 10.1136/jech.2003.009449
29. Couch L. The Bullying Literature Project: An Evaluation of a Class-Wide Bullying Intervention Program. University of California Riverside, 2015.
30. Jenson JM, Dieterich WA. Effects of a skills-based prevention program on bullying and bully victimization among elementary school children. *Prevention Science* 2007;8(4):285-96.
31. Li K-K, Washburn I, DuBois DL, et al. Effects of the Positive Action programme on problem behaviours in elementary school students: A matched-pair randomised control trial in Chicago. *Psychology and Health* 2011;26(2):187-204.
32. Frey KS, Hirschstein MK, Snell JL, et al. Reducing playground bullying and supporting beliefs: an experimental trial of the steps to respect program. *Developmental Psychology* 2005;41(3):479.
33. Crean HF, Johnson DB. Promoting Alternative Thinking Strategies (PATHS) and elementary school aged children's aggression: Results from a cluster randomized trial. *American Journal of Community Psychology* 2013;52(1-2):56-72.
34. Reid JB, Eddy JM, Fetrow RA, et al. Description and immediate impacts of a preventive intervention for conduct problems. *American Journal of Community Psychology* 1999;27(4):483-518.
35. Langford R, Bonell CP, Jones HE, et al. The WHO Health Promoting School framework for improving the health and well-being of students and their academic achievement. *The Cochrane Database of Systematic Reviews* 2014;4(4):CD008958.
36. Brown EC, Low S, Smith BH, et al. Outcomes from a school-randomized controlled trial of steps to respect: A bullying prevention program. *School Psychology Review* 2011;40(3):423.

For peer review only

37. Beets MW, Flay BR, Vuchinich S, et al. Use of a social and character development program to prevent substance use, violent behaviors, and sexual activity among elementary-school students in Hawaii. *American Journal of Public Health* 2009;99(8):1438-45.
38. Jenson JM, Brisson D, Bender KA, et al. Effects of the Youth Matters prevention program on patterns of bullying and victimization in elementary and middle school. *Social Work Research* 2013:svt030.
39. Lewis KM, Schure MB, Bavarian N, et al. Problem behavior and urban, low-income youth: A randomized controlled trial of Positive Action in Chicago. *American Journal of Preventive Medicine* 2013;44(6):622-30.
40. Jenson JM, Dieterich WA, Brisson D, et al. Preventing childhood bullying: Findings and lessons from the Denver public schools trial. *Research on Social Work Practice* 2010

For peer review only

Figures

Figure 1. PRISMA flowchart

For peer review only

Table 1. Characteristics of included studies

Evaluation, setting and studies	Sample characteristics	Intervention description	Control description
**Bullying Literature Project**<br><br>California, USA<br><br>Couch 2015[29]	4 classrooms, 95 teacher reports, 90 students (IG); 3 classrooms, 55 teacher reports, 42 students (CG)<br><br>Students enrolled in years 4 and 5, followed up one week post-intervention<br><br>42.8% female, 57.2% male<br><br>9.6% African American, 63.3% Hispanic, 9.0% Caucasian, 3.0% Asian, 4.2% other, 10.2% did not report<br><br>>50% of students received free or reduced-cost lunch	This intervention aims to reduce bullying by introducing themes related to bullying through children's literature. It also provides an opportunity for children to role-model practical skills to address or avoid bullying. The Bullying Literature Project integrates themes related to bullying into the children's literature used within a standard English curriculum. Students then had the opportunity to practice and reinforce skills via writing activities. The intervention was delivered by school psychologists supervised by the PI.	Waitlist control
**Gatehouse**<br><br>Melbourne, Australia<br><br>Bond 2004[28]	2 districts, 12 schools, 1335 students (IG); 2 districts, 14 schools, 1343 students (CG)<br><br>Students enrolled in Year 9, followed up for three years<br><br>53.2% female, 46.8% male<br><br>87.5% Australian-born<br><br>79.2% from two-parent family; 24.2% speak language other than English at home	Through teaching a curriculum (including integration of cognitive behavioural principles in English classes) and establishing a school-wide adolescent health team, Gatehouse aims to: build a sense of security and trust in students; enhance skills and opportunities for good communication; and build a sense of positive regard through participation in school life. The intervention was delivered by teachers, supported by the schoolwide adolescent health team and by external consultants who themselves were experiences teachers. Integration was achieved by using English classes to convey cognitive behavioural techniques for self-management, including via a 'critical literacy' approach that uses poetry, literature,	Education as usual

For peer review only

		song, film and visual materials.	
**Linking the Interests of Families and Teachers (LIFT)**<br><br>Pacific Northwest, USA<br><br>Reid 1999[34]	3 schools, 214 students (IG); 3 schools, 147 students (CG)<br><br>Students enrolled in year 6 and followed up over seven years<br><br>49% female, 51% male<br><br>86% White, 14% ethnic minority<br><br>12% mother less than high school graduate, 8% father less than high school graduate; 36% mother unemployed, 10% father unemployed; 22% single-parent families; 18% receiving benefits; 20% less than $15,000/year in early 1990s	Classroom instruction and discussion on specific social and problem-solving skills followed by skills practice, reinforced during free play using a group cooperation game with review of behaviour and presentation of daily rewards. There is also a parent evening to engage families and opportunities for parents to engage with teachers. The intervention was delivered by teachers and special instructors. Integration was achieved by teaching study skills alongside social-emotional education content.	Education as usual
**Positive Action Chicago**<br><br>Chicago, USA<br><br>Li 2011[31]<br>Lewis 2013[39]	7 schools, ~240 students (IG); 7 schools, ~260 students (CG)<br><br>Students enrolled in year 4 and followed up over six years<br><br>~48% female, ~52% male<br><br>55% African American, 32% Hispanic, 9% White non-Hispanic, 4% Asian, 5% other or mixed<br><br>83% receiving free lunch	Teachers provide lessons covering six units: self-concept; positive actions for mind and body; positive social-emotional actions; managing oneself; being honest with oneself; and continually improving oneself. Content includes 140 lessons per grade per year from years 1 to 13. In addition, an implementation coordinator and school climate team are appointed to support the intervention. The intervention is primarily delivered by teachers and school staff; in both trials, this was supported by extensive professional development and training. Integration was achieved by linking academic learning to social-emotional and health-related learning, e.g. by including content on problem solving and study skills alongside positive actions for mind and body, and by encouraging teachers to reflect Positive Action content in academic lessons.	Education as usual
**Positive Action Hawaii**	10 schools, 976 students (IG); 10 schools, 738 students (CG)		Education as usual

For peer review only

Hawaii, USA<br><br>Beets 2009[37]	Students enrolled in years 2 or 3 and followed up over four or five years<br><br>50% female, 50% male<br><br>26.1% Hawaiian, 22.6% mixed, 8.6% White, 1.6% African American, 1.7% American Indian, 4.7% other Pacific Islander, 4.6% Japanese, 20.6% other Asian, other 7.8%, unknown 1.6%.<br><br>Control schools had on average 55% free/reduced lunch students, whereas intervention schools had on average 56% free/reduced lunch students		
**Promoting Alternative Thinking Strategies (PATHS)**<br><br>Minnesota and New York State, USA<br><br>Crean 2013[33]	7 schools, 422 students (IG); 7 schools, 357 students (CG)<br><br>Students enrolled in year 4 and followed up over three years<br><br>57% female, 43% male<br><br>51% White, 38% African American, 10% other, 17% Hispanic<br><br>33% from single parent homes; 39% families with income less than $20,000/year, 43% below the federal poverty line; 11% no parent with high school diploma	An intervention to reduce conflict by improving students' social-emotional and thinking skills through a curriculum (including study skills), the establishment of a positive classroom environment and generalised positive social norms throughout the school environment. Lessons are grouped into three units addressing readiness and self-control, feelings and relationships, and interpersonal problem solving. These units cover five domains: 1. Self-control; 2. Emotional understanding; 3. Positive self-esteem; 4. Healthy relationships; and 5. Interpersonal problem-solving skills. The intervention is delivered by teachers supported by consultants, with 131 lessons delivered over three years (two to three times per week, 20 to 30 minutes each). Integration was achieved by linking study skills to social-emotional learning, by supporting teachers to include children's literature in reinforcing	Education as usual

For peer review only

		concepts, and by providing ideas to link PATHS to English, social studies and history lessons.	
**Reading, Writing, Respect and Reconciliation (4Rs)**<br><br>New York City, USA<br><br>Jones 2010[19]<br>Jones 2011[15]	9 schools, 515 students (IG); 9 schools, 427 students (CG)<br><br>Students enrolled in year 4 followed up for three years (only results up to two years available)<br><br>51.2% female, 48.8% male<br><br>41.1% African American, 45.6% Hispanic, 4.7% Caucasian, 8.6% other<br><br>31% low parental education, 15.1% parental unemployment, 53.4% single-parent household, 61.8% living in poverty	This intervention includes two components: 1. a seven-unit, 21–35 lesson literacy-based curriculum in conflict resolution and social-emotional learning for children in primary school (from year 1 to year 6); and 2. intensive professional development for teachers. The intervention was delivered by teachers after this extensive professional development. Integration was achieved by using literature as a springboard to help students understand anger and develop skills in listening, cooperation, assertiveness and negotiation.	Education as usual
**Steps to Respect I**<br><br>Pacific Northwest, USA<br><br>Frey 2005[32]	3 schools (IG), 3 schools (CG); 1,126 students total<br><br>Students enrolled in years 4 through 7; followed up for one year in endpoint-based analyses<br><br>49.4% female, 50.6% male<br><br>70.0% White, 9% African American, 12.7% Asian, 7.0% Hispanic, 1.3% Native American<br><br>SES indices not stated	This is an anti-bullying intervention with both school-wide and classroom components. The School-wide components create new disciplinary policies for bullying and improve monitoring of and intervention in bullying. Classroom curricula positive social norms and improve social–emotional skills for better engagement with bullying. The intervention was delivered by classroom teachers alongside schoolwide bullying policy teams. Biweekly lessons in the Steps to Respect curriculum are supported by 8-to-10 literature-based lessons presented over a 12 to 14 week period. This intervention integrates academic and health education by developing literacy skills alongside furthering understanding of the Steps to Respect curricular themes.	Waitlist control
**Steps to Respect II**	17 schools (IG), 16 schools (CG); 2,940		Waitlist

For peer review only

North-Central California, USA<br><br>Brown 2011[36]	students total<br><br>Students enrolled in years 4 through 6; followed up for one year<br><br>51% female, 49% male (IG); 48% female, 52% male (CG)<br><br>52% White, 7% African American, 6% Asian, 43% Hispanic, 35% other or mixed race (IG); 53% White, 6% African American, 6% Asian, 41% Hispanic, 35% other or mixed race (CG)<br><br>School-level average of 40% on free or reduced-price lunch		control
**Youth Matters**<br><br>Denver, USA<br><br>Jenson 2007[30]<br>Jenson 2010[40]<br>Jenson 2013[38]	14 schools, 702 students (IG), 14 schools, 462 students (CG)<br><br>Students enrolled in year 5 and followed up for three years<br><br>50.6% female, 49.4% male<br><br>59.1% Latinx, 14.7% African American, 16.8% American Indian, Asian American, or mixed, 9.3% Caucasian<br><br>SES indices not reported	Youth Matters promotes the development of healthy relationships and social competency and the development of social resistance. Classroom discussions around social issues promote positive social norms. Over four modules with 10 lessons, delivered over two years, students read age-appropriate stories, receive social-emotional learning and practice skills. The intervention was delivered by educational specialists from outside the school. Integration was achieved by using 30-40 page stories in each module intended to support schools in meeting academic standards in academic and health education.	Education as usual

Table 2. Appraisal of included studies

Intervention name	Random generation of allocation sequence	Concealed allocation	Blinding	Complete outcome data	Reporting not selective	Controlled for confounding	Accounted for clustering	Reduced other forms of bias	Suitable control group
Bullying Literature Project	NC	NS	NS	Yes	NC	NC	NS	NS	NC
Linking the Interests of Families and Teachers (LIFT)	Yes	NS	Yes	Yes	NC	Yes	Yes	Yes	NC
Positive Action Hawaii	NC	NS	NS	Yes	NC	NC	Yes	Yes	Yes
Positive Action Chicago	Yes	NS	NS	Yes	NC	Yes	Yes	Yes	Yes
Promoting Alternative Thinking Strategies (PATHS)	NC	NS	NS	Yes	NC	NC	Yes	Yes	NC
Reading Writing, Respect and Resolution (4Rs)	Yes	NS	NS	Yes	No	Yes	Yes	Yes	Yes
Steps to Respect I	NC	NS	NS	Yes	NC	NC	Yes	Yes	NC
Steps to Respect II	NC	NS	NS	Yes	NC	NC	Yes	Yes	Yes
Gatehouse	NC	NS	NS	Yes	NC	Yes	Yes	Yes	Yes
Youth Matters	NC	NS	NS	Yes	NC	Yes	Yes	NS	Yes

Legend: NC = not clear; NS = not stated

For peer review only

Table 3. Key themes in the intervention components analysis.

Key theme	Components within theme
Approach to integration	Literature: did interventions use literature and language arts as the key vehicle for delivery?<br>Local development: did interventions support teachers to link health education across academic subjects in each school?<br>Linking to developmental concerns: did interventions link academic education and personal health and development?
Domains of integration	Classroom: did interventions focus on the classroom?<br>Classroom and whole-school: did interventions include whole-school change components alongside classroom components?<br>Classroom, whole-school and external domains: did interventions also include parent engagement alongside classroom and whole-school components?
Degree of integration	Did interventions include full or partial integration of health education alongside academic education?
Timing of integration	Were interventions one year or multiple years in duration?

For peer review only

Table 4. Measures used in included studies

Evaluation	Measure	Notes
**Violence perpetration**		
4Rs	Aggression	Frequency score on 13 aggressive behaviours assessed by teacher report in last month, including physical aggression and threatening of others
Bullying Literature Project	Physical bullying	Assessed by teacher and student report; mean of frequency scores relating to reports of violence
LIFT	Change in child physical playground aggression	Measured by observation; includes physical bullying by observed children
PATHS	Aggression	Assessed by teacher and student report; mean of frequency scores relating to verbal and physical aggression
Positive Action Chicago	Bullying<br><br><br>Violence-related behaviours	Student report: mean of frequency scores relating to verbal or physical aggression behaviours in the past two weeks<br>Parent report: count of observed verbal or physical aggression behaviours in past 30 days<br>Count of lifetime behaviours: carried a knife, threatened to cut or stab someone, cut of stabbed someone on purpose, been asked to join a gang, hung out with gang members, been a member of a gang
Positive Action Hawaii	Cut or stabbed others<br>Shot another person<br>Physically hurts others<br>Gets into a lot of fights	Student report, lifetime prevalence<br>Student report, lifetime prevalence<br>Teacher report<br>Teacher report
Steps to Respect I	Bullying<br>Direct aggression	Playground observation of students<br>Mean of student reported frequency scores of direct bullying
Steps to Respect II	Bullying perpetration<br>Physical bullying perpetration	Measured by student report; proportion of students with at least one bullying behaviour<br>Measured by teacher report; proportion of students with at least one physical bullying behaviour
Youth Matters	Bullying<br>Bully, victim, or bully-victim	At least two or three times a month on at least one bullying behaviour<br>Classification of students based on questionnaire responses into one of three categories
**Violence victimisation**		
Bullying	Physical bullying	Assessed by teacher and student report; mean of frequency scores relating to reports of

For peer review only

Literature Project		violence
Gatehouse	Bullying victimisation	Assessed by student report; any of being teased, having rumours spread about them, deliberate exclusion or experience of threats or violence
PATHS	Victimisation	Assessed by student report; sum of frequency scores of victimisation in last two weeks
Steps to Respect I	Target of bullying	Playground observation of students
	Victimisation	Assessed by student report; mean of frequency scores for physical and verbal victimisation items
Steps to Respect II	Victimisation	Assessed by student report; mean of frequency scores for physical and verbal victimisation items
Youth Matters	Victimisation	Assessed by student report; mean of frequency scores for physical and verbal victimisation items, and also at least two or three times a month victimisation at least one bullying behaviour
	Bully, victim, or bully-victim	Classification of students based on questionnaire responses into one of three categories

For peer review only

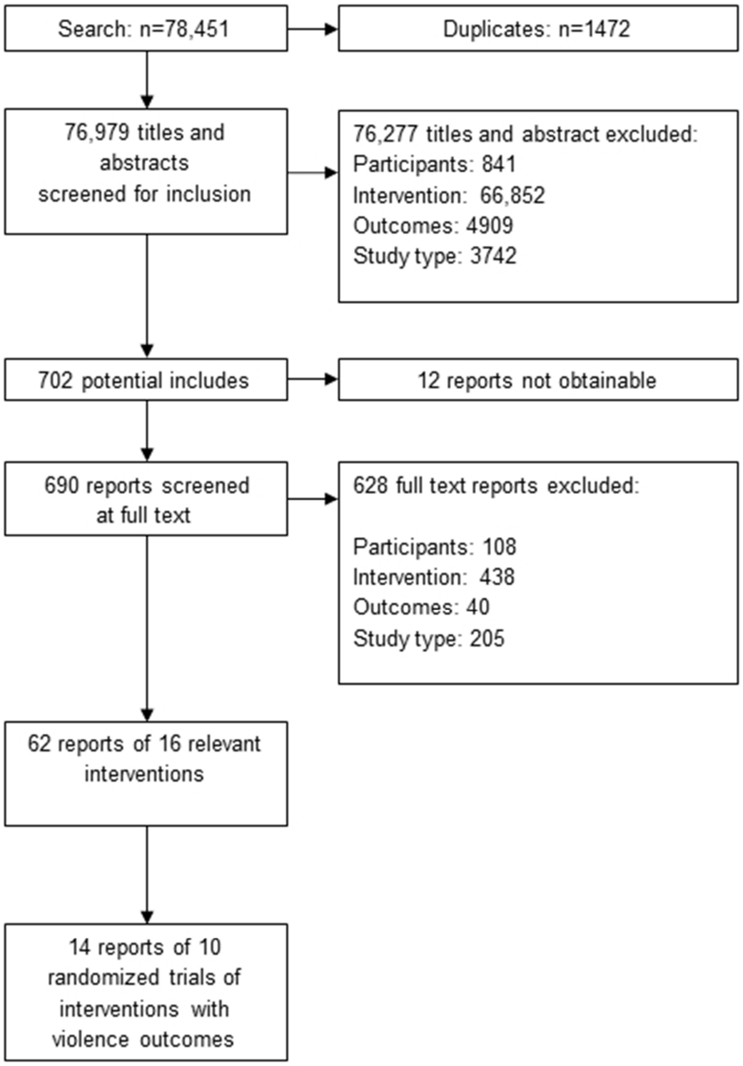

PRISMA flowchart

153x190mm (96 x 96 DPI)

Additional search and synthesis methods

We searched 19 electronic databases. The original list of databases was amended after consultation with our information scientist, as informed by initial searches.

- ASSIA via Proquest
- Australian Educational Index via Proquest
- BiblioMap (Database of health promotion research) via EPPI-Centre
- British Educational Index via EBSCOhost
- Cochrane Central Register of Controlled Trials via the Cochrane Library
- Cochrane Database of Systematic Reviews via the Cochrane Library
- Database of Abstracts of Reviews of Effects via the Cochrane Library
- Database of Promoting Health Effectiveness Reviews (DoPHER) via EPPI-Centre
- Dissertation Abstracts (UK theses, all dates; global theses 2010-2015) via Proquest
- Econlit via EBSCO
- Educational Research Index Citations via EBSCO
- Health Technology Assessment Database via the Cochrane Library
- International Bibliography of the Social Sciences via Proquest
- MEDLINE via OVID
- NHS Economic Evaluation Database
- PsycINFO via OVIDa
- Social Policy and Practice Including Child Data & Social Care Online via OVID
- Social Science Citation Index via Web of Knowledge
- Trials Register of Promoting Health Interventions via EPPI-Centre

We also searched the following 32 websites:

- Cambridge Journals
- Centers for Disease Control and Prevention: Smoking & Tobacco Use
- Child and Adolescent Research Unit
- Childhoods Today
- Children in Scotland
- Children in Wales
- Community Research and Development Information Service
- Database of Educational Research (EPPI-Centre)
- Drug and Alcohol Findings Effectiveness Bank
- Google
- Google Scholar
- Government of Wales
- Government of Scotland
- Joseph Rowntree Foundation
- National Criminal Justice Reference Service
- National Society of the Prevention of Cruelty to Children
- National Youth Agency
- Northern Ireland Executive
- OpenGrey
- Personal Social Services Research Unit
- Project Cork
- UCL-IOE Digital Education Resource Archive
- UK Clinical Research Net Study Portfolio
- University of Illinois at Urbana Champaign
- US Centre for Substance Abuse Prevention
- Social Issues Research Centre
- The Campbell Library
- The Children's Society
- The Open Library
- The Schools and Students' Health Education Unit Archive
- WHO International Clinical Trials Registry Platform
- Young Minds: Child & Adolescent Mental Health

For peer review only

PsycINFO search string

1. ((substance? or drug? or drinking or alcohol* or solvent?) adj1 ("use" or abus* or misuse*)).ti,ab.
2. ((substance? or drug? or drinking or alcohol* or solvent?) adj1 (usage or intake or using or taking or behavio* or user?)).ti,ab.
3. (drinking adj1 (alcohol* or behavio*)).ti,ab.
4. Alcohol.ti,ab.
5. (smoke or smoking or tobacco or cigarette? or smoker? or cannabis or marijuana).ti,ab.
6. (aggression or aggressive or bully* or delinquen* or "conduct problem*" or "conduct disorder?" or "antisocial" or "anti social" or violence or violent or (volatile adj behavio*) or victimi* or hostile or hostility or perpetrat*).ti,ab.
7. (Externalising or externalizing).ti,ab.
8. emotion*.ti,ab.
9. PSHE.ti,ab.
10. ("Health literacy" or "health education" or "health promotion" or "preventive health" or "primary prevention" or "health information" or "promoting health" or "health promoting" or "health promotion" or "health maintenance").ti,ab.
11. "Public health".ti,ab.
12. ("wellbeing" or "well being").ti,ab.
13. "mental health".ti,ab.
14. 1 or 2 or 3 or 4 or 5 or 6 or 7 or 8 or 9 or 10 or 11 or 12 or 13
15. ((curric* or lesson? or classes or classroom? or subject? or intervention? or program* or education or initiative? or learn or learning or teach or teaching or outcome* or attainment or achievement or assessment or effect* or impact* or score? or scoring* or skill? or knowledge or competen* or performance) adj3 (Academic or academically or Scholastic or scholar* or Mainstream or "main stream")).ti,ab.
16. ((curric* or lesson? or classes or classroom? or subject? or learn or learning or teach or teaching or attainment or achievement or assessment or score? or scoring* or skill? or knowledge or competen* or performance) adj3 School?).ti,ab.
17. ((intervention? or program* or initiative? or effect* or impact* or education) adj1 School?).ti,ab.
18. (class adj1 (Academic or academically or Scholastic or scholar* or School? or Mainstream or "main stream")).ti,ab.
19. ((curric* or lesson? or classes or classroom? or subject? or education or learn or learning or teach or teaching or attainment or achievement or score? or scoring* or skill? or knowledge or competen*) adj3 (study or core or generic)).ti,ab.
20. (class adj1 (study or core or generic)).ti,ab.
21. ((curric* or lesson? or classes or classroom? or subject? or attainment or achievement or assessment or score? or scoring* or competenc* or performance) adj3 ((Education not ("patient education" or "continuing education")) or educational)).ti,ab.
22. (class adj1 ((Education not ("patient education" or "continuing education")) or educational)).ti,ab.
23. (outcome* adj1 (education or educational)).ti,ab.
24. ((curric* or lesson? or classroom? or classes or subject? or intervention? or program* or initiative? or education or teach* or outcome* or attainment or achievement or assessment or effect* or impact* or score? or scoring* or skill? or knowledge or competen* or performance) adj3 (learn or learning)).ti,ab.
25. (class adj1 (learn or learning)).ti,ab.
26. ((curric* or lesson? or classes or classroom or class or subject? or education or teach* or learning or teach or teaching or learn or attainment or achievement or assessment or score? or scoring* or skill? or knowledge or competen* or performance) adj3 (art or arts or math* or science? or humanities or chemistry or physics or language* or geography or (history not ("medical history" or "health history" or "familial history" or "family history")) or numeracy or (literacy not "health literacy") or grammar or grammer or reading or writing)).ti,ab.
27. (((curric* or lesson? or classroom or classes or subject? or skill?) adj3 literature) or "literature class").ti,ab.
28. ("Education reform" or "Instructional support" or "School reform" or "Classroom organi*" or (Commit* adj3 (school or education or learning)) or (Engag* adj3 (school or education or learning)) or "Character development" or "Whole school" or "School level" or "School wide" or schoolwide).ti,ab.
29. ((Comprehensive adj3 school) and (intervention? or program* or initiative? or outcome* or effect* or impact*)).ti,ab.
30. ((Integrat* or Combin* or Infuse or infused or infusion or sustainable) adj3 (curric* or lesson? or classes or classroom or syllabus or subject? or education or learn or learning or teach or teaching)).ti,ab.
31. (((Integrat* or Combin* or Infuse or infused or infusion or sustainable) adj3 (intervention* or program* or initiative*)) and school?).ti,ab.
32. ((school or education or core or generic or teaching or learning) adj3 syllabus).ti,ab.

For peer review only

33. 15 or 16 or 17 or 18 or 19 or 20 or 21 or 22 or 23 or 24 or 25 or 26 or 27 or 28 or 29 or 30 or 32
34. (child* or schoolchild* or youth* or "young people*" or "young person" or teen* or adolescen* or juvenile* or preadolescen* or boy? or girl?).ti,ab.
35. (curric* or lesson? or classes or classroom? or subject? or school? or syllabus or "junior high" or "senior high" or "junior education" or "elementary education" or "primary education").ti,ab.
36. 34 and 35
37. ("secondary school?" or "primary school?" or "comprehensive school?" or "school education" or "high school?" or "grammar school?" or "private school?" or "public school?" or "mainstream school*" or "compulsory education" or "statutory education" or "middle school?" or "junior school?" or "senior school?" or "primary education" or "secondary education" or "elementary school?" or "elementary education" or "mainstream education" or "compulsory school*" or "statutory school*" or "sixth form college?" or "post-16 education" or "junior high" or "senior high" or "reception class" or "post primary").ti,ab.
38. ((school? or junior? or elementary or senior? or primary or "sixth form" or grade) adj10 student?).ti,ab.
39. pupil?.ti,ab.
40. 36 or 37 or 38
41. (University or universities or freshmen or sophomore? or "higher education" or "tertiary education" or ((registrar* or workplace? or clinical or medical or nursing or nurse? or doctor? or continuing or adult? or patient?) adj1 (education or educating or profession* or student?)) or "professional education").ti.
42. 40 not 41
43. 14 and 33 and 42
44. "Elementary School Students"/ or "Intermediate School Students"/ or "Primary School Students"/ or "Middle School Students"/ or "High School Students"/ or "Junior High School Students"/ or "Kindergarten Students"/ or "High School Education"/ or "Middle School Education"/ or "Secondary Education"/ or "Junior High Schools"/ or "High Schools"/ or "Schools"/ or "Elementary Schools"/ or "Middle Schools"/
45. "Drug Abuse Prevention"/ or "Health Education"/ or "Drug Education"/ or "Health Promotion"/ or "Public Health"/ or "Health Promotion"/ or "Preventive Medicine"/ or Health behaviour/ or Harm reduction/ or Health literacy/ or exp Health screening/ or Primary Mental health prevention/ or Prevention/ or Public health/ or Lifestyle changes/ or Lifestyle/ or Health literacy/
46. "Tobacco Smoking"/ or "Smoking Cessation"/ or "Marijuana Usage"/ or "Drinking Behavior"/ or "Social Drinking"/ or "Binge Drinking"/ or "Underage Drinking"/ or "Alcohol Abuse"/ or "Alcohol Drinking Patterns"/ or "Alcohol Intoxication"/ or "Alcoholism"/ or "Heroin Addiction"/ or "Drug Addiction"/ or "Drug Dependency"/ or "Drug Usage"/ or "Inhalant Abuse"/ or "Drug Abuse"/ or "Glue Sniffing"/ or "Predelinquent Youth"/ or "Cyberbullying"/ or "School Violence"/ or "Teasing"/ or "Juvenile Delinquency"/ or "Physical Abuse"/ or "Verbal Abuse"/ or "Violence"/ or "Harassment"/ or "Antisocial Behavior"/ or "Bullying"/ or "Perpetrators"/ or "Threat"/ or "Victimization"/ or "Relational Aggression"/ or "Aggressive Behavior"/ or "Behavior Problems"/ or "Behavior Disorders"/ or "Conduct Disorder"/ or "Drug Education"/ or "Drug Abuse Prevention"/ or "Harm Reduction"/
47. emotions/ or emotional development/
48. emotional adjustment/ or emotional disturbances/ or emotional control/
49. mental health/ or primary mental health prevention/ or well being/
50. "Curriculum"/ or "Curriculum Based Assessment"/ or "Curriculum Development"/ or "School Learning"/ or "Classroom Environment"/ or "Academic Environment"/ or "Teacher Effectiveness"/ or "Teacher Effectiveness Evaluation"/ or "Educational Program Evaluation"/ or "Course Evaluation"/ or "learning environment"/
51. 14 or 45 or 46 or 47 or 48 or 49
52. 33 or 50
53. 42 or 44
54. 51 and 52 and 53

For peer review only

PRISMA 2009 Checklist

Section/topic	#	Checklist item	Reported on page #
**TITLE**			
Title	1	Identify the report as a systematic review, meta-analysis, or both.	1
**ABSTRACT**			
Structured summary	2	Provide a structured summary including, as applicable: background; objectives; data sources; study eligibility criteria, participants, and interventions; study appraisal and synthesis methods; results; limitations; conclusions and implications of key findings; systematic review registration number.	2
**INTRODUCTION**			
Rationale	3	Describe the rationale for the review in the context of what is already known.	3-4
Objectives	4	Provide an explicit statement of questions being addressed with reference to participants, interventions, comparisons, outcomes, and study design (PICOS).	4
**METHODS**			
Protocol and registration	5	Indicate if a review protocol exists, if and where it can be accessed (e.g., Web address), and, if available, provide registration information including registration number.	4
Eligibility criteria	6	Specify study characteristics (e.g., PICOS, length of follow-up) and report characteristics (e.g., years considered, language, publication status) used as criteria for eligibility, giving rationale.	4
Information sources	7	Describe all information sources (e.g., databases with dates of coverage, contact with study authors to identify additional studies) in the search and date last searched.	Online File 1
Search	8	Present full electronic search strategy for at least one database, including any limits used, such that it could be repeated.	Online File 1
Study selection	9	State the process for selecting studies (i.e., screening, eligibility, included in systematic review, and, if applicable, included in the meta-analysis).	5
Data collection process	10	Describe method of data extraction from reports (e.g., piloted forms, independently, in duplicate) and any processes for obtaining and confirming data from investigators.	5
Data items	11	List and define all variables for which data were sought (e.g., PICOS, funding sources) and any assumptions and simplifications made.	Online File 1
Risk of bias in individual studies	12	Describe methods used for assessing risk of bias of individual studies (including specification of whether this was done at the study or outcome level), and how this information is to be used in any data synthesis.	5
Summary measures	13	State the principal summary measures (e.g., risk ratio, difference in means).	N/A
Synthesis of results	14	Describe the methods of handling data and combining results of studies, if done, including measures of consistency (e.g., I^2) for each meta-analysis.	5

For peer review only

PRISMA 2009 Checklist

Section/topic	#	Checklist item	Reported on page #
Risk of bias across studies	15	Specify any assessment of risk of bias that may affect the cumulative evidence (e.g., publication bias, selective reporting within studies).	5
Additional analyses	16	Describe methods of additional analyses (e.g., sensitivity or subgroup analyses, meta-regression), if done, indicating which were pre-specified.	5
**RESULTS**			
Study selection	17	Give numbers of studies screened, assessed for eligibility, and included in the review, with reasons for exclusions at each stage, ideally with a flow diagram.	6, Figure 1
Study characteristics	18	For each study, present characteristics for which data were extracted (e.g., study size, PICOS, follow-up period) and provide the citations.	6-7, Table 1
Risk of bias within studies	19	Present data on risk of bias of each study and, if available, any outcome level assessment (see item 12).	6, Table 2
Results of individual studies	20	For all outcomes considered (benefits or harms), present, for each study: (a) simple summary data for each intervention group (b) effect estimates and confidence intervals, ideally with a forest plot.	8-12
Synthesis of results	21	Present results of each meta-analysis done, including confidence intervals and measures of consistency.	N/A
Risk of bias across studies	22	Present results of any assessment of risk of bias across studies (see Item 15).	N/A
Additional analysis	23	Give results of additional analyses, if done (e.g., sensitivity or subgroup analyses, meta-regression [see Item 16]).	7-8
**DISCUSSION**			
Summary of evidence	24	Summarize the main findings including the strength of evidence for each main outcome; consider their relevance to key groups (e.g., healthcare providers, users, and policy makers).	12
Limitations	25	Discuss limitations at study and outcome level (e.g., risk of bias), and at review-level (e.g., incomplete retrieval of identified research, reporting bias).	12-13
Conclusions	26	Provide a general interpretation of the results in the context of other evidence, and implications for future research.	14
**FUNDING**			
Funding	27	Describe sources of funding for the systematic review and other support (e.g., supply of data); role of funders for the systematic review.	14

From: Moher D, Liberati A, Tetzlaff J, Altman DG, The PRISMA Group (2009). Preferred Reporting Items for Systematic Reviews and Meta-Analyses: The PRISMA Statement. PLoS Med 6(7): e1000097. doi:10.1371/journal.pmed1000097

For more information, visit: **www.prisma-statement.org**.

For peer review only - http://bmjopen.bmj.com/site/about/guidelines.xhtml

BMJ Open

Integration of academic and health education for the prevention of physical aggression and violence in young people: systematic review, narrative synthesis and intervention components analysis

Journal:	*BMJ Open*
Manuscript ID	bmjopen-2017-020793.R1
Article Type:	Research
Date Submitted by the Author:	01-May-2018
Complete List of Authors:	Melendez-Torres, G.J.; Cardiff University, DECIPHer, School of Social Sciences<br>Tancred, Tara; London School of Hygiene and Tropical Medicine, Department of Social and Environmental Health Research<br>Fletcher, Adam; British Heart Foundation Cymru<br>Campbell, Rona; University of Bristol, DEICPHer, Bristol Medical School<br>Thomas, James; University College London Social Science Research Unit<br>Bonell, Christopher; London School of Hygiene and Tropical Medicine, Department of Social and Environmental Health Research
**Primary Subject Heading**:	Public health
Secondary Subject Heading:	Paediatrics
Keywords:	systematic review, violence, Community child health < PAEDIATRICS

SCHOLARONE™ Manuscripts

For peer review only

Integration of academic and health education for the prevention of physical aggression and violence in young people: systematic review, narrative synthesis and intervention components analysis

Dr G.J. Melendez-Torres [1]*

Dr Tara Tancred [2]

Prof Adam Fletcher [3]

Prof Rona Campbell [4]

Prof James Thomas [5]

Prof Christopher Bonell [2]

[1] DECIPHer, School of Social Sciences, Cardiff University, Cardiff, UK

[2] Department of Social and Environmental Health Research, London School of Hygiene and Tropical Medicine, London, UK

[3] British Heart Foundation Cymru, Cardiff, UK

[4] DECIPHer, Bristol Medical School, University of Bristol, Bristol, UK

[5] EPPI-Centre, UCL Institute of Education, University College London, London, UK

*Corresponding author
G.J. Melendez-Torres
DECIPHer
School of Social Sciences
Cardiff University
1-3 Museum Place
Cardiff CF10 3BD
UNITED KINGDOM
melendez-torresg@cardiff.ac.uk
+44 (0) 29208 79106

Word count: 4,023

For peer review only

Abstract

Objectives. To systematically review evidence on the effectiveness of interventions including integration of academic and health education for reducing physical aggression and violence, and describe the content of these interventions.

Data sources. Between November and December 2015, we searched 19 databases and 32 websites and consulted key experts in the field. We updated our search in February 2018.

Eligibility criteria. We included randomised trials of school-based interventions integrating academic and health education in students aged 4-18 and not targeted at health-related subpopulations (e.g. learning or developmental difficulties). We included evaluations reporting a measure of interpersonal violence or aggression.

Data extraction and analysis. Data were extracted independently in duplicate, interventions were analysed to understand similarities and differences, and outcomes were narratively synthesised by key stage (KS).

Results. We included 10 evaluations of eight interventions were reported in 14 papers. Interventions included either full or partial integration, incorporated a variety of domains beyond the classroom, and used literature, local development or linking of study skills and health promoting skills. Evidence was concentrated in KS2, with few evaluations in KS3 or KS4, and evaluations had few consistent effects; evaluations in KS3 and KS4 did not suggest effectiveness.

Discussion. Integration of academic and health education may be a promising approach, but more evidence is needed. Future research should consider the 'lifecourse' aspects of these interventions; that is, do they have a longitudinal effect? Evaluations did not shed light on the value of different approaches to integration.

For peer review only

Strengths and limitations of this study

- We used an exhaustive search including 19 databases and 32 websites.
- We used an innovative method to describe key components in this class of interventions.
- However, it was challenging to identify studies for inclusion.
- Meta-analysis was not possible because of the diversity of outcomes and raters.

For peer review only

INTRODUCTION

Violence among young people is a public-health priority due to its prevalence and harms to young people and wider society.[1, 2] One UK study found that 10% of young people aged 11–12 reported carrying a weapon and 8% admitted attacking someone with intent to hurt them seriously.[3] By age 15–16, 24% of students reported they have carried a weapon and 19% reported attacking someone with the intention to hurt them seriously.[3] Early aggression and anti-social behaviour are strongly linked to adult violent behaviour.[4, 5]

School-based health education can be effective in reducing violence.[6-8] However, school-based health education is increasingly marginal in many high-income countries, partly because of schools increasing focus on attainment-based performance metrics. In England specifically, health education is not a statutory subject,[9-11] and school inspectors have a limited focus on how schools promote student health.[12]

One way to avoid such marginalisation is to integrate health education into academic lessons. For example, health-related content can be seamlessly integrated into existing academic lessons or discrete additional health education lessons can also include academic learning elements. This strategy may bring other benefits because: larger 'doses' may be delivered; students may be less resistant to health messages weaved into other subjects; and lessons in different subjects may reinforce each other.[13, 14] Conversely, those teaching academic subjects may be uninterested or unqualified to teach health topics. Though theories of change in this class of interventions are diffuse, one important way in which they could be effective is by promoting developmental cascades involving the interplay of cognitive and non-cognitive skills.[15, 16] Interventions integrating academic and health education could address violence by developing: social and emotional skills such as self-awareness, self-regulation, motivation, empathy and communication;[17] healthier social support or norms

For peer review only

among students[15, 18, 19]; knowledge of the costs [20] and consequences [21] of substance use; media literacy skills to critique harmful media messages; and modifying students' social norms about antisocial behaviours.[13, 20, 22-24] Our work synthesising the theories of change underlying these interventions (Tancred et al., in press) identified that interventions aimed to integrate and thus erode boundaries between academic and health education, between students and teachers (so that relationships were improved and teachers might function more effectively as behavioural role models), and between classrooms and schools and schools and families (so that violence prevention messages communicated in classrooms might be reinforced by messaging in other settings).

Despite policy interest in these interventions, they have not previously been the subject of a specific systematic review. Previous systematic reviews have focused on socio-emotional learning interventions or school-based interventions generally,[6-8] without considering interventions that specifically integrate with academic lessons as defined above. Our focus on violence is informed by preliminary consultation, scoping work and logic model development suggesting that violence is an outcome especially amenable to these interventions. In the present review, we examined the characteristics of interventions that integrate academic and health education to prevent violence, and synthesised evidence for their effectiveness. That is, our research questions were: what are the overarching features relevant to integration of interventions that integrate academic and health education, and are these interventions effective at different key stages in reducing physical aggression and violence?

METHODS

This review was part of a larger evidence synthesis project on theories of change, process evaluations and outcome evaluations of integration of academic and health education

For peer review only

for substance use and violence. We registered the protocol for this review on PROSPERO (CRD42015026464, https://www.crd.york.ac.uk/prospero/).

Inclusion and exclusion

Studies were included regardless of publication date or language. We included randomised controlled trials of interventions integrating academic and health education, the former defined as specific academic subjects or general study skills. We defined as ‘health education’ education seeking to improve the health and wellbeing of students (including social and emotional learning and other forms of violence prevention). We included school-based interventions that seamlessly incorporated health education into existing academic lessons and interventions that provided discrete health education lessons with additional academic components. Interventions could be delivered by teachers or other school staff such as teaching assistants, but may also have been delivered by external providers, for example from the health, voluntary or youth service sectors. We did not include interventions solely addressing social conduct in the classroom; relationships with peers or staff; attitudes to education, school or teachers; or aspirations and life goals. Our also definition excluded interventions which: were delivered in mainstream subject lessons but did not aim to integrate health and academic education; trained teachers in classroom management without student curriculum components; or were delivered exclusively outside of classrooms, as these did not seek to integrate academic and health education.

For this review, we focus on violence outcomes, defined as the perpetration or victimisation of physical violence including convictions for violent crime. We included outcomes that were a composite of physical and non-physical (e.g. emotional) interpersonal violence, but excluded composite measures that included items not focused on interpersonal violence, such as damage to property.

For peer review only - http://bmjopen.bmj.com/site/about/guidelines.xhtml

Interventions focusing on targeted health-related sub-populations (e.g. children with cognitive disabilities) were excluded as we were interested in universal interventions. We excluded interventions that trained teachers in classroom management.

Search strategy

In our original search, undertaken between November and December 2015, we searched 19 databases and 32 websites, and contacted subject experts (see Online File 1 for full details). We subsequently updated our search in February 2018 using PsycINFO and CENTRAL, as all of our original study hits were recovered from these databases.

Study selection

Pairs of researchers double-screened titles and abstracts in sets of 50 references until 90% agreement was reached, with disagreements discussed at every stage. Subsequently, single reviewers screened each reference. We located the full texts of remaining references and undertook similar pairwise calibration with disagreements discussed, followed by single screening. Reports were translated into English where necessary. Using an existing tool[25] we extracted data independently in duplicate from included studies and assessed trials for risk of bias using a modified version of the Cochrane assessment tool.[26] Authors were contacted where study data were missing.

Synthesis methods

We undertook an intervention components analysis.[27] This was undertaken inductively by one researcher and audited by two other researchers, and used intervention descriptions to draw out similarities and differences in intervention design using an iterative method. Intervention descriptions were read and re-read and then coded manually. The goal of this analysis was to use a set of descriptors to characterise aspects of the integration of academic and health education in the intervention. Intervention descriptions were rarely detailed enough to permit 'deep' engagement with the specific content of the interventions

For peer review only

provided in included interventions. The intervention components analysis identified key domains of relevance in understanding the integration of academic and health education, and developed within each domain a set of overlapping categories that described key differences between interventions within each domain. Finally, we synthesised outcomes narratively due to the heterogeneity in included outcome measurement. We categorised the timing of intervention effect by period of schooling, defined in terms of English schools' key-stage (KS) system. KS1 includes school years 1–2 (age 5–7 years); KS2 includes years 3–6 (age 7–11 years); KS3 includes years 7–9 (age 11–14 years); KS4 includes years 10–11 (age 14–16 years); and KS5 includes years 12–13 (age 16–18 years).

We could not formally assess publication bias because heterogeneity in outcome measurement precluded meta-analysis.

Patient and public involvement

Because this review focused on public health interventions that were generally preventive in nature, patients were not involved *per se*. However, stakeholders were extensively consulted in the development of research questions and in assessing the implications of the findings. In addition, findings were disseminated via stakeholder events, and a series of one-to-one consultations took place to ensure the relevance and salience of study findings.

RESULTS

In our original search, we found and screened 76,979 references, of which we retained 702 for full-text screening and were able to assess 690. Of 62 relevant reports included in the overall project, 10 evaluations of eight interventions were reported in 14 papers that considered violence and are reported in this review. Our update search yielded 2,355 references, of which we retained 41 for full-text screening and included six papers reporting three evaluations (Figure 1). This yielded a total of 13 evaluations reported in 20 papers.

For peer review only

Included studies and their quality

All trials randomised schools except the Bullying Literature Project, which randomised classrooms (Table 1). All evaluations were conducted in the USA, except for Gatehouse,[28] which was an Australian study. All control arms consisted of education-as-usual or waitlist controls, though Second Step[29-31] offered a brief anti-bullying intervention with low takeup.

Interventions were diverse and are summarised below in the intervention components analysis. Only two interventions (Bullying Literature Project,[32] Youth Matters[33]) were wholly delivered by external staff. Several (Gatehouse,[28] Positive Action,[34] Steps to Respect[35]) linked classroom-based delivery to school-level work to support and reinforce implementation. PATHS[36] and 4Rs[19] also emphasised teachers' professional development.

Evaluation quality varied (Table 2). Appraisal was hampered by poor reporting of some aspects of trial methods. Only four studies reported evidence of low risk of bias for random generation of allocation sequence; the remainder were unclear. Only one study reported information on concealed allocation. In LIFT,[37] outcome assessors were blinded, resulting in low risk of bias in this domain, but all other interventions were of unclear risk of bias. All interventions included reasonably complete outcome data, and in only one evaluation did unit of analysis issues pose a risk of bias. In some studies such as Steps to Respect, follow-up was shorter than intervention length. Evaluations also differed in size, ranging in size from seven classrooms to 63 schools.

Intervention components analysis

This identified four themes describing included interventions: approach to integration, position of integration, degree of integration and point of integration. Included interventions are described in Table 1, and the components analysis is summarised in Table 3.

For peer review only

Approach to integration

Interventions approached the rationale for and strategy of integration in different and overlapping ways. These overlapped across interventions, but were not mutually exclusive, and described the types of academic foci that interventions used to integrate academic and health education. Several (4Rs, Bullying Literature Project, Steps to Respect, Youth Matters) focused on *literature* as a focus for integration, using children's books as a prompt for social-emotional learning. These interventions targeted language arts or literacy lessons as an opportunity to provoke discussion, role-play and modelling of positive strategies to avoid violence. Gatehouse explicitly used a 'critical literacy' approach to inspire reflection on programme lessons in English classes. Another approach to integration emphasised *local development*, where interventions supported teachers to link health education across academic subjects in each school. For example, in PATHS, teachers received suggestions on how to integrate programme learning across English, history and social studies lessons, while in Second Step, this was an encouraged aspect of classroom delivery. A third approach was *linking to developmental concerns*, emphasising not so much the comprehensive integration of academic and health education but rather the interrelationships between academic success and broader development, health and wellbeing. These interventions viewed academic education through a 'health' lens, in addition to viewing health education through an 'academic' lens. Interventions either manifested this as part of their activities, for example by pairing study skills lessons with social-emotional learning (PATHS), or as part of their underlying theory of change. For example, Gatehouse sought to create healthy schools that would also support academic attainment, whereas Positive Action tied together individual student attainment with student health and wellbeing.

Domains of integration

For peer review only

Some interventions (4Rs, Bullying Literature Project and Youth Matters) were exclusively *classroom-focused* while others (Gatehouse, Steps to Respect) used *classroom and whole-school* strategies to reinforce and extend learning. For example, Gatehouse involved school implementation-support teams, while Steps to Respect deployed a schoolwide 'policy team' to revise and develop anti-bullying policies. Other interventions, (PATHS, Positive Action) used *classroom, whole school environment and external domain* (parent information) strategies consistent with the health promoting schools approach promulgated particularly by the WHO, which in the US is known as the Comprehensive School Health Program (CSHP) model.[38]

Degree of integration

In some interventions, health education was fully integrated (woven seamlessly) into everyday academic lessons (Gatehouse, 4Rs, Youth Matters), while in partially integrated interventions, health education involved distinct lessons, albeit also covering academic learning (Positive Action).

Timing of integration

Most interventions were multi-year, though two involved only one school year (LIFT, Bullying Literature Project).

Intervention effects

Perpetration measures included bullying (physical, or physical/verbal), aggression against peers and others, and violent behaviours including injuring others. Measures involved different raters, including students, teachers and observers. Victimisation measures ranged from physical violence specifically to interpersonal aggression more generally. Heterogeneity of definition, measurement and form of effect sizes precluded meta-analysis. No included studies described effects for KS1 or KS5. Measures and corresponding effect estimates are included in Table 4.

For peer review only

Violence perpetration: KS2

Across the 10 evaluations reporting outcomes in this KS, effects were inconsistent, including within studies by rater.

In LIFT,[37] effects at the end of the first intervention year on observed physical aggression in the playground were similar for students with different levels of baseline aggression(d=-0.14 at mean, 1 SD and 2 SD above the pre-intervention mean); these findings being described as 'statistically significant'. However, after the first intervention year of 4Rs,[19] there were no effects on teacher-reported aggression (regression-estimated b=0.02, SE=0.05, based on a 1-4 scale). After the second intervention year,[15] there were effects on teacher-reported student aggression (d=-0.21, p<0.05). The Bullying Literature Project also reported no effects on physical aggression rated by teachers for individual students (IG: M=1.12, SD=0.47, n=95 vs. CG: 1.19, SD=0.47, n=55; p=0.67) or student self-reports (1.20, 0.44, n=90 vs. 1.14, 0.36, n=42; p=0.84) at one week post-intervention.[32] This finding was the same in the Bullying Literature Project—Moral Disengagement version (F(1, 80)=0.83, p=.431), though only combined student-reported physical and emotional bullying estimates were available.[39]

Findings for Steps to Respect differed by type of rater. At the end of the first intervention year, the first evaluation of Steps to Respect[35] reported evidence of decreased bullying based on playground observation (F(91.3)=5.02, p<0.01) but not direct aggression based on student report (F(68.7)=2.05, p>0.05). The second evaluation of Steps to Respect[40] revealed a similar pattern. While teacher reports of physical bullying perpetration were less in intervention schools than in control schools at the end of the first intervention year (OR=0.61, t(29)=-3.12, p<0.01), student reports suggested no difference between schools on bullying perpetration (t(29)=-1.06). Moreover, in PATHS,[36] small positive effects of the intervention on student-reported aggression at the end of the first

intervention year (*d*=-0.048, 95% CI [-0.189, 0.092]) and at the start (-0.064, [-0.205, 0.076]) and end (-0.048, [-0.188, 0.093]) of the second intervention year gave way to a small deleterious intervention effect at the end of the third year (0.082, [-0.060, 0.224]). Opposite effects were found on teacher-reported aggression, with initially small, negative intervention effects at the end of the first (0.036, [-0.105, 0.178]) and start of the second intervention year (0.035, [-0.107, 0.178]) but progressively greater effects at the end of the second (-0.005, [-0.146, 0.136]) and the third (-0.199, [-0.338, -0.060]) intervention years.

In contrast, two evaluations showed consistently positive results across different measures. In Positive Action Chicago,[34] students reported lower counts of bullying behaviours (IRR [incidence rate ratio] =0.59, 95% CI [0.37, 0.92]) and of serious violence-related behaviours, including cutting or stabbing someone on purpose (0.63, [0.45, 0.88]). Findings from Positive Action Hawaii[41] were similar for student-reported violent behaviours (IRR=0.42, 90% CI [0.24, 0.73]) and teacher-reported violent behaviours (0.54, [0.30, 0.77]). For students in the fourth or fifth intervention year, intervention recipients were less likely to report cutting or stabbing someone (OR=0.29, 90% CI [0.16, 0.52]) or shooting someone (0.24, [0.14, 0.40]). Teachers were less likely to report that students hurt others (0.61, [0.38, 0.97]) or got into lots of fights (0.63, [0.47, 0.84]).

However, in Youth Matters,[33] students in intervention schools were not less likely to report bullying perpetration (OR=0.85, 95% CI [0.29, 1.47], *p*=0.585) after the second intervention year. Evaluators explored use of latent class analyses to classify intervention recipients as victims, bullies or bully-victims. Proportions of intervention and control recipients classified as bullies or bully-victims were not significantly different by study arm at the end of the first (IG: 21%, n=356 vs CG: 22%, n=392) or second (19%, n=244 vs 23%, n=293) intervention years.[42]

Violence perpetration: KS3

For peer review only

The three evaluations examining violence perpetration outcomes in KS3 had dissimilar results. At the end of the sixth intervention year of Positive Action Chicago,[43] students receiving the intervention reported lower counts of violence-related behaviours than no-treatment controls (IRR=0.38, 95% CI [0.18, 0.81]; equivalent to *d*=-0.54). Students also reported fewer bullying behaviours (*d*=-0.39), and parents reported that their children engaged in fewer bullying behaviours (*d*=-0.31). Significance values for these estimates were not presented, but both were supported by significant condition-by-time interactions in multilevel models, indicating that the intervention group showed an improved trajectory over time as compared to the control group. In contrast, after the third year from baseline in Youth Matters,[42] proportions of students were not different in the collective bully and bully-victim groups (both groups 16%; IG n=283, CG n=289). Findings for Second Step were reported at the end of the first, second and third years of intervention. At the end of the first school year, students in intervention schools had decreased odds of physical aggression (OR=0.70, $p<0.05$) but not sexual harassment and violence perpetration (OR=1.04, $p>0.05$).[31] These findings did not hold to the end of the second school year for physical aggression (OR=0.80, 95% CI [0.59, 1.08]), but sexual harassment and violence perpetration was significantly reduced in intervention schools in Illinois (0.72, [0.54, 0.95]) but not Kansas (0.99, [0.71, 1.48]).[30] At the end of the third school year, there were no direct effects of Second Step on sexual harassment perpetration (β=0.005, SE=0.012); findings for physical aggression were not available.[29]

Violence victimisation: KS2

While the seven evaluations reporting outcomes in this KS were similar in follow-up period, they did not point to a clear effect. Students receiving the 'original' Bullying Literature Project were not different from their peers in physical victimisation by teacher report on individual students (IG: M=1.04, SD=0.23, n=95 vs. CG: 1.04, SD=0.21, n=55;

For peer review only

p=0.39) or student self-report (1.35, 0.54, n=90 vs. 1.43, 0.66, n=42; *p*=0.57) one week post-intervention.[32] However, students receiving the Bullying Literature Project—Moral Disengagement version did report decrease in victimisation (both physical and emotional combined) after the intervention (IG: M=1.76, SD=0.81 to M=1.60, SD=0.66, n=42 vs. CG: M=1.23, SD=0.38 to M=1.38, SD=0.53, n=42), with a significant time-by-treatment interaction in an ANOVA (F(1, 80)=7.42, p=0.047).[39] PATHS measured student-reported victimisation using standardised mean differences, and found small, non-significant increases relative to the control arm at: the end of the first intervention year (*d*=0.044, 95% CI [-0.098, 0.185]); the start (0.074, [-0.067, 0.216]) and end (0.092, [-0.050, 0.234]) of the second year; and the end of the third year (0.089, [-0.053, 0.231]) of intervention implementation.[36] Steps to Respect, evaluated in two different trials, also found no differences in student-reported bullying victimisation at the end of the first intervention year in the first (IG: M=0.80, SD=1.51 vs CG: M=0.86, SD=1.44; *F*<1)[35] or second trial (2.11, 1.03 vs. 2.18, 1.06; *t*(29)=-1.15).[40] The first trial included playground observation at the end of the first intervention year, which was suggestive of lower levels in bullying victimisation, though these differences were marginally non-significant (0.9, 0.82 vs. 1.01, 0.83; *F*(72.4)=3.74, *p*<0.10).[35] Learning to Read in a Healing Classroom examined relational and physical victimisation after one year of intervention implementation and found no significant effect of the intervention (weighted *d*=-0.01, SE=0.06).[44, 45] Finally, Youth Matters examined bullying victimisation through continuous and dichotomous measures. At the end of the second intervention year, the difference in log-transformed continuous scores suggested a decrease (difference=-0.171, SE=0.083, *p*=0.049), as did the difference in dichotomous scores (OR=0.61, *p*=0.098).[33] However, the latent class analysis did not suggest a difference between groups at this point.[42]

Violence victimisation: KS3 and KS4

For peer review only

Intervention evaluations reporting violence victimisation outcomes in KS3 (Youth Matters [42, 46], Second Step[30, 31] and Gatehouse[28]) and KS4 (Gatehouse[28]) suggested no evidence of effectiveness. In Youth Matters, differences in the log-transformed scores for bullying victimisation suggested a decrease in victimisation in intervention recipients as compared to controls, but this difference was not significant (regression-estimated difference=-0.123, SE=0.068, p=0.08).[46] However, at the end of the third intervention year, fewer students in the intervention than control group were members of the victim or bully-victim classes (36%, n=283 vs 45%, n=289).[42] Based on our own chi-square test, this difference was significant (p=0.029). In Second Step, neither peer victimisation (OR=1.01, p>0.5) nor sexual harassment and violence victimisation (OR=1.01, p>0.05) were different between students in intervention schools and control schools after the first intervention year;[31] this remained the case at the end of the second intervention year (peer victimisation: OR=0.94, 95% CI [0.75, 1.18]; sexual victimisation: 0.91, [0.72, 1.15]).[30] Gatehouse,[28] which was implemented from year 9, found no evidence of a change in bullying victimisation at the end of the first (OR=1.03, 95% CI [0.86, 1.26]), second (1.03, [0.78, 1.34]) or third (0.88, [0.68, 1.13]) intervention years, which corresponded to the first two years of KS4.

DISCUSSION

While the integration of academic and health education remains a promising model for the delivery of school-based health education, randomised evaluations were variable in quality and did not consistently report evidence of effectiveness in reducing violence victimisation or perpetration. Evidence was concentrated in KS2, with few evaluations in KS3 or KS4.

Few interventions showed consistent signals of effectiveness. Though a formal moderator analysis was not possible, certain intervention models appear more effective than

For peer review only

others. Specifically, evaluations of Positive Action in both Chicago[43] and Hawaii[41] showed consistently positive results across diverse measures. This may reflect the involvement of the intervention developer, a factor often associated with improved intervention fidelity (although Positive Action was not unique in this respect among interventions included in our review). It may also reflect that Positive Action included classroom, whole-school and (in the Hawaii trial) external domain strategies delivered over multiple school years. Though Gatehouse[28] was similar to Positive Action in its focus on multiple systems, Gatehouse targeted adolescents, whereas Positive Action was delivered from KS2 and also included work with parents. Another possible explanation for our results is that effects for these interventions may take time to emerge. This is plausible given the developmental focus of many of these interventions, and evidence of links between early aggressive behaviour and later violence.[4, 5] For example, there was some evidence that effects on aggressive behaviour in 4Rs began to emerge after the second intervention year.[19] While findings were somewhat contradictory across different outcomes for PATHS, there was some evidence that teachers of intervention students reported less aggression in later years of the intervention.[36] Another key feature of Positive Action was the use of a model that linked academic and health education to developmental concerns, both in terms of activities as well as in the underlying theory of change. Moving forward, intervention strategies that combine multiple domains over several years and that use both subject-specific learning alongside linking to developmental concerns may be more effective than classroom-only interventions, single-year interventions, or interventions that use literature alone; this should be a target for future research.

This systematic review has strengths and limitations. Identifying relevant studies was challenging often because of poor intervention description. We were unable to undertake meta-analysis or assessment of publication bias, though the preponderance of null results

For peer review only

suggests that projects with non-significant findings are being published. Finally, the diversity of outcome measures and of raters precludes a complete and consistent picture of the effectiveness of these interventions via standardised measures. This is especially important as 'core outcome sets' become relevant in planning evaluations in public health and social science. Most studies focused on bullying, while evaluations of Positive Action[41, 43] generally provided the most direct test of violent behaviours specifically.

Future research should seek to understand better the life course aspects of these interventions: that is, how does early school-based intervention impact later-life violent behaviours? From a policy perspective, it is clear that the integration of academic and health education, while possibly an effective intervention, will need to be considered alongside interventions involving other systems to prevent violence. Future evaluations will also contribute by considering the effects of integration in a diversity of ways and mechanisms of action for integration in different types of academic education. For example, contrasts between full and partial integration, which included evaluations did not address, could inform an understanding of how much integration is necessary to support health education messages.

FUNDING

This work was funded through National Institute for Health Research Public Health Research Programme grant 14/52/15.

The work was undertaken with the support of The Centre for the Development and Evaluation of Complex Interventions for Public Health Improvement (DECIPHer), a UKCRC Public Health Research Centre of Excellence. Joint funding (MR/KO232331/1) from the British Heart Foundation, Cancer Research UK, Economic and Social Research Council, Medical Research Council, the Welsh Government and the Wellcome Trust, under the auspices of the UK Clinical Research Collaboration, is gratefully acknowledged.

COMPETING INTERESTS

For peer review only

Authors have no competing interests to disclose.

CONTRIBUTIONS

GJMT undertook study screening and selection, led the meta-analyses and drafted the initial manuscript. TT undertook study screening and selection, extracted data and contributed to drafting the initial manuscript. AF undertook study screening and selection. JT and RC provided methodological and substantive advice. CB undertook study screening and selection, extracted data, and contributed to drafting the initial manuscript. All authors revised the manuscript and approved the final manuscript as submitted.

The authors acknowledge Ms Claire Stansfield for her assistance in designing and conducting the searches.

DATA SHARING STATEMENT

All data are publicly available.

For peer review only

References

1 Krug EG, Mercy JA, Dahlberg LL*, et al.* The world report on violence and health. *The Lancet* 2002;**360**:1083-8.

2 Scott S, Knapp M, Henderson J*, et al.* Financial cost of social exclusion: follow up study of antisocial children into adulthood. *BMJ* 2001;**323**:191.

3 Beinart S, Anderson B, Lee S*, et al. Youth at Risk?: A National Survey of Risk Factors, Protective Factors and Problem Behaviour among Young People in England, Scotland and Wales (JRF Findings 432)*. York: Joseph Rowntree Foundation 2002.

4 Bender D, Lösel F. Bullying at school as a predictor of delinquency, violence and other anti-social behaviour in adulthood. *Criminal Behaviour and Mental Health* 2011;**21**:99-106.

5 Olweus D. Bullying at school: What we know and what can we do. *Malden, MA: Blackwell* 1993.

6 Farrington DP, Ttofi MM. *School-based programs to reduce bullying and victimization*: Campbell Systematic Reviews 2010.

7 Hahn R, Fuqua-Whitley D, Wethington H*, et al.* Effectiveness of universal school-based programs to prevent violent and aggressive behavior: A systematic review. *American Journal of Preventive Medicine* 2007;**33**:S114-S29.

8 Vreeman RC, Carroll AE. A systematic review of school-based interventions to prevent bullying. *Archives of Pediatrics & Adolescent Medicine* 2007;**161**:78-88.

9 Fletcher A, Bonell C, Sorhaindo A. "We don't have no drugs education": the myth of universal drugs education in English secondary schools? *International Journal of Drug Policy* 2010;**21**:452-8.

10 NASUWT. *English Baccalaureate Survey Summary*. Birmingham: NASUWT 2011.

11 PSHE Association. *Comments on the National Curriculum proposals published in February 2013 from the PSHE education Strategic Partners' Group*. PHSEA 2013.

12 The Office for Standards in Education Children's Services and Skills. School inspection handbook: Handbook for inspecting schools in England under section 5 of the Education Act 2005. Manchester: The Government of the United Kingdom 2016.

13 Bier MC, Zwarun L, Warren VF. Getting Universal Primary Tobacco Use Prevention Into Priority Area Schools A Media Literacy Approach. *Health Promotion Practice* 2011;**12**:152S-8S.

14 Pearson M, Chilton R, Wyatt K*, et al.* Implementing health promotion programmes in schools: a realist systematic review of research and experience in the United Kingdom. *Implementation Science* 2015;**10**:1.

15 Jones SM, Brown JL, Lawrence Aber J. Two-year impacts of a universal school-based social-emotional and literacy intervention: An experiment in translational developmental research. *Child Development* 2011;**82**:533-54.

16 Masten AS, Cicchetti D. Developmental cascades. *Development and Psychopathology* 2010;**22**:491-5.

17 Goleman D. *Emotional intelligence*. New York: Bantam 1995.

18 Jones SM, Brown JL, Aber JL. Three Year Cumulative Impacts of the 4Rs Program on Children's Social-Emotional, Behavioral, and Academic Outcomes. *Society for Research on Educational Effectiveness* 2010.

19 Jones SM, Brown JL, Hoglund WL*, et al.* A school-randomized clinical trial of an integrated social–emotional learning and literacy intervention: Impacts after 1 school year. *Journal of Consulting and Clinical Psychology* 2010;**78**:829.

20 The British Heart Foundation. Money to Burn lesson plan. 2014.

For peer review only - http://bmjopen.bmj.com/site/about/guidelines.xhtml

21 Wright G, Ainsworth P. Plastered evaluation: part of It's Not OK! Violence Prevention Education Programme. Liverpool: Ariel Trust 2008.
22 Flay BR, Graumlich S, Segawa E*, et al.* Effects of 2 prevention programs on high-risk behaviors among African American youth: a randomized trial. *Archives of Pediatrics & Adolescent Medicine* 2004;**158**:377-84.
23 Kupersmidt JB, Scull TM, Benson JW. Improving media message interpretation processing skills to promote healthy decision making about substance use: the effects of the middle school media ready curriculum. *Journal of Health Communication* 2012;**17**:546-63.
24 Patton G, Bond L, Carlin JB*, et al.* Promoting social inclusion in schools: group-randomized trial of effects on student health risk behaviour and well-being. *American Journal of Public Health* 2006;**96**:1582-7.
25 Peersman G, Oliver S, Oakley A. EPPI-Center review guidelines: data collection for the EPIC database. London: EPPI-Centre Social Science Research Unit 1997.
26 Higgins JPT, Green S. *Cochrane Handbook for Systematic Reviews of Interventions Version 5.1.0 [updated March 2011]*. Oxford: The Cochrane Collaboration 2011.
27 Sutcliffe K, Thomas J, Stokes G*, et al.* Intervention component analysis (ICA): a pragmatic approach for identifying the critical features of complex interventions. *Systematic Reviews* 2015;**4**:140.
28 Bond L, Patton G, Glover S*, et al.* The Gatehouse Project: can a multilevel school intervention affect emotional wellbeing and health risk behaviours? *Journal of epidemiology and community health* 2004;**58**:997-1003.
29 Espelage DL, Low S, Van Ryzin MJ*, et al.* Clinical trial of Second Step Middle School Program: Impact on bullying, cyberbullying, homophobic teasing, and sexual harassment perpetration. *School Psychology Review* 2015;**44**:464-79.
30 Espelage DL, Low S, Polanin JR*, et al.* Clinical trial of Second Step© middle-school program: Impact on aggression & victimization. *Journal of Applied Developmental Psychology* 2015;**37**:52-63.
31 Espelage DL, Low S, Polanin JR*, et al.* The Impact of a Middle School Program to Reduce Aggression, Victimization, and Sexual Violence. *Journal of Adolescent Health* 2013;**53**:180-6.
32 Couch L. The Bullying Literature Project: An Evaluation of a Class-Wide Bullying Intervention Program. *Education*. California: University of California Riverside 2015.
33 Jenson JM, Dieterich WA. Effects of a skills-based prevention program on bullying and bully victimization among elementary school children. *Prevention Science* 2007;**8**:285-96.
34 Li K-K, Washburn I, DuBois DL*, et al.* Effects of the Positive Action programme on problem behaviours in elementary school students: A matched-pair randomised control trial in Chicago. *Psychology and Health* 2011;**26**:187-204.
35 Frey KS, Hirschstein MK, Snell JL*, et al.* Reducing playground bullying and supporting beliefs: an experimental trial of the steps to respect program. *Developmental Psychology* 2005;**41**:479.
36 Crean HF, Johnson DB. Promoting Alternative Thinking Strategies (PATHS) and elementary school aged children's aggression: Results from a cluster randomized trial. *American Journal of Community Psychology* 2013;**52**:56-72.
37 Reid JB, Eddy JM, Fetrow RA*, et al.* Description and immediate impacts of a preventive intervention for conduct problems. *American Journal of Community Psychology* 1999;**27**:483-518.
38 Langford R, Bonell CP, Jones HE*, et al.* The WHO Health Promoting School framework for improving the health and well-being of students and their academic achievement. *The Cochrane Database of Systematic Reviews* 2014;**4**:CD008958.

For peer review only

39 Wang C, Goldberg TS. Using children's literature to decrease moral disengagement and victimization among elementary school students. *Psychology in the Schools* 2017:No-Specified.
40 Brown EC, Low S, Smith BH*, et al.* Outcomes from a school-randomized controlled trial of steps to respect: A bullying prevention program. *School Psychology Review* 2011;**40**:423.
41 Beets MW, Flay BR, Vuchinich S*, et al.* Use of a social and character development program to prevent substance use, violent behaviors, and sexual activity among elementary-school students in Hawaii. *American Journal of Public Health* 2009;**99**:1438-45.
42 Jenson JM, Brisson D, Bender KA*, et al.* Effects of the Youth Matters prevention program on patterns of bullying and victimization in elementary and middle school. *Social Work Research* 2013:svt030.
43 Lewis KM, Schure MB, Bavarian N*, et al.* Problem behavior and urban, low-income youth: A randomized controlled trial of Positive Action in Chicago. *American Journal of Preventive Medicine* 2013;**44**:622-30.
44 Aber JL, Tubbs C, Torrente C*, et al.* Promoting children's learning and development in conflict-affected countries: Testing change process in the Democratic Republic of the Congo. *Development and Psychopathology* 2017;**29**:53-67.
45 Torrente C, Johnston B, Starkey L*, et al.* Improving the Quality of School Interactions and Student Well-Being: Impacts of One Year of a School-Based Program in the Democratic Republic of the Congo. *Journal on Education in Emergencies* 2015;**1**:48-91.
46 Jenson JM, Dieterich WA, Brisson D*, et al.* Preventing childhood bullying: Findings and lessons from the Denver public schools trial. *Research on Social Work Practice* 2010.

For peer review only

Figures

Figure 1. PRISMA flowchart

For peer review only

Table 1. Characteristics of included studies

**Evaluation, setting and studies**	**Sample characteristics**	**Intervention description**	**Control description**
**Bullying Literature Project**<br><br>California, USA<br><br>Couch 2015[32]	4 classrooms, 95 teacher reports, 90 students (IG); 3 classrooms, 55 teacher reports, 42 students (CG)<br><br>Students enrolled in years 4 and 5, followed up one week post-intervention<br><br>42.8% female, 57.2% male<br><br>9.6% African American, 63.3% Hispanic, 9.0% Caucasian, 3.0% Asian, 4.2% other, 10.2% did not report<br><br>>50% of students received free or reduced-cost lunch	This intervention aims to reduce bullying by introducing themes related to bullying through children's literature. It also provides an opportunity for children to role-model practical skills to address or avoid bullying. The Bullying Literature Project integrates themes related to bullying into the children's literature used within a standard English curriculum. Students then had the opportunity to practice and reinforce skills via writing activities. The intervention was delivered by school psychologists supervised by the PI and lasted over five weeks in one school term. Additionally, the version including moral disengagement discussed the role of moral disengagement in each lesson as well.	Waitlist control
**Bullying Literature Project—Moral Disengagement**<br><br>California, USA<br><br>Wang 2017[39]	2 classrooms, 42 students (IG); 2 classrooms, 42 students (CG)<br><br>Students enrolled in year 4, followed up one week post-intervention<br><br>53.6% female, 46.4% male<br><br>2.4% Asian, 3.6% Caucasian, 94% Hispanic<br><br>SES details not reported		Waitlist control
**Gatehouse**	2 districts, 12 schools, 1335 students (IG);	Through teaching a curriculum (including integration of	Education as

For peer review only

Melbourne, Australia<br><br>Bond 2004[28]	2 districts, 14 schools, 1343 students (CG)<br><br>Students enrolled in Year 9, followed up for three years<br><br>53.2% female, 46.8% male<br><br>87.5% Australian-born<br><br>79.2% from two-parent family; 24.2% speak language other than English at home	cognitive behavioural principles in English classes) and establishing a school-wide adolescent health team, Gatehouse aims to: build a sense of security and trust in students; enhance skills and opportunities for good communication; and build a sense of positive regard through participation in school life. The intervention was delivered by teachers over the course of two school years, supported by the schoolwide adolescent health team and by external consultants who themselves were experiences teachers. Integration was achieved by using English classes to convey cognitive behavioural techniques for self-management, including via a 'critical literacy' approach that uses poetry, literature, song, film and visual materials.	usual
**Learning to Read in a Healing Classroom**<br><br>Democratic Republic of the Congo<br><br>Torrente 2015[45]<br>Aber 2017[44]	20 districts, 33 schools (IG); 19 districts, 30 schools (CG); 3,857 students overall<br><br>Students enrolled in Years 3-5, followed up for one year<br><br>48% female, 52% male	The intervention is delivered over the course of a year and is designed for use in post-conflict reconstruction areas. Teachers are supported to integrate social-emotional learning in literacy lessons, supported by a bank of lesson plans relating to reading and writing. Teachers additionally received substantial professional development, including 'teacher learning circles', and developed strategies to improve the learning environment.	Waitlist control
**Linking the Interests of Families and Teachers (LIFT)**<br><br>Pacific Northwest, USA<br><br>Reid 1999[37]	3 schools, 214 students (IG); 3 schools, 147 students (CG)<br><br>Students enrolled in year 6 and followed up over seven years<br><br>49% female, 51% male<br><br>86% White, 14% ethnic minority	Classroom instruction and discussion on specific social and problem-solving skills followed by skills practice, reinforced during free play using a group cooperation game with review of behaviour and presentation of daily rewards. There is also a parent evening to engage families and opportunities for parents to engage with teachers. The intervention was delivered by teachers and special instructors. Integration was achieved by teaching study skills alongside social-emotional	Education as usual

For peer review only

	12% mother less than high school graduate, 8% father less than high school graduate; 36% mother unemployed, 10% father unemployed; 22% single-parent families; 18% receiving benefits; 20% less than $15,000/year in early 1990s	education content, and was delivered over the course of one school year.	
**Positive Action Chicago**<br><br>Chicago, USA<br><br>Li 2011[34]<br>Lewis 2013[43]	7 schools, ~240 students (IG); 7 schools, ~260 students (CG)<br><br>Students enrolled in year 4 and followed up over six years<br><br>~48% female, ~52% male<br><br>55% African American, 32% Hispanic, 9% White non-Hispanic, 4% Asian, 5% other or mixed<br><br>83% receiving free lunch	Teachers provide lessons covering six units: self-concept; positive actions for mind and body; positive social-emotional actions; managing oneself; being honest with oneself; and continually improving oneself. Content includes 140 lessons per grade per year from years 1 to 13. In addition, an implementation coordinator and school climate team are appointed to support the intervention. The intervention is primarily delivered by teachers and school staff; in both trials, this was supported by extensive professional development and training. Integration was achieved by linking academic learning to social-emotional and health-related learning, e.g. by including content on problem solving and study skills alongside positive actions for mind and body, and by encouraging teachers to reflect Positive Action content in academic lessons.	Education as usual
**Positive Action Hawaii**<br><br>Hawaii, USA<br><br>Beets 2009[41]	10 schools, 976 students (IG); 10 schools, 738 students (CG)<br><br>Students enrolled in years 2 or 3 and followed up over four or five years<br><br>50% female, 50% male<br><br>26.1% Hawaiian, 22.6% mixed, 8.6% White, 1.6% African American, 1.7% American Indian, 4.7% other Pacific Islander, 4.6% Japanese, 20.6% other		Education as usual

For peer review only

	Asian, other 7.8%, unknown 1.6%.<br><br>Control schools had on average 55% free/reduced lunch students, whereas intervention schools had on average 56% free/reduced lunch students		
**Promoting Alternative Thinking Strategies (PATHS)**<br><br>Minnesota and New York State, USA<br><br>Crean 2013[36]	7 schools, 422 students (IG); 7 schools, 357 students (CG)<br><br>Students enrolled in year 4 and followed up over three years<br><br>57% female, 43% male<br><br>51% White, 38% African American, 10% other, 17% Hispanic<br><br>33% from single parent homes; 39% families with income less than $20,000/year, 43% below the federal poverty line; 11% no parent with high school diploma	An intervention to reduce conflict by improving students' social-emotional and thinking skills through a curriculum (including study skills), the establishment of a positive classroom environment and generalised positive social norms throughout the school environment. Lessons are grouped into three units addressing readiness and self-control, feelings and relationships, and interpersonal problem solving. These units cover five domains: 1. Self-control; 2. Emotional understanding; 3. Positive self-esteem; 4. Healthy relationships; and 5. Interpersonal problem-solving skills. The intervention is delivered by teachers supported by consultants, with 131 lessons delivered over three years (two to three times per week, 20 to 30 minutes each). Integration was achieved by linking study skills to social-emotional learning, by supporting teachers to include children's literature in reinforcing concepts, and by providing ideas to link PATHS to English, social studies and history lessons.	Education as usual
**Reading, Writing, Respect and Reconciliation (4Rs)**<br><br>New York City, USA	9 schools, 515 students (IG); 9 schools, 427 students (CG)<br><br>Students enrolled in year 4 followed up for three years (only results up to two years available)	This intervention includes two components: 1. a seven-unit, 21–35 lesson literacy-based curriculum in conflict resolution and social-emotional learning for children in primary school (from year 1 to year 6); and 2. intensive professional development for teachers. The intervention was delivered by teachers after this extensive professional development. Integration was achieved by	Education as usual

For peer review only

Jones 2010[19]<br>Jones 2011[15]	51.2% female, 48.8% male<br><br>41.1% African American, 45.6% Hispanic, 4.7% Caucasian, 8.6% other<br><br>31% low parental education, 15.1% parental unemployment, 53.4% single-parent household, 61.8% living in poverty	using literature as a springboard to help students understand anger and develop skills in listening, cooperation, assertiveness and negotiation.	
**Second Step**<br><br>Illinois and Kansas, USA<br><br>Espelage 2013[31]<br>Espelage 2015a[30]<br>Espelage 2015b[29]	18 schools, 1,940 students (IG); 18 schools, 1,676 students (CG)<br><br>Students enrolled in year 7, followed up yearly over three years<br><br>48.1% female, 51.9% male<br><br>26.4% African American, 24.7% Caucasian, 34.2% Hispanic, 14.7% biracial and all others<br><br>74.1% free or reduced lunch	This intervention includes 15 weeks of classroom lessons taught weekly or every two weeks throughout the school year for three years. Teachers are supported by professional development training to deliver intervention content, which includes bullying, problem-solving, emotional regulation and empathy, alongside videos. Teachers also receive plans to support integration of Second Step content into academic lessons. Modelling, role-play and coaching are included in the intervention. Students receive homework to reinforce skills, and use group and collaborative work to practice skills.	Education as usual, with additional bullying resources
**Steps to Respect I**<br><br>Pacific Northwest, USA<br><br>Frey 2005[35]	3 schools (IG), 3 schools (CG); 1,126 students total<br><br>Students enrolled in years 4 through 7; followed up for one year in endpoint-based analyses<br><br>49.4% female, 50.6% male<br><br>70.0% White, 9% African American, 12.7% Asian, 7.0% Hispanic, 1.3% Native	This is an anti-bullying intervention with both school-wide and classroom components. The School-wide components create new disciplinary policies for bullying and improve monitoring of and intervention in bullying. Classroom curricula positive social norms and improve social–emotional skills for better engagement with bullying. The intervention was delivered by classroom teachers alongside schoolwide bullying policy teams. Biweekly lessons in the Steps to Respect curriculum are supported by 8-to-10 literature-based lessons presented over a 12 to 14 week period. This	Waitlist control

For peer review only

	American<br><br>SES indices not stated	intervention integrates academic and health education by developing literacy skills alongside furthering understanding of the Steps to Respect curricular themes.	
**Steps to Respect II**<br><br>North-Central California, USA<br><br>Brown 2011[40]	17 schools (IG), 16 schools (CG); 2,940 students total<br><br>Students enrolled in years 4 through 6; followed up for one year<br><br>51% female, 49% male (IG); 48% female, 52% male (CG)<br><br>52% White, 7% African American, 6% Asian, 43% Hispanic, 35% other or mixed race (IG); 53% White, 6% African American, 6% Asian, 41% Hispanic, 35% other or mixed race (CG)<br><br>School-level average of 40% on free or reduced-price lunch		Waitlist control
**Youth Matters**<br><br>Denver, USA<br><br>Jenson 2007[33]<br>Jenson 2010[46]<br>Jenson 2013[42]	14 schools, 702 students (IG), 14 schools, 462 students (CG)<br><br>Students enrolled in year 5 and followed up for three years<br><br>50.6% female, 49.4% male<br><br>59.1% Latinx, 14.7% African American, 16.8% American Indian, Asian American, or mixed, 9.3% Caucasian	Youth Matters promotes the development of healthy relationships and social competency and the development of social resistance. Classroom discussions around social issues promote positive social norms. Over four modules with 10 lessons, delivered over two years, students read age-appropriate stories, receive social-emotional learning and practice skills. The intervention was delivered by educational specialists from outside the school. Integration was achieved by using 30-40 page stories in each module intended to support schools in meeting academic standards in academic and health education.	Education as usual

	SES indices not reported		

For peer review only

Table 2. Appraisal of included studies

Intervention name	Random generation of allocation sequence	Concealed allocation	Blinding	Complete outcome data	Reporting not selective	Controlled for confounding	Accounted for clustering	Reduced other forms of bias	Suitable control group
Bullying Literature Project	NC	NS	NS	Yes	NC	NC	NS	NS	NC
Bullying Literature Project—Moral Disengagement	NS	NS	NS	Yes	Yes	Yes	No	Yes	Yes
Learning to Read in a Healing Classroom	Yes	Yes	NS	No	Yes	Yes	Yes	Yes	Yes
Linking the Interests of Families and Teachers (LIFT)	Yes	NS	Yes	Yes	NC	Yes	Yes	Yes	NC
Positive Action Hawaii	NC	NS	NS	Yes	NC	NC	Yes	Yes	Yes
Positive Action Chicago	Yes	NS	NS	Yes	NC	Yes	Yes	Yes	Yes
Promoting Alternative Thinking Strategies (PATHS)	NC	NS	NS	Yes	NC	NC	Yes	Yes	NC
Reading Writing, Respect and Resolution (4Rs)	Yes	NS	NS	Yes	No	Yes	Yes	Yes	Yes
Second Step	Yes	NS	NS	Yes	No	Yes	Yes	Yes	Yes
Steps to Respect I	NC	NS	NS	Yes	NC	NC	Yes	Yes	NC
Steps to Respect II	NC	NS	NS	Yes	NC	NC	Yes	Yes	Yes
Gatehouse	NC	NS	NS	Yes	NC	Yes	Yes	Yes	Yes

For peer review only

Youth Matters	NC	NS	NS	Yes	NC	Yes	Yes	NS	Yes

Legend: NC = not clear; NS = not stated

For peer review only

Table 3. Key themes in the intervention components analysis.

Key theme	Components within theme	Bullying Literature Project	Gatehouse	Learning to Read in a Healing Classroom	LIFT	Positive Action	PATHS	4Rs	Second Step	Steps to Respect	Youth Matters
Approach to integration	Literature: did interventions use literature and language arts as the key vehicle for delivery?<br>Local development: did interventions support teachers to link health education across academic subjects in each school?<br>Linking to developmental concerns: did interventions link academic education and personal health and development?	**Literature:** use of children's books as basis for lessons	**Literature:** English classes used as a key vehicle for delivery of cognitive behavioural content relating to emotional learning and self-regulation<br>**Linking to developmental concerns:** underlying the theory of change was a connection between school health and wellbeing and academic attainment, and the need to create 'healthy' schools	**Literature:** use of reading and language arts lessons to support socio-emotional learning by providing lesson plans	**Linking to developmental concerns:** study skills were presented alongside socio-emotional learning skills, such as empathy and how to play with peers. This content was restricted to the year 6 arm of the intervention	**Local development:** teachers are supported to integrate health education lessons (both social-emotional learning and health and wellbeing, e.g. hygiene) throughout academic learning<br>**Linking to developmental concerns:** key aspect of theory of change is linking academic achievement with physical and mental health and wellbeing, character development	**Linking to developmental concerns:** content on social-emotional learning was presented alongside study skills in later years of the programme<br>**Literature:** English (but also history and social studies classes) was used as a key opportunity to reinforce concepts taught in discrete manualised social-emotional learning lessons	**Literature:** the intervention centres on a literacy-based curriculum relating conflict resolution and social-emotional learning to children's literature	**Local development:** social-emotional learning is integrated into academic lessons alongside a manualised programme of content relating specifically to bullying, problem-solving, emotional regulation content and multimedia resources	**Literature:** the classroom component of this intervention relates to a programme of literature-based lessons designed to convey anti-bullying messages	**Literature:** stories are used to discuss healthy relationships, resistance to bullying and aggressive behaviours, and to practice skills, including via projects relating to literacy lessons
Domains of integration	Classroom: did interventions focus on the classroom?<br>Classroom and whole-school: did interventions include whole-school change components	**Classroom:** focus on classroom teaching only via book-reading and accompanying activities	**Classroom and whole-school domains:** in addition to classroom learning strategies, a school health team supported by eternal consultants	**Classroom and whole-school domains:** in addition to lessons plans to support classroom teaching, pedagogic	**Classroom, whole-school and external domains:** in addition to supporting study skills alongside social-emotional learning,	**Classroom, whole-school and external domains:** in addition to extensively manualised lessons, a school climate team was assembled as part of the	**Classroom, whole-school and external domains:** manualised lessons relating to social-emotional learning and self-regulation are	**Classroom:** teachers receive substantial professional development to implement the intervention using specific materials	**Classroom:** intervention delivered in the classroom context specifically	**Classroom and whole-school domains:** in addition to classroom literacy-based learning, a whole-school	**Classroom and whole-school domains:** lessons delivered in the classroom context, but whole-school events 'showcasing'

For peer review only

	alongside classroom components? Classroom, whole-school and external domains: did interventions also include parent engagement alongside classroom and whole-school components?		sought to identify ways to improve school climate to promote health and wellbeing	circles facilitated school meetings led to exchange of ideas on how to improve school climate	parents received a series of parenting classes and teachers were encouraged to communicate with parents via a phone line recorded message	intervention with a schoolwide 'champion' for intervention implementation. Parents are also involved through homework and 'take-home' assignments, as well as community engagement, though this was not a feature in the Chicago trial	accompanied with school-wide implementation to promote generalised positive norms and parent information	prepared as part of the intervention		policy team developed schoolwide responses to bullying	work part of the intervention activities
Degree of integration	Did interventions include full or partial integration of health education alongside academic education?	**Full integration:** lessons designed to develop literacy skills	**Full integration:** the use of 'critical literacy' to convey social-emotional learning was seamlessly integrated into English classes	**Full integration:** lessons designed to integrate social-emotional learning into enhanced provision of reading and literacy	**Partial integration:** the intervention was set apart from other academic learning	**Partial integration:** discrete lessons relating to Positive Action are presented as part of the intervention	**Partial integration:** manualised intervention lessons presented alongside academic content	**Full integration:** learning is presented alongside literature and reading lessons	**Partial integration:** separate lessons for intervention content are presented alongside integration	**Full integration:** lessons designed to address key literacy goals	**Full integration:** intervention 'led' by literacy and literature content
Timing of integration	Were interventions one year or multiple years in duration?	One year	Multiple years	Multiple years	One year	Multiple years	Multiple years	Multiple years	Multiple years	Multiple years/one year	Multiple years

Table 4. Measures used in included studies and effect estimates

Evaluation	Measure	Notes	Effect estimate
**Violence perpetration**			
4Rs	Aggression	Frequency score on 13 aggressive behaviours assessed by teacher report in last month, including physical aggression and threatening of others	**KS2**<br>End of first year: regression-estimated *b*=0.02, SE=0.05, based on a 1-4 scale<br>End of second year: *d*=-0.21, *p*<0.05
Bullying Literature Project	Physical bullying	Assessed by teacher and student report; mean of frequency scores relating to reports of violence	**KS2**<br>Teacher report: IG: M=1.12, SD=0.47, n=95 vs. CG: 1.19, SD=0.47, n=55; *p*=0.67<br>Student report: 1.20, 0.44, n=90 vs. 1.14, 0.36, n=42; *p*=0.84
Bullying Literature Project—Moral Disengagement	Bullying	Assessed by student report; mean of frequency scores relating to physical and emotional bullying	**KS2**<br>No significant difference from time by treatment interaction: F(1, 80)=0.83, p=.431
LIFT	Change in child physical playground aggression	Measured by observation; includes physical bullying by observed children	**KS2**<br>'Statistically significant' differences: *d*=-0.14 at mean, 1 SD and 2 SD above the pre-intervention mean
PATHS	Aggression	Assessed by teacher and student report; mean of frequency scores relating to verbal and physical aggression	**KS2**<br>Student report: decrease at end of first year *d*=-0.048, 95% CI (-0.189, 0.092); start of second year (-0.064, [-0.205, 0.076]); end of second year (-0.048, [-0.188, 0.093]); but increase end of the third year (0.082, [-0.060, 0.224])<br>Teacher report: increase at end of the first year (0.036, [-0.105, 0.178]), start of second year (0.035, [-0.107, 0.178]) but decrease end of second year (-0.005, [-0.146, 0.136]) and end of third year (-0.199, [-0.338, -0.060])

Positive Action Chicago	Bullying	Student report: count of bullying behaviours relating to verbal or physical aggression behaviours in the past two weeks<br>Parent report: count of observed verbal or physical aggression behaviours in past 30 days	**KS2**<br>Student report IRR [incidence rate ratio] =0.59, 95% CI (0.37, 0.92)<br>**KS3**<br>Student report: *d*=-0.39<br>Parent report: *d*=-0.31
	Violence-related behaviours	Count of lifetime behaviours: carried a knife, threatened to cut or stab someone, cut of stabbed someone on purpose, been asked to join a gang, hung out with gang members, been a member of a gang	**KS2**<br>IRR=0.63, 95% CI [0.45, 0.88]<br>**KS3**<br>IRR=0.38, 95% CI [0.18, 0.81], or *d*=-0.54
Positive Action Hawaii	Count of violent behaviours	Teacher, student report	**KS2**<br>Teacher report: IRR=0.54, 90% CI [0.30, 0.77]<br>Student report: IRR=0.42, 90% CI [0.24, 0.73]
	Cut or stabbed others	Student report, lifetime prevalence	**KS2**<br>OR=0.29, 90% CI [0.16, 0.52]
	Shot another person	Student report, lifetime prevalence	**KS2**<br>OR=0.24, 90% CI [0.14, 0.40]
	Physically hurts others	Teacher report	**KS2**<br>OR=0.61, 90% CI [0.38, 0.97]
	Gets into a lot of fights	Teacher report	**KS2**<br>OR=0.63, 90% CI [0.47, 0.84]
Second Step	Physical aggression perpetration	Student report, endorse any fighting behaviours in last 30 days	**KS3**<br>End of first year: OR=0.70, $p<0.05$<br>End of second year: OR=0.80, 95% CI [0.59, 1.08]<br>End of third year: β=0.005, SE=0.012
	Sexual harassment and violence perpetration	Student report, endorse any verbal sexual violence or groping behaviours or forced sexual contact	**KS3**<br>End of first year: OR=1.04, $p>0.05$<br>End of second year: Illinois schools 0.72 [0.54, 0.95], Kansas schools 0.99 [0.71, 1.48]
Steps to Respect I	Bullying	Playground observation of students	**KS2**<br>Decrease in intervention group: $F(91.3)=5.02$, $p<0.01$
	Direct	Mean of student reported frequency scores of direct	Decrease not significant in intervention group

For peer review only

	aggression	bullying	compared to control: *F*(68.7)=2.05, *p*>0.05
Steps to Respect II	Bullying perpetration	Measured by student report; proportion of students with at least one bullying behaviour	**KS2**<br>Intervention group not significantly lower than control group: *t*(29)=-1.06
	Physical bullying perpetration	Measured by teacher report; proportion of students with at least one physical bullying behaviour	**KS2**<br>Significantly less in intervention group: OR=0.61, *t*(29)=-3.12, *p*<0.01
Youth Matters	Bullying	At least two or three times a month on at least one bullying behaviour	**KS2**<br>OR=0.85, 95% CI [0.29, 1.47], *p*=0.585
	Bully, victim, or bully-victim	Classification of students based on questionnaire responses into one of three categories	Bully or bully-victim<br>**KS2**<br>end of first year IG: 21%, n=356 vs CG: 22%, n=392; end of second year 19%, n=244 vs 23%, n=293<br>**KS3**<br>both groups 16%; IG n=283, CG n=289
**Violence victimisation**			
Bullying Literature Project	Physical bullying	Assessed by teacher and student report; mean of frequency scores relating to reports of violence	**KS2**<br>Teacher report: IG: M=1.04, SD=0.23, n=95 vs. CG: 1.04, SD=0.21, n=55; *p*=0.39<br>Student report: (1.35, 0.54, n=90 vs. 1.43, 0.66, n=42; *p*=0.57
Bullying Literature Project—Moral Disengagement	Bullying victimisation	Assessed by student report; mean of frequency scores relating to physical and emotional bullying	**KS2**<br>Student report: IG: M=1.76, SD=0.81 to M=1.60, SD=0.66, n=42 vs. CG: M=1.23, SD=0.38 to M=1.38, SD=0.53, n=42; F(1, 80)=7.42, *p*=0.047
Gatehouse	Bullying victimisation	Assessed by student report; any of being teased, having rumours spread about them, deliberate exclusion or experience of threats or violence	**KS4**<br>End of first year OR=1.03, 95% CI [0.86, 1.26]<br>End of second year OR=1.03, [0.78, 1.34]<br>End of third year OR=0.88, [0.68, 1.13]
Learning to Read in a Healing	Victimisation	Assessed by student report; average of frequency scores of peer verbal and physical bullying	**KS2**<br>weighted *d*=-0.01, SE=0.06

For peer review only

Classroom			
PATHS	Victimisation	Assessed by student report; sum of frequency scores of victimisation in last two weeks	**KS2**<br>Increase at the end of the first intervention year (*d*=0.044, 95% CI [-0.098, 0.185]); the start (0.074, [-0.067, 0.216]) and end (0.092, [-0.050, 0.234]) of the second year; and the end of the third year (0.089, [-0.053, 0.231])
Second Step	Peer victimisation	Student report, endorse any physical or verbal victimisation in last 30 days	**KS3**<br>End of first year OR=1.01, p>0.05<br>End of second year OR=0.94, 95% CI [0.75, 1.18]
	Sexual harassment and violence victimisation	Student report, endorse any victimisation by verbal sexual violence or groping behaviours or forced sexual contact	**KS3**<br>End of first year OR=1.01, *p*>0.05<br>End of second year OR=0.91, [0.72, 1.15]
Steps to Respect I	Target of bullying	Playground observation of students	**KS2**<br>IG: M=0.9, SD=0.82 vs. CG: M=1.01, SD=0.83; *F*(72.4)=3.74, *p*<0.10
	Victimisation	Assessed by student report; mean of frequency scores for physical and verbal victimisation items	**KS2**<br>IG: M=0.80, SD=1.51 vs CG: M=0.86, SD=1.44; *F*<1
Steps to Respect II	Victimisation	Assessed by student report; mean of frequency scores for physical and verbal victimisation items	**KS2**<br>IG: M=2.11, SD=1.03 vs. CG: M=2.18, SD=1.06; *t*(29)=-1.15
Youth Matters	Victimisation	Assessed by student report; mean of frequency scores for physical and verbal victimisation items, and also at least two or three times a month victimisation at least one bullying behaviour	**KS2**<br>difference=-0.171, SE=0.083, *p*=0.049; OR=0.61, *p*=0.098<br>**KS3**<br>regression-estimated difference=-0.123, SE=0.068, *p*=0.08
	Bully, victim, or bully-victim	Classification of students based on questionnaire responses into one of three categories	Victim or bully-victim<br>**KS2**<br>No difference between groups

For peer review only

			**KS3**<br>IG: 36%, n=283 vs CG: 45%, n=289

For peer review only

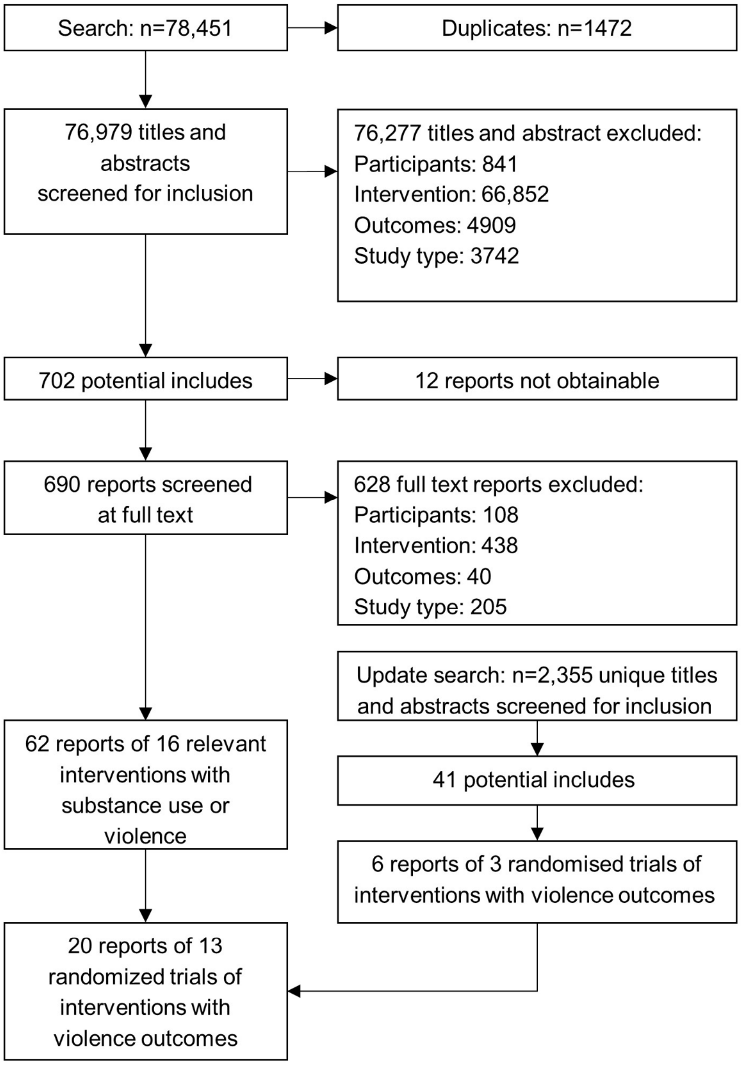

PRISMA flowchart

154x190mm (300 x 300 DPI)

Additional search and synthesis methods

We searched 19 electronic databases. The original list of databases was amended after consultation with our information scientist, as informed by initial searches.

- ASSIA via Proquest
- Australian Educational Index via Proquest
- BiblioMap (Database of health promotion research) via EPPI-Centre
- British Educational Index via EBSCOhost
- Cochrane Central Register of Controlled Trials via the Cochrane Library
- Cochrane Database of Systematic Reviews via the Cochrane Library
- Database of Abstracts of Reviews of Effects via the Cochrane Library
- Database of Promoting Health Effectiveness Reviews (DoPHER) via EPPI-Centre
- Dissertation Abstracts (UK theses, all dates; global theses 2010-2015) via Proquest
- Econlit via EBSCO
- Educational Research Index Citations via EBSCO
- Health Technology Assessment Database via the Cochrane Library
- International Bibliography of the Social Sciences via Proquest
- MEDLINE via OVID
- NHS Economic Evaluation Database
- PsycINFO via OVIDa
- Social Policy and Practice Including Child Data & Social Care Online via OVID
- Social Science Citation Index via Web of Knowledge
- Trials Register of Promoting Health Interventions via EPPI-Centre

We also searched the following 32 websites:

- Cambridge Journals
- Centers for Disease Control and Prevention: Smoking & Tobacco Use
- Child and Adolescent Research Unit
- Childhoods Today
- Children in Scotland
- Children in Wales
- Community Research and Development Information Service
- Database of Educational Research (EPPI-Centre)
- Drug and Alcohol Findings Effectiveness Bank
- Google
- Google Scholar
- Government of Wales
- Government of Scotland
- Joseph Rowntree Foundation
- National Criminal Justice Reference Service
- National Society of the Prevention of Cruelty to Children
- National Youth Agency
- Northern Ireland Executive
- OpenGrey
- Personal Social Services Research Unit
- Project Cork
- UCL-IOE Digital Education Resource Archive
- UK Clinical Research Net Study Portfolio
- University of Illinois at Urbana Champaign
- US Centre for Substance Abuse Prevention
- Social Issues Research Centre
- The Campbell Library
- The Children's Society
- The Open Library
- The Schools and Students' Health Education Unit Archive
- WHO International Clinical Trials Registry Platform
- Young Minds: Child & Adolescent Mental Health

For peer review only

PsycINFO search string

1. ((substance? or drug? or drinking or alcohol* or solvent?) adj1 ("use" or abus* or misuse*)).ti,ab.
2. ((substance? or drug? or drinking or alcohol* or solvent?) adj1 (usage or intake or using or taking or behavio* or user?)).ti,ab.
3. (drinking adj1 (alcohol* or behavio*)).ti,ab.
4. Alcohol.ti,ab.
5. (smoke or smoking or tobacco or cigarette? or smoker? or cannabis or marijuana).ti,ab.
6. (aggression or aggressive or bully* or delinquen* or "conduct problem*" or "conduct disorder?" or "antisocial" or "anti social" or violence or violent or (volatile adj behavio*) or victimi* or hostile or hostility or perpetrat*).ti,ab.
7. (Externalising or externalizing).ti,ab.
8. emotion*.ti,ab.
9. PSHE.ti,ab.
10. ("Health literacy" or "health education" or "health promotion" or "preventive health" or "primary prevention" or "health information" or "promoting health" or "health promoting" or "health promotion" or "health maintenance").ti,ab.
11. "Public health".ti,ab.
12. ("wellbeing" or "well being").ti,ab.
13. "mental health".ti,ab.
14. 1 or 2 or 3 or 4 or 5 or 6 or 7 or 8 or 9 or 10 or 11 or 12 or 13
15. ((curric* or lesson? or classes or classroom? or subject? or intervention? or program* or education or initiative? or learn or learning or teach or teaching or outcome* or attainment or achievement or assessment or effect* or impact* or score? or scoring* or skill? or knowledge or competen* or performance) adj3 (Academic or academically or Scholastic or scholar* or Mainstream or "main stream")).ti,ab.
16. ((curric* or lesson? or classes or classroom? or subject? or learn or learning or teach or teaching or attainment or achievement or assessment or score? or scoring* or skill? or knowledge or competen* or performance) adj3 School?).ti,ab.
17. ((intervention? or program* or initiative? or effect* or impact* or education) adj1 School?).ti,ab.
18. (class adj1 (Academic or academically or Scholastic or scholar* or School? or Mainstream or "main stream")).ti,ab.
19. ((curric* or lesson? or classes or classroom? or subject? or education or learn or learning or teach or teaching or attainment or achievement or score? or scoring* or skill? or knowledge or competen*) adj3 (study or core or generic)).ti,ab.
20. (class adj1 (study or core or generic)).ti,ab.
21. ((curric* or lesson? or classes or classroom? or subject? or attainment or achievement or assessment or score? or scoring* or competenc* or performance) adj3 ((Education not ("patient education" or "continuing education")) or educational)).ti,ab.
22. (class adj1 ((Education not ("patient education" or "continuing education")) or educational)).ti,ab.
23. (outcome* adj1 (education or educational)).ti,ab.
24. ((curric* or lesson? or classroom? or classes or subject? or intervention? or program* or initiative? or education or teach* or outcome* or attainment or achievement or assessment or effect* or impact* or score? or scoring* or skill? or knowledge or competen* or performance) adj3 (learn or learning)).ti,ab.
25. (class adj1 (learn or learning)).ti,ab.
26. ((curric* or lesson? or classes or classroom or class or subject? or education or teach* or learning or teach or teaching or learn or attainment or achievement or assessment or score? or scoring* or skill? or knowledge or competen* or performance) adj3 (art or arts or math* or science? or humanities or chemistry or physics or language* or geography or (history not ("medical history" or "health history" or "familial history" or "family history")) or numeracy or (literacy not "health literacy") or grammar or grammer or reading or writing)).ti,ab.
27. (((curric* or lesson? or classroom or classes or subject? or skill?) adj3 literature) or "literature class").ti,ab.
28. ("Education reform" or "Instructional support" or "School reform" or "Classroom organi*" or (Commit* adj3 (school or education or learning)) or (Engag* adj3 (school or education or learning)) or "Character development" or "Whole school" or "School level" or "School wide" or schoolwide).ti,ab.
29. ((Comprehensive adj3 school) and (intervention? or program* or initiative? or outcome* or effect* or impact*)).ti,ab.
30. ((Integrat* or Combin* or Infuse or infused or infusion or sustainable) adj3 (curric* or lesson? or classes or classroom or syllabus or subject? or education or learn or learning or teach or teaching)).ti,ab.
31. (((Integrat* or Combin* or Infuse or infused or infusion or sustainable) adj3 (intervention* or program* or initiative*)) and school?).ti,ab.
32. ((school or education or core or generic or teaching or learning) adj3 syllabus).ti,ab.

For peer review only

33. 15 or 16 or 17 or 18 or 19 or 20 or 21 or 22 or 23 or 24 or 25 or 26 or 27 or 28 or 29 or 30 or 32
34. (child* or schoolchild* or youth* or "young people*" or "young person" or teen* or adolescen* or juvenile* or preadolescen* or boy? or girl?).ti,ab.
35. (curric* or lesson? or classes or classroom? or subject? or school? or syllabus or "junior high" or "senior high" or "junior education" or "elementary education" or "primary education").ti,ab.
36. 34 and 35
37. ("secondary school?" or "primary school?" or "comprehensive school?" or "school education" or "high school?" or "grammar school?" or "private school?" or "public school?" or "mainstream school*" or "compulsory education" or "statutory education" or "middle school?" or "junior school?" or "senior school?" or "primary education" or "secondary education" or "elementary school?" or "elementary education" or "mainstream education" or "compulsory school*" or "statutory school*" or "sixth form college?" or "post-16 education" or "junior high" or "senior high" or "reception class" or "post primary").ti,ab.
38. ((school? or junior? or elementary or senior? or primary or "sixth form" or grade) adj10 student?).ti,ab.
39. pupil?.ti,ab.
40. 36 or 37 or 38
41. (University or universities or freshmen or sophomore? or "higher education" or "tertiary education" or ((registrar* or workplace? or clinical or medical or nursing or nurse? or doctor? or continuing or adult? or patient?) adj1 (education or educating or profession* or student?)) or "professional education").ti.
42. 40 not 41
43. 14 and 33 and 42
44. "Elementary School Students"/ or "Intermediate School Students"/ or "Primary School Students"/ or "Middle School Students"/ or "High School Students"/ or "Junior High School Students"/ or "Kindergarten Students"/ or "High School Education"/ or "Middle School Education"/ or "Secondary Education"/ or "Junior High Schools"/ or "High Schools"/ or "Schools"/ or "Elementary Schools"/ or "Middle Schools"/
45. "Drug Abuse Prevention"/ or "Health Education"/ or "Drug Education"/ or "Health Promotion"/ or "Public Health"/ or "Health Promotion"/ or "Preventive Medicine"/ or Health behaviour/ or Harm reduction/ or Health literacy/ or exp Health screening/ or Primary Mental health prevention/ or Prevention/ or Public health/ or Lifestyle changes/ or Lifestyle/ or Health literacy/
46. "Tobacco Smoking"/ or "Smoking Cessation"/ or "Marijuana Usage"/ or "Drinking Behavior"/ or "Social Drinking"/ or "Binge Drinking"/ or "Underage Drinking"/ or "Alcohol Abuse"/ or "Alcohol Drinking Patterns"/ or "Alcohol Intoxication"/ or "Alcoholism"/ or "Heroin Addiction"/ or "Drug Addiction"/ or "Drug Dependency"/ or "Drug Usage"/ or "Inhalant Abuse"/ or "Drug Abuse"/ or "Glue Sniffing"/ or "Predelinquent Youth"/ or "Cyberbullying"/ or "School Violence"/ or "Teasing"/ or "Juvenile Delinquency"/ or "Physical Abuse"/ or "Verbal Abuse"/ or "Violence"/ or "Harassment"/ or "Antisocial Behavior"/ or "Bullying"/ or "Perpetrators"/ or "Threat"/ or "Victimization"/ or "Relational Aggression"/ or "Aggressive Behavior"/ or "Behavior Problems"/ or "Behavior Disorders"/ or "Conduct Disorder"/ or "Drug Education"/ or "Drug Abuse Prevention"/ or "Harm Reduction"/
47. emotions/ or emotional development/
48. emotional adjustment/ or emotional disturbances/ or emotional control/
49. mental health/ or primary mental health prevention/ or well being/
50. "Curriculum"/ or "Curriculum Based Assessment"/ or "Curriculum Development"/ or "School Learning"/ or "Classroom Environment"/ or "Academic Environment"/ or "Teacher Effectiveness"/ or "Teacher Effectiveness Evaluation"/ or "Educational Program Evaluation"/ or "Course Evaluation"/ or "learning environment"/
51. 14 or 45 or 46 or 47 or 48 or 49
52. 33 or 50
53. 42 or 44
54. 51 and 52 and 53

For peer review only

PRISMA 2009 Checklist

Section/topic	#	Checklist item	Reported on page #
**TITLE**			
Title	1	Identify the report as a systematic review, meta-analysis, or both.	1
**ABSTRACT**			
Structured summary	2	Provide a structured summary including, as applicable: background; objectives; data sources; study eligibility criteria, participants, and interventions; study appraisal and synthesis methods; results; limitations; conclusions and implications of key findings; systematic review registration number.	2
**INTRODUCTION**			
Rationale	3	Describe the rationale for the review in the context of what is already known.	3-4
Objectives	4	Provide an explicit statement of questions being addressed with reference to participants, interventions, comparisons, outcomes, and study design (PICOS).	4
**METHODS**			
Protocol and registration	5	Indicate if a review protocol exists, if and where it can be accessed (e.g., Web address), and, if available, provide registration information including registration number.	6
Eligibility criteria	6	Specify study characteristics (e.g., PICOS, length of follow-up) and report characteristics (e.g., years considered, language, publication status) used as criteria for eligibility, giving rationale.	6
Information sources	7	Describe all information sources (e.g., databases with dates of coverage, contact with study authors to identify additional studies) in the search and date last searched.	Online File 1
Search	8	Present full electronic search strategy for at least one database, including any limits used, such that it could be repeated.	Online File 1
Study selection	9	State the process for selecting studies (i.e., screening, eligibility, included in systematic review, and, if applicable, included in the meta-analysis).	7
Data collection process	10	Describe method of data extraction from reports (e.g., piloted forms, independently, in duplicate) and any processes for obtaining and confirming data from investigators.	7
Data items	11	List and define all variables for which data were sought (e.g., PICOS, funding sources) and any assumptions and simplifications made.	Online File 1
Risk of bias in individual studies	12	Describe methods used for assessing risk of bias of individual studies (including specification of whether this was done at the study or outcome level), and how this information is to be used in any data synthesis.	7
Summary measures	13	State the principal summary measures (e.g., risk ratio, difference in means).	N/A
Synthesis of results	14	Describe the methods of handling data and combining results of studies, if done, including measures of consistency (e.g., I^2) for each meta-analysis.	8

For peer review only

For peer review only - http://bmjopen.bmj.com/site/about/guidelines.xhtml

PRISMA 2009 Checklist

Section/topic	#	Checklist item	Reported on page #
Risk of bias across studies	15	Specify any assessment of risk of bias that may affect the cumulative evidence (e.g., publication bias, selective reporting within studies).	8
Additional analyses	16	Describe methods of additional analyses (e.g., sensitivity or subgroup analyses, meta-regression), if done, indicating which were pre-specified.	8
**RESULTS**			
Study selection	17	Give numbers of studies screened, assessed for eligibility, and included in the review, with reasons for exclusions at each stage, ideally with a flow diagram.	9, Figure 1
Study characteristics	18	For each study, present characteristics for which data were extracted (e.g., study size, PICOS, follow-up period) and provide the citations.	8-10, Table 1-3
Risk of bias within studies	19	Present data on risk of bias of each study and, if available, any outcome level assessment (see item 12).	Table 2
Results of individual studies	20	For all outcomes considered (benefits or harms), present, for each study: (a) simple summary data for each intervention group (b) effect estimates and confidence intervals, ideally with a forest plot.	11-16
Synthesis of results	21	Present results of each meta-analysis done, including confidence intervals and measures of consistency.	N/A
Risk of bias across studies	22	Present results of any assessment of risk of bias across studies (see Item 15).	N/A
Additional analysis	23	Give results of additional analyses, if done (e.g., sensitivity or subgroup analyses, meta-regression [see Item 16]).	N/A
**DISCUSSION**			
Summary of evidence	24	Summarize the main findings including the strength of evidence for each main outcome; consider their relevance to key groups (e.g., healthcare providers, users, and policy makers).	16
Limitations	25	Discuss limitations at study and outcome level (e.g., risk of bias), and at review-level (e.g., incomplete retrieval of identified research, reporting bias).	17
Conclusions	26	Provide a general interpretation of the results in the context of other evidence, and implications for future research.	17-18
**FUNDING**			
Funding	27	Describe sources of funding for the systematic review and other support (e.g., supply of data); role of funders for the systematic review.	18

From: Moher D, Liberati A, Tetzlaff J, Altman DG, The PRISMA Group (2009). Preferred Reporting Items for Systematic Reviews and Meta-Analyses: The PRISMA Statement. PLoS Med 6(7): e1000097. doi:10.1371/journal.pmed1000097

For more information, visit: **www.prisma-statement.org**.

For peer review only - http://bmjopen.bmj.com/site/about/guidelines.xhtml

BMJ Open

Integration of academic and health education for the prevention of physical aggression and violence in young people: systematic review, narrative synthesis and intervention components analysis

Journal:	*BMJ Open*
Manuscript ID	bmjopen-2017-020793.R2
Article Type:	Research
Date Submitted by the Author:	22-Jun-2018
Complete List of Authors:	Melendez-Torres, G.J.; Cardiff University, DECIPHer, School of Social Sciences<br>Tancred, Tara; London School of Hygiene and Tropical Medicine, Department of Social and Environmental Health Research<br>Fletcher, Adam; Cardiff University<br>Campbell, Rona; University of Bristol, DEICPHer, Bristol Medical School<br>Thomas, James; University College London Social Science Research Unit<br>Bonell, Christopher; London School of Hygiene and Tropical Medicine, Department of Social and Environmental Health Research
**Primary Subject Heading**:	Public health
Secondary Subject Heading:	Paediatrics
Keywords:	systematic review, violence, Community child health < PAEDIATRICS

SCHOLARONE™
Manuscripts

Integration of academic and health education for the prevention of physical aggression and violence in young people: systematic review, narrative synthesis and intervention components analysis

Dr G.J. Melendez-Torres [1]*

Dr Tara Tancred [2]

Prof Adam Fletcher [3]

Prof Rona Campbell [4]

Prof James Thomas [5]

Prof Christopher Bonell [2]

[1] DECIPHer, School of Social Sciences, Cardiff University, Cardiff, UK

[2] Department of Social and Environmental Health Research, London School of Hygiene and Tropical Medicine, London, UK

[3] Cardiff University, Cardiff, UK

[4] DECIPHer, Bristol Medical School, University of Bristol, Bristol, UK

[5] EPPI-Centre, UCL Institute of Education, University College London, London, UK

*Corresponding author
G.J. Melendez-Torres
DECIPHer
School of Social Sciences
Cardiff University
1-3 Museum Place
Cardiff CF10 3BD
UNITED KINGDOM
melendez-torresg@cardiff.ac.uk
+44 (0) 29208 79106

Word count: 4,023

For peer review only

Abstract

Objectives. To systematically review evidence on the effectiveness of interventions including integration of academic and health education for reducing physical aggression and violence, and describe the content of these interventions.

Data sources. Between November and December 2015, we searched 19 databases and 32 websites and consulted key experts in the field. We updated our search in February 2018.

Eligibility criteria. We included randomised trials of school-based interventions integrating academic and health education in students aged 4-18 and not targeted at health-related subpopulations (e.g. learning or developmental difficulties). We included evaluations reporting a measure of interpersonal violence or aggression.

Data extraction and analysis. Data were extracted independently in duplicate, interventions were analysed to understand similarities and differences, and outcomes were narratively synthesised by key stage (KS).

Results. We included 10 evaluations of eight interventions were reported in 14 papers. Interventions included either full or partial integration, incorporated a variety of domains beyond the classroom, and used literature, local development or linking of study skills and health promoting skills. Evidence was concentrated in KS2, with few evaluations in KS3 or KS4, and evaluations had few consistent effects; evaluations in KS3 and KS4 did not suggest effectiveness.

Discussion. Integration of academic and health education may be a promising approach, but more evidence is needed. Future research should consider the 'lifecourse' aspects of these interventions; that is, do they have a longitudinal effect? Evaluations did not shed light on the value of different approaches to integration.

For peer review only

Strengths and limitations of this study

- We used an exhaustive search including 19 databases and 32 websites.
- We used an innovative method to describe key components in this class of interventions.
- However, it was challenging to identify studies for inclusion.
- Meta-analysis was not possible because of the diversity of outcomes and raters.

For peer review only

INTRODUCTION

Violence among young people is a public-health priority due to its prevalence and harms to young people and wider society.[1, 2] One UK study found that 10% of young people aged 11–12 reported carrying a weapon and 8% admitted attacking someone with intent to hurt them seriously.[3] By age 15–16, 24% of students reported they have carried a weapon and 19% reported attacking someone with the intention to hurt them seriously.[3] Early aggression and anti-social behaviour are strongly linked to adult violent behaviour.[4, 5]

School-based health education can be effective in reducing violence.[6-8] However, school-based health education is increasingly marginal in many high-income countries, partly because of schools increasing focus on attainment-based performance metrics. In England specifically, health education is not a statutory subject,[9-11] and school inspectors have a limited focus on how schools promote student health.[12]

One way to avoid such marginalisation is to integrate health education into academic lessons. For example, health-related content can be seamlessly integrated into existing academic lessons or discrete additional health education lessons can also include academic learning elements. This strategy may bring other benefits because: larger 'doses' may be delivered; students may be less resistant to health messages weaved into other subjects; and lessons in different subjects may reinforce each other.[13, 14] Conversely, those teaching academic subjects may be uninterested or unqualified to teach health topics. Though theories of change in this class of interventions are diffuse, one important way in which they could be effective is by promoting developmental cascades involving the interplay of cognitive and non-cognitive skills.[15, 16] Interventions integrating academic and health education could address violence by developing: social and emotional skills such as self-awareness, self-regulation, motivation, empathy and communication;[17] healthier social support or norms

For peer review only

among students[15, 18, 19]; knowledge of the costs [20] and consequences [21] of substance use; media literacy skills to critique harmful media messages; and modifying students' social norms about antisocial behaviours.[13, 20, 22-24] Our work synthesising the theories of change underlying these interventions (Tancred et al., in press) identified that interventions aimed to integrate and thus erode boundaries between academic and health education, between students and teachers (so that relationships were improved and teachers might function more effectively as behavioural role models), and between classrooms and schools and schools and families (so that violence prevention messages communicated in classrooms might be reinforced by messaging in other settings).

Despite policy interest in these interventions, they have not previously been the subject of a specific systematic review. Previous systematic reviews have focused on socio-emotional learning interventions or school-based interventions generally,[6-8] without considering interventions that specifically integrate with academic lessons as defined above. Our focus on violence is informed by preliminary consultation, scoping work and logic model development suggesting that violence is an outcome especially amenable to these interventions. In the present review, we examined the characteristics of interventions that integrate academic and health education to prevent violence, and synthesised evidence for their effectiveness. That is, our research questions were: what are the overarching features relevant to integration of interventions that integrate academic and health education, and are these interventions effective at different key stages in reducing physical aggression and violence?

METHODS

This review was part of a larger evidence synthesis project on theories of change, process evaluations and outcome evaluations of integration of academic and health education for substance use and violence. We registered the protocol for this review on PROSPERO

For peer review only

(CRD42015026464, https://www.crd.york.ac.uk/prospero/), and it is enclosed as Online File 1.

Inclusion and exclusion

Studies were included regardless of publication date or language. We included randomised controlled trials of interventions integrating academic and health education, the former defined as specific academic subjects or general study skills. We defined as 'health education' education seeking to improve the health and wellbeing of students (including social and emotional learning and other forms of violence prevention). We included school-based interventions that seamlessly incorporated health education into existing academic lessons and interventions that provided discrete health education lessons with additional academic components. Interventions could be delivered by teachers or other school staff such as teaching assistants, but may also have been delivered by external providers, for example from the health, voluntary or youth service sectors. We did not include interventions solely addressing social conduct in the classroom; relationships with peers or staff; attitudes to education, school or teachers; or aspirations and life goals. Our definition also excluded interventions which: were delivered in mainstream subject lessons but did not aim to integrate health and academic education; trained teachers in classroom management without student curriculum components; or were delivered exclusively outside of classrooms, as these did not seek to integrate academic and health education. Interventions focusing on targeted health-related sub-populations (e.g. children with cognitive disabilities) were excluded as we were interested in universal interventions.

For this review, we focus on violence outcomes, defined as the perpetration or victimisation of physical violence including convictions for violent crime. While we preferred direct measures of physically violent and physically aggressive behaviours, we included outcomes that were a composite of physical and non-physical (e.g. verbal or

For peer review only

emotional) interpersonal violence, but excluded composite measures that also included items not focused on interpersonal violence, such as damage to property.

Search strategy

In our original search, undertaken between November and December 2015, we searched 19 databases and 32 websites, and contacted subject experts (see Online File 2 for full details). We subsequently updated our search in February 2018 using PsycINFO and CENTRAL, as all of our original study hits were recovered from these databases.

Study selection

Pairs of researchers double-screened titles and abstracts in sets of 50 references until 90% agreement was reached, with disagreements discussed at every stage. Subsequently, single reviewers screened each reference. We located the full texts of remaining references and undertook similar pairwise calibration with disagreements discussed, followed by single screening. Reports were translated into English where necessary. Using an existing tool[25] we extracted data independently in duplicate from included studies and assessed trials for risk of bias using a modified version of the Cochrane assessment tool.[26] Authors were contacted where study data were missing.

Synthesis methods

We undertook an intervention components analysis.[27] This was undertaken inductively by one researcher and audited by two other researchers, and used intervention descriptions to draw out similarities and differences in intervention design using an iterative method. Intervention descriptions were read and re-read and then coded manually. The goal of this analysis was to use a set of descriptors to characterise aspects of the integration of academic and health education in the intervention. Intervention descriptions were rarely detailed enough to permit 'deep' engagement with the specific content of the interventions provided in included evaluations. The intervention components analysis identified

For peer review only

overarching domains that accounted for similarities and differences between interventions in their integration of academic and health education, and developed within each domain a set of overlapping categories that described these similarities and differences between interventions within each domain. Finally, we synthesised outcomes narratively due to the heterogeneity in included outcome measurement. We categorised the timing of intervention effect by period of schooling, defined in terms of English schools' key-stage (KS) system. KS1 includes school years 1–2 (age 5–7 years); KS2 includes years 3–6 (age 7–11 years); KS3 includes years 7–9 (age 11–14 years); KS4 includes years 10–11 (age 14–16 years); and KS5 includes years 12–13 (age 16–18 years).

We could not formally assess publication bias because heterogeneity in outcome measurement precluded meta-analysis.

Patient and public involvement

Because this review focused on public health interventions that were generally preventive in nature, patients were not involved *per se*. However, stakeholders were extensively consulted in the development of research questions and in assessing the implications of the findings. In addition, findings were disseminated via stakeholder events, and a series of one-to-one consultations took place to ensure the relevance and salience of study findings.

RESULTS

In our original search, we found and screened 76,979 references, of which we retained 702 for full-text screening and were able to assess 690. Of 62 relevant reports included in the overall project, 10 evaluations of eight interventions were reported in 14 papers that considered violence and are reported in this review. Our update search yielded 2,355 references, of which we retained 41 for full-text screening and included six papers reporting three evaluations (Figure 1). This yielded a total of 13 evaluations reported in 20 papers.

For peer review only

Included studies and their quality

All trials randomised schools except the Bullying Literature Project, which randomised classrooms (Table 1). All evaluations were conducted in the USA, except for Gatehouse,[28] which was an Australian study. All control arms consisted of education-as-usual or waitlist controls, though Second Step[29-31] offered a brief anti-bullying intervention with low takeup.

Interventions were diverse and are summarised below in the intervention components analysis. Only two interventions (Bullying Literature Project,[32] Youth Matters[33]) were wholly delivered by external staff. Several (Gatehouse,[28] Positive Action,[34] Steps to Respect[35]) linked classroom-based delivery to school-level work to support and reinforce implementation. PATHS[36] and 4Rs[19] also emphasised teachers' professional development.

Evaluation quality varied (Table 2). Appraisal was hampered by poor reporting of some aspects of trial methods. Only four studies reported evidence of low risk of bias for random generation of allocation sequence; the remainder were unclear. Only one study reported information on concealed allocation. In LIFT,[37] outcome assessors were blinded, resulting in low risk of bias in this domain, but all other interventions were of unclear risk of bias. All interventions included reasonably complete outcome data, and in only one evaluation did unit of analysis issues pose a risk of bias. In some studies such as Steps to Respect, follow-up was shorter than intervention length. Evaluations also differed in size, ranging in size from seven classrooms to 63 schools.

Intervention components analysis

This identified four themes describing included interventions: approach to integration, position of integration, degree of integration and point of integration. Included interventions are described in Table 1, and the components analysis is summarised in Table 3.

For peer review only

Approach to integration

Interventions approached the rationale for and strategy of integration in different and overlapping ways. These overlapped across interventions, but were not mutually exclusive, and described the types of academic foci that interventions used to integrate academic and health education. Several (4Rs, Bullying Literature Project, Steps to Respect, Youth Matters) focused on *literature* as a focus for integration, using children's books as a prompt for social-emotional learning. These interventions targeted language arts or literacy lessons as an opportunity to provoke discussion, role-play and modelling of positive strategies to avoid violence. Gatehouse explicitly used a 'critical literacy' approach to inspire reflection on programme lessons in English classes. Another approach to integration emphasised *local development*, where interventions supported teachers to link health education across academic subjects in each school in a 'local' fashion. For example, in PATHS, teachers received suggestions on how to integrate programme learning across English, history and social studies lessons, while in Second Step, this was an encouraged aspect of classroom delivery. In both cases, teachers received guidance and support to integrate health education messages into academic education, but were given substantial latitude to determine how and when to do this in the school day. A third approach was *linking to developmental concerns*, emphasising not so much the comprehensive integration of academic and health education but rather the interrelationships between academic success and broader development, health and wellbeing. These interventions viewed academic education through a 'health' lens, in addition to viewing health education through an 'academic' lens. From a conceptual perspective, this meant that the interrelationships between academic achievement, and student health and wellbeing were emphasised in theories of change. From a practical perspective, this meant that interventions paired activities such as study skills lessons with social-emotional learning (e.g., in PATHS). For example, the theory of change underlying

For peer review only

Gatehouse related to the creation of healthy social milieus in schools that would also support academic attainment; practically, this manifested as enhancement of academic lessons to improve interpersonal skills and emotional regulation. Similarly, Positive Action tied together individual student attainment with student health and wellbeing in their theory of change, with lessons focused on problem-solving and goal-setting, among other topics.

Domains of integration

Some interventions (4Rs, Bullying Literature Project and Youth Matters) were exclusively *classroom-focused* while others (Gatehouse, Steps to Respect) used *classroom and whole-school* strategies to reinforce and extend learning. For example, Gatehouse involved school implementation-support teams, while Steps to Respect deployed a schoolwide 'policy team' to revise and develop anti-bullying policies. Other interventions, (PATHS, Positive Action) used *classroom, whole school environment and external domain* (parent information) strategies consistent with the health promoting schools approach promulgated particularly by the WHO, which in the US is known as the Comprehensive School Health Program (CSHP) model.[38]

Degree of integration

In some interventions, health education was fully integrated (woven seamlessly) into everyday academic lessons (Gatehouse, 4Rs, Youth Matters), while in partially integrated interventions, health education involved distinct lessons, albeit also covering academic learning (Positive Action).

Timing of integration

Most interventions were multi-year, though two involved only one school year (LIFT, Bullying Literature Project).

Intervention effects

For peer review only

Perpetration measures included bullying (physical, or physical/verbal), aggression against peers and others, and violent behaviours including injuring others. Measures involved different raters, including students, teachers and observers. Victimisation measures ranged from physical violence specifically to interpersonal aggression more generally. Heterogeneity of definition, measurement and form of effect sizes precluded meta-analysis. No included studies described effects for KS1 or KS5. Measures and corresponding effect estimates are included in Table 4.

Violence perpetration: KS2

Across the 10 evaluations reporting outcomes in this KS, effects were inconsistent, including within studies by rater.

In LIFT,[37] effects at the end of the first intervention year on observed physical aggression in the playground were similar for students with different levels of baseline aggression(d=-0.14 at mean, 1 SD and 2 SD above the pre-intervention mean); these findings being described as 'statistically significant'. However, after the first intervention year of 4Rs,[19] there were no effects on teacher-reported aggression (regression-estimated b=0.02, SE=0.05, based on a 1-4 scale). After the second intervention year,[15] there were effects on teacher-reported student aggression (d=-0.21, p<0.05). The Bullying Literature Project also reported no effects on physical aggression rated by teachers for individual students (IG: M=1.12, SD=0.47, n=95 vs. CG: 1.19, SD=0.47, n=55; p=0.67) or student self-reports (1.20, 0.44, n=90 vs. 1.14, 0.36, n=42; p=0.84) at one week post-intervention.[32] This finding was the same in the Bullying Literature Project—Moral Disengagement version (F(1, 80)=0.83, p=.431), though only combined student-reported physical and emotional bullying estimates were available.[39]

Findings for Steps to Respect differed by type of rater. At the end of the first intervention year, the first evaluation of Steps to Respect[35] reported evidence of decreased

bullying based on playground observation (F(91.3)=5.02, p<0.01) but not direct aggression based on student report (F(68.7)=2.05, p>0.05). The second evaluation of Steps to Respect[40] revealed a similar pattern. While teacher reports of physical bullying perpetration were less in intervention schools than in control schools at the end of the first intervention year (OR=0.61, t(29)=-3.12, p<0.01), student reports suggested no difference between schools on bullying perpetration (t(29)=-1.06). Moreover, in PATHS,[36] small positive effects of the intervention on student-reported aggression at the end of the first intervention year (d=-0.048, 95% CI [-0.189, 0.092]) and at the start (-0.064, [-0.205, 0.076]) and end (-0.048, [-0.188, 0.093]) of the second intervention year gave way to a small deleterious intervention effect at the end of the third year (0.082, [-0.060, 0.224]). Opposite effects were found on teacher-reported aggression, with initially small, negative intervention effects at the end of the first (0.036, [-0.105, 0.178]) and start of the second intervention year (0.035, [-0.107, 0.178]) but progressively greater effects at the end of the second (-0.005, [-0.146, 0.136]) and the third (-0.199, [-0.338, -0.060]) intervention years.

In contrast, two evaluations showed consistently positive results across different measures. In Positive Action Chicago,[34] students reported lower counts of bullying behaviours (IRR [incidence rate ratio] =0.59, 95% CI [0.37, 0.92]) and of serious violence-related behaviours, including cutting or stabbing someone on purpose (0.63, [0.45, 0.88]). Findings from Positive Action Hawaii[41] were similar for student-reported violent behaviours (IRR=0.42, 90% CI [0.24, 0.73]) and teacher-reported violent behaviours (0.54, [0.30, 0.77]). For students in the fourth or fifth intervention year, intervention recipients were less likely to report cutting or stabbing someone (OR=0.29, 90% CI [0.16, 0.52]) or shooting someone (0.24, [0.14, 0.40]). Teachers were less likely to report that students hurt others (0.61, [0.38, 0.97]) or got into lots of fights (0.63, [0.47, 0.84]).

For peer review only

However, in Youth Matters,[33] students in intervention schools were not less likely to report bullying perpetration (OR=0.85, 95% CI [0.29, 1.47], *p*=0.585) after the second intervention year. Evaluators explored use of latent class analyses to classify intervention recipients as victims, bullies or bully-victims. Proportions of intervention and control recipients classified as bullies or bully-victims were not significantly different by study arm at the end of the first (IG: 21%, n=356 vs CG: 22%, n=392) or second (19%, n=244 vs 23%, n=293) intervention years.[42]

Violence perpetration: KS3

The three evaluations examining violence perpetration outcomes in KS3 had dissimilar results. At the end of the sixth intervention year of Positive Action Chicago,[43] students receiving the intervention reported lower counts of violence-related behaviours than no-treatment controls (IRR=0.38, 95% CI [0.18, 0.81]; equivalent to *d*=-0.54). Students also reported fewer bullying behaviours (*d*=-0.39), and parents reported that their children engaged in fewer bullying behaviours (*d*=-0.31). Significance values for these estimates were not presented, but both were supported by significant condition-by-time interactions in multilevel models, indicating that the intervention group showed an improved trajectory over time as compared to the control group. In contrast, after the third year from baseline in Youth Matters,[42] proportions of students were not different in the collective bully and bully-victim groups (both groups 16%; IG n=283, CG n=289). Findings for Second Step were reported at the end of the first, second and third years of intervention. At the end of the first school year, students in intervention schools had decreased odds of physical aggression (OR=0.70, $p<0.05$) but not sexual harassment and sexual violence perpetration (OR=1.04, $p>0.05$). [31] These findings did not hold to the end of the second school year for physical aggression (OR=0.80, 95% CI [0.59, 1.08]), but sexual harassment and sexual violence perpetration was significantly reduced in intervention schools in Illinois (0.72, [0.54, 0.95])

but not Kansas (0.99, [0.71, 1.48]).[30] At the end of the third school year, there were no direct effects of Second Step on sexual harassment perpetration (β=0.005, SE=0.012); findings for physical aggression were not available.[29]

Violence victimisation: KS2

While the seven evaluations reporting outcomes in this KS were similar in follow-up period, they did not point to a clear effect. Students receiving the 'original' Bullying Literature Project were not different from their peers in physical victimisation by teacher report on individual students (IG: M=1.04, SD=0.23, n=95 vs. CG: 1.04, SD=0.21, n=55; p=0.39) or student self-report (1.35, 0.54, n=90 vs. 1.43, 0.66, n=42; p=0.57) one week post-intervention.[32] However, students receiving the Bullying Literature Project—Moral Disengagement version did report decrease in victimisation (both physical and emotional combined) after the intervention (IG: M=1.76, SD=0.81 to M=1.60, SD=0.66, n=42 vs. CG: M=1.23, SD=0.38 to M=1.38, SD=0.53, n=42), with a significant time-by-treatment interaction in an ANOVA (F(1, 80)=7.42, p=0.047).[39] PATHS measured student-reported victimisation using standardised mean differences, and found small, non-significant increases relative to the control arm at: the end of the first intervention year (d=0.044, 95% CI [-0.098, 0.185]); the start (0.074, [-0.067, 0.216]) and end (0.092, [-0.050, 0.234]) of the second year; and the end of the third year (0.089, [-0.053, 0.231]) of intervention implementation.[36] Steps to Respect, evaluated in two different trials, also found no differences in student-reported bullying victimisation at the end of the first intervention year in the first (IG: M=0.80, SD=1.51 vs CG: M=0.86, SD=1.44; F<1)[35] or second trial (2.11, 1.03 vs. 2.18, 1.06; t(29)=-1.15).[40] The first trial included playground observation at the end of the first intervention year, which was suggestive of lower levels in bullying victimisation, though these differences were marginally non-significant (0.9, 0.82 vs. 1.01, 0.83; F(72.4)=3.74, p<0.10).[35] Learning to Read in a Healing Classroom examined relational and physical

For peer review only

victimisation after one year of intervention implementation and found no significant effect of the intervention (weighted d=-0.01, SE=0.06).[44, 45] Finally, Youth Matters examined bullying victimisation through continuous and dichotomous measures. At the end of the second intervention year, the difference in log-transformed continuous scores suggested a decrease (difference=-0.171, SE=0.083, p=0.049), as did the difference in dichotomous scores (OR=0.61, p=0.098).[33] However, a latent class analysis that sought to describe transitions into, and out of, bullying victimisation did not suggest a difference between groups at this point.[42]

Violence victimisation: KS3 and KS4

Intervention evaluations reporting violence victimisation outcomes in KS3 (Youth Matters [42, 46], Second Step[30, 31] and Gatehouse[28]) and KS4 (Gatehouse[28]) suggested no evidence of effectiveness. In Youth Matters, differences in the log-transformed scores for bullying victimisation suggested a decrease in victimisation in intervention recipients as compared to controls, but this difference was not significant (regression-estimated difference=-0.123, SE=0.068, p=0.08).[46] However, at the end of the third intervention year, fewer students in the intervention than control group were members of the victim or bully-victim classes (36%, n=283 vs 45%, n=289).[42] Based on our own chi-square test, this difference was significant (p=0.029). In Second Step, neither peer victimisation (OR=1.01, p>0.5) nor sexual harassment and violence victimisation (OR=1.01, p>0.05) were different between students in intervention schools and control schools after the first intervention year;[31] this remained the case at the end of the second intervention year (peer victimisation: OR=0.94, 95% CI [0.75, 1.18]; sexual victimisation: 0.91, [0.72, 1.15]).[30] Gatehouse,[28] which was implemented from year 9, found no evidence of a change in bullying victimisation at the end of the first (OR=1.03, 95% CI [0.86, 1.26]),

second (1.03, [0.78, 1.34]) or third (0.88, [0.68, 1.13]) intervention years, which corresponded to the first two years of KS4.

DISCUSSION

While the integration of academic and health education remains a promising model for the delivery of school-based health education, randomised evaluations were variable in quality and did not consistently report evidence of effectiveness in reducing violence victimisation or perpetration. Evidence was concentrated in KS2, with few evaluations in KS3 or KS4. Moreover, evidence was stronger in quantity and in quality for violence perpetration as compared to victimisation. Unfortunately, evaluations that measured perpetration did not always also measure victimisation, preventing a meaningful comparison of consistency of effects.

Few interventions showed consistent signals of effectiveness. Though a formal moderator analysis was not possible, certain intervention models appear more effective than others. Specifically, evaluations of Positive Action in both Chicago[43] and Hawaii[41] showed consistently positive results across diverse measures. This may reflect the involvement of the intervention developer, a factor often associated with improved intervention fidelity (although Positive Action was not unique in this respect among interventions included in our review). It may also reflect that Positive Action included classroom, whole-school and (in the Hawaii trial) external domain strategies delivered over multiple school years. Though Gatehouse[28] was similar to Positive Action in its focus on multiple systems, Gatehouse targeted adolescents, whereas Positive Action was delivered from KS2 and also included work with parents. Another possible explanation for our results is that effects for these interventions may take time to emerge. This is plausible given the developmental focus of many of these interventions, and evidence of links between early aggressive behaviour and later violence.[4, 5] For example, there was some evidence that

effects on aggressive behaviour in 4Rs began to emerge after the second intervention year.[19] While findings were somewhat contradictory across different outcomes for PATHS, there was some evidence that teachers of intervention students reported less aggression in later years of the intervention.[36] Another key feature of Positive Action was the use of a model that linked academic and health education to developmental concerns. That is to say, this intervention focused on improvements in academic engagement and study skills both enhancing, and being enhanced by, student health and wellbeing; this was a feature of intervention activities and of the underlying theory of change. Moving forward, intervention strategies that combine multiple domains over several years and that use both subject-specific learning alongside linking to developmental concerns may be more effective than classroom-only interventions, single-year interventions, or interventions that use literature alone; this should be a target for future research.

This systematic review has strengths and limitations. Identifying relevant studies was challenging often because of poor intervention description. We were unable to undertake meta-analysis or assessment of publication bias, though the preponderance of null results suggests that projects with non-significant findings are being published. Finally, the diversity of outcome measures and of raters precludes a complete and consistent picture of the effectiveness of these interventions via standardised measures. For example, measures that included physical violence and aggression were at times combined with verbal forms of interpersonal violence; while we preferred measures of physical violence and physical aggression, we included outcomes where these behaviours were included as part of a composite. Consistency and clarity in outcome reporting will be especially important as 'core outcome sets' become relevant in planning evaluations in public health and social science. Most studies focused on bullying, while evaluations of Positive Action[41, 43] generally provided the most direct test of violent behaviours specifically.

For peer review only

Future research should seek to understand better the life course aspects of these interventions: that is, how does early school-based intervention impact later-life violent behaviours? From a policy perspective, it is clear that the integration of academic and health education, while possibly an effective intervention, will need to be considered alongside interventions involving other systems to prevent violence. Future evaluations will also contribute by considering the effects of integration in a diversity of ways and mechanisms of action for integration in different types of academic education. For example, contrasts between full and partial integration, which included evaluations did not address, could inform an understanding of how much integration is necessary to support health education messages.

FUNDING

This work was funded through National Institute for Health Research Public Health Research Programme grant 14/52/15.

The work was undertaken with the support of The Centre for the Development and Evaluation of Complex Interventions for Public Health Improvement (DECIPHer), a UKCRC Public Health Research Centre of Excellence. Joint funding (MR/KO232331/1) from the British Heart Foundation, Cancer Research UK, Economic and Social Research Council, Medical Research Council, the Welsh Government and the Wellcome Trust, under the auspices of the UK Clinical Research Collaboration, is gratefully acknowledged.

COMPETING INTERESTS

Authors have no competing interests to disclose.

CONTRIBUTIONS

GJMT undertook study screening and selection, led the meta-analyses and drafted the initial manuscript. TT undertook study screening and selection, extracted data and contributed to drafting the initial manuscript. AF undertook study screening and selection. JT and RC provided methodological and substantive advice. CB undertook study screening and

For peer review only

selection, extracted data, and contributed to drafting the initial manuscript. All authors revised the manuscript and approved the final manuscript as submitted.

ACKNOWLEDGEMENTS

The authors acknowledge Ms Claire Stansfield for her assistance in designing and conducting the searches.

DATA SHARING STATEMENT

All data are publicly available.

For peer review only

References

1 Krug EG, Mercy JA, Dahlberg LL*, et al.* The world report on violence and health. *The Lancet* 2002;**360**:1083-8.
2 Scott S, Knapp M, Henderson J*, et al.* Financial cost of social exclusion: follow up study of antisocial children into adulthood. *BMJ* 2001;**323**:191.
3 Beinart S, Anderson B, Lee S*, et al. Youth at Risk?: A National Survey of Risk Factors, Protective Factors and Problem Behaviour among Young People in England, Scotland and Wales (JRF Findings 432)*. York: Joseph Rowntree Foundation 2002.
4 Bender D, Lösel F. Bullying at school as a predictor of delinquency, violence and other anti-social behaviour in adulthood. *Criminal Behaviour and Mental Health* 2011;**21**:99-106.
5 Olweus D. Bullying at school: What we know and what can we do. *Malden, MA: Blackwell* 1993.
6 Farrington DP, Ttofi MM. *School-based programs to reduce bullying and victimization*: Campbell Systematic Reviews 2010.
7 Hahn R, Fuqua-Whitley D, Wethington H*, et al.* Effectiveness of universal school-based programs to prevent violent and aggressive behavior: A systematic review. *American Journal of Preventive Medicine* 2007;**33**:S114-S29.
8 Vreeman RC, Carroll AE. A systematic review of school-based interventions to prevent bullying. *Archives of Pediatrics & Adolescent Medicine* 2007;**161**:78-88.
9 Fletcher A, Bonell C, Sorhaindo A. "We don't have no drugs education": the myth of universal drugs education in English secondary schools? *International Journal of Drug Policy* 2010;**21**:452-8.
10 NASUWT. *English Baccalaureate Survey Summary*. Birmingham: NASUWT 2011.
11 PSHE Association. *Comments on the National Curriculum proposals published in February 2013 from the PSHE education Strategic Partners' Group*. PHSEA 2013.
12 The Office for Standards in Education Children's Services and Skills. School inspection handbook: Handbook for inspecting schools in England under section 5 of the Education Act 2005. Manchester: The Government of the United Kingdom 2016.
13 Bier MC, Zwarun L, Warren VF. Getting Universal Primary Tobacco Use Prevention Into Priority Area Schools A Media Literacy Approach. *Health Promotion Practice* 2011;**12**:152S-8S.
14 Pearson M, Chilton R, Wyatt K*, et al.* Implementing health promotion programmes in schools: a realist systematic review of research and experience in the United Kingdom. *Implementation Science* 2015;**10**:1.
15 Jones SM, Brown JL, Lawrence Aber J. Two-year impacts of a universal school-based social-emotional and literacy intervention: An experiment in translational developmental research. *Child Development* 2011;**82**:533-54.
16 Masten AS, Cicchetti D. Developmental cascades. *Development and Psychopathology* 2010;**22**:491-5.
17 Goleman D. *Emotional intelligence*. New York: Bantam 1995.
18 Jones SM, Brown JL, Aber JL. Three Year Cumulative Impacts of the 4Rs Program on Children's Social-Emotional, Behavioral, and Academic Outcomes. *Society for Research on Educational Effectiveness* 2010.
19 Jones SM, Brown JL, Hoglund WL*, et al.* A school-randomized clinical trial of an integrated social–emotional learning and literacy intervention: Impacts after 1 school year. *Journal of Consulting and Clinical Psychology* 2010;**78**:829.
20 The British Heart Foundation. Money to Burn lesson plan. 2014.

For peer review only

21 Wright G, Ainsworth P. Plastered evaluation: part of It's Not OK! Violence Prevention Education Programme. Liverpool: Ariel Trust 2008.
22 Flay BR, Graumlich S, Segawa E*, et al.* Effects of 2 prevention programs on high-risk behaviors among African American youth: a randomized trial. *Archives of Pediatrics & Adolescent Medicine* 2004;**158**:377-84.
23 Kupersmidt JB, Scull TM, Benson JW. Improving media message interpretation processing skills to promote healthy decision making about substance use: the effects of the middle school media ready curriculum. *Journal of Health Communication* 2012;**17**:546-63.
24 Patton G, Bond L, Carlin JB*, et al.* Promoting social inclusion in schools: group-randomized trial of effects on student health risk behaviour and well-being. *American Journal of Public Health* 2006;**96**:1582-7.
25 Peersman G, Oliver S, Oakley A. EPPI-Center review guidelines: data collection for the EPIC database. London: EPPI-Centre Social Science Research Unit 1997.
26 Higgins JPT, Green S. *Cochrane Handbook for Systematic Reviews of Interventions Version 5.1.0 [updated March 2011]*. Oxford: The Cochrane Collaboration 2011.
27 Sutcliffe K, Thomas J, Stokes G*, et al.* Intervention component analysis (ICA): a pragmatic approach for identifying the critical features of complex interventions. *Systematic Reviews* 2015;**4**:140.
28 Bond L, Patton G, Glover S*, et al.* The Gatehouse Project: can a multilevel school intervention affect emotional wellbeing and health risk behaviours? *Journal of epidemiology and community health* 2004;**58**:997-1003.
29 Espelage DL, Low S, Van Ryzin MJ*, et al.* Clinical trial of Second Step Middle School Program: Impact on bullying, cyberbullying, homophobic teasing, and sexual harassment perpetration. *School Psychology Review* 2015;**44**:464-79.
30 Espelage DL, Low S, Polanin JR*, et al.* Clinical trial of Second Step© middle-school program: Impact on aggression & victimization. *Journal of Applied Developmental Psychology* 2015;**37**:52-63.
31 Espelage DL, Low S, Polanin JR*, et al.* The Impact of a Middle School Program to Reduce Aggression, Victimization, and Sexual Violence. *Journal of Adolescent Health* 2013;**53**:180-6.
32 Couch L. The Bullying Literature Project: An Evaluation of a Class-Wide Bullying Intervention Program. *Education*. California: University of California Riverside 2015.
33 Jenson JM, Dieterich WA. Effects of a skills-based prevention program on bullying and bully victimization among elementary school children. *Prevention Science* 2007;**8**:285-96.
34 Li K-K, Washburn I, DuBois DL*, et al.* Effects of the Positive Action programme on problem behaviours in elementary school students: A matched-pair randomised control trial in Chicago. *Psychology and Health* 2011;**26**:187-204.
35 Frey KS, Hirschstein MK, Snell JL*, et al.* Reducing playground bullying and supporting beliefs: an experimental trial of the steps to respect program. *Developmental Psychology* 2005;**41**:479.
36 Crean HF, Johnson DB. Promoting Alternative Thinking Strategies (PATHS) and elementary school aged children's aggression: Results from a cluster randomized trial. *American Journal of Community Psychology* 2013;**52**:56-72.
37 Reid JB, Eddy JM, Fetrow RA*, et al.* Description and immediate impacts of a preventive intervention for conduct problems. *American Journal of Community Psychology* 1999;**27**:483-518.
38 Langford R, Bonell CP, Jones HE*, et al.* The WHO Health Promoting School framework for improving the health and well-being of students and their academic achievement. *The Cochrane Database of Systematic Reviews* 2014;**4**:CD008958.

For peer review only

39 Wang C, Goldberg TS. Using children's literature to decrease moral disengagement and victimization among elementary school students. *Psychology in the Schools* 2017:No-Specified.
40 Brown EC, Low S, Smith BH*, et al.* Outcomes from a school-randomized controlled trial of steps to respect: A bullying prevention program. *School Psychology Review* 2011;**40**:423.
41 Beets MW, Flay BR, Vuchinich S*, et al.* Use of a social and character development program to prevent substance use, violent behaviors, and sexual activity among elementary-school students in Hawaii. *American Journal of Public Health* 2009;**99**:1438-45.
42 Jenson JM, Brisson D, Bender KA*, et al.* Effects of the Youth Matters prevention program on patterns of bullying and victimization in elementary and middle school. *Social Work Research* 2013:svt030.
43 Lewis KM, Schure MB, Bavarian N*, et al.* Problem behavior and urban, low-income youth: A randomized controlled trial of Positive Action in Chicago. *American Journal of Preventive Medicine* 2013;**44**:622-30.
44 Aber JL, Tubbs C, Torrente C*, et al.* Promoting children's learning and development in conflict-affected countries: Testing change process in the Democratic Republic of the Congo. *Development and Psychopathology* 2017;**29**:53-67.
45 Torrente C, Johnston B, Starkey L*, et al.* Improving the Quality of School Interactions and Student Well-Being: Impacts of One Year of a School-Based Program in the Democratic Republic of the Congo. *Journal on Education in Emergencies* 2015;**1**:48-91.
46 Jenson JM, Dieterich WA, Brisson D*, et al.* Preventing childhood bullying: Findings and lessons from the Denver public schools trial. *Research on Social Work Practice* 2010.

For peer review only

Figures

Figure 1. PRISMA flowchart

For peer review only

Table 1. Characteristics of included studies

Evaluation, setting and studies	Sample characteristics	Intervention description	Control description
**Bullying Literature Project**<br><br>California, USA<br><br>Couch 2015[32]	4 classrooms, 95 teacher reports, 90 students (IG); 3 classrooms, 55 teacher reports, 42 students (CG)<br><br>Students enrolled in years 4 and 5, followed up one week post-intervention<br><br>42.8% female, 57.2% male<br><br>9.6% African American, 63.3% Hispanic, 9.0% Caucasian, 3.0% Asian, 4.2% other, 10.2% did not report<br><br>>50% of students received free or reduced-cost lunch	This intervention aims to reduce bullying by introducing themes related to bullying through children's literature. It also provides an opportunity for children to role-model practical skills to address or avoid bullying. The Bullying Literature Project integrates themes related to bullying into the children's literature used within a standard English curriculum. Students then had the opportunity to practice and reinforce skills via writing activities. The intervention was delivered by school psychologists supervised by the PI and lasted over five weeks in one school term. Additionally, the version including moral disengagement discussed the role of moral disengagement in each lesson as well.	Waitlist control
**Bullying Literature Project—Moral Disengagement**<br><br>California, USA<br><br>Wang 2017[39]	2 classrooms, 42 students (IG); 2 classrooms, 42 students (CG)<br><br>Students enrolled in year 4, followed up one week post-intervention<br><br>53.6% female, 46.4% male<br><br>2.4% Asian, 3.6% Caucasian, 94% Hispanic<br><br>SES details not reported		Waitlist control
**Gatehouse**	2 districts, 12 schools, 1335 students (IG);	Through teaching a curriculum (including integration of	Education as

For peer review only

Melbourne, Australia<br><br>Bond 2004[28]	2 districts, 14 schools, 1343 students (CG)<br><br>Students enrolled in Year 9, followed up for three years<br><br>53.2% female, 46.8% male<br><br>87.5% Australian-born<br><br>79.2% from two-parent family; 24.2% speak language other than English at home	cognitive behavioural principles in English classes) and establishing a school-wide adolescent health team, Gatehouse aims to: build a sense of security and trust in students; enhance skills and opportunities for good communication; and build a sense of positive regard through participation in school life. The intervention was delivered by teachers over the course of two school years, supported by the schoolwide adolescent health team and by external consultants who themselves were experiences teachers. Integration was achieved by using English classes to convey cognitive behavioural techniques for self-management, including via a 'critical literacy' approach that uses poetry, literature, song, film and visual materials.	usual
**Learning to Read in a Healing Classroom**<br><br>Democratic Republic of the Congo<br><br>Torrente 2015[45]<br>Aber 2017[44]	20 districts, 33 schools (IG); 19 districts, 30 schools (CG); 3,857 students overall<br><br>Students enrolled in Years 3-5, followed up for one year<br><br>48% female, 52% male	The intervention is delivered over the course of a year and is designed for use in post-conflict reconstruction areas. Teachers are supported to integrate social-emotional learning in literacy lessons, supported by a bank of lesson plans relating to reading and writing. Teachers additionally received substantial professional development, including 'teacher learning circles', and developed strategies to improve the learning environment.	Waitlist control
**Linking the Interests of Families and Teachers (LIFT)**<br><br>Pacific Northwest, USA<br><br>Reid 1999[37]	3 schools, 214 students (IG); 3 schools, 147 students (CG)<br><br>Students enrolled in year 6 and followed up over seven years<br><br>49% female, 51% male<br><br>86% White, 14% ethnic minority	Classroom instruction and discussion on specific social and problem-solving skills followed by skills practice, reinforced during free play using a group cooperation game with review of behaviour and presentation of daily rewards. There is also a parent evening to engage families and opportunities for parents to engage with teachers. The intervention was delivered by teachers and special instructors. Integration was achieved by teaching study skills alongside social-emotional	Education as usual

	12% mother less than high school graduate, 8% father less than high school graduate; 36% mother unemployed, 10% father unemployed; 22% single-parent families; 18% receiving benefits; 20% less than $15,000/year in early 1990s	education content, and was delivered over the course of one school year.	
**Positive Action Chicago**<br><br>Chicago, USA<br><br>Li 2011[34]<br>Lewis 2013[43]	7 schools, ~240 students (IG); 7 schools, ~260 students (CG)<br><br>Students enrolled in year 4 and followed up over six years<br><br>~48% female, ~52% male<br><br>55% African American, 32% Hispanic, 9% White non-Hispanic, 4% Asian, 5% other or mixed<br><br>83% receiving free lunch	Teachers provide lessons covering six units: self-concept; positive actions for mind and body; positive social-emotional actions; managing oneself; being honest with oneself; and continually improving oneself. Content includes 140 lessons per grade per year from years 1 to 13. In addition, an implementation coordinator and school climate team are appointed to support the intervention. The intervention is primarily delivered by teachers and school staff; in both trials, this was supported by extensive professional development and training. Integration was achieved by linking academic learning to social-emotional and health-related learning, e.g. by including content on problem solving and study skills alongside positive actions for mind and body, and by encouraging teachers to reflect Positive Action content in academic lessons.	Education as usual
**Positive Action Hawaii**<br><br>Hawaii, USA<br><br>Beets 2009[41]	10 schools, 976 students (IG); 10 schools, 738 students (CG)<br><br>Students enrolled in years 2 or 3 and followed up over four or five years<br><br>50% female, 50% male<br><br>26.1% Hawaiian, 22.6% mixed, 8.6% White, 1.6% African American, 1.7% American Indian, 4.7% other Pacific Islander, 4.6% Japanese, 20.6% other		Education as usual

For peer review only

	Asian, other 7.8%, unknown 1.6%.<br><br>Control schools had on average 55% free/reduced lunch students, whereas intervention schools had on average 56% free/reduced lunch students		
**Promoting Alternative Thinking Strategies (PATHS)**<br><br>Minnesota and New York State, USA<br><br>Crean 2013[36]	7 schools, 422 students (IG); 7 schools, 357 students (CG)<br><br>Students enrolled in year 4 and followed up over three years<br><br>57% female, 43% male<br><br>51% White, 38% African American, 10% other, 17% Hispanic<br><br>33% from single parent homes; 39% families with income less than $20,000/year, 43% below the federal poverty line; 11% no parent with high school diploma	An intervention to reduce conflict by improving students' social-emotional and thinking skills through a curriculum (including study skills), the establishment of a positive classroom environment and generalised positive social norms throughout the school environment. Lessons are grouped into three units addressing readiness and self-control, feelings and relationships, and interpersonal problem solving. These units cover five domains: 1. Self-control; 2. Emotional understanding; 3. Positive self-esteem; 4. Healthy relationships; and 5. Interpersonal problem-solving skills. The intervention is delivered by teachers supported by consultants, with 131 lessons delivered over three years (two to three times per week, 20 to 30 minutes each). Integration was achieved by linking study skills to social-emotional learning, by supporting teachers to include children's literature in reinforcing concepts, and by providing ideas to link PATHS to English, social studies and history lessons.	Education as usual
**Reading, Writing, Respect and Reconciliation (4Rs)**<br><br>New York City, USA	9 schools, 515 students (IG); 9 schools, 427 students (CG)<br><br>Students enrolled in year 4 followed up for three years (only results up to two years available)	This intervention includes two components: 1. a seven-unit, 21–35 lesson literacy-based curriculum in conflict resolution and social-emotional learning for children in primary school (from year 1 to year 6); and 2. intensive professional development for teachers. The intervention was delivered by teachers after this extensive professional development. Integration was achieved by	Education as usual

For peer review only

Jones 2010[19]<br>Jones 2011[15]	51.2% female, 48.8% male<br><br>41.1% African American, 45.6% Hispanic, 4.7% Caucasian, 8.6% other<br><br>31% low parental education, 15.1% parental unemployment, 53.4% single-parent household, 61.8% living in poverty	using literature as a springboard to help students understand anger and develop skills in listening, cooperation, assertiveness and negotiation.	
**Second Step**<br><br>Illinois and Kansas, USA<br><br>Espelage 2013[31]<br>Espelage 2015a[30]<br>Espelage 2015b[29]	18 schools, 1,940 students (IG); 18 schools, 1,676 students (CG)<br><br>Students enrolled in year 7, followed up yearly over three years<br><br>48.1% female, 51.9% male<br><br>26.4% African American, 24.7% Caucasian, 34.2% Hispanic, 14.7% biracial and all others<br><br>74.1% free or reduced lunch	This intervention includes 15 weeks of classroom lessons taught weekly or every two weeks throughout the school year for three years. Teachers are supported by professional development training to deliver intervention content, which includes bullying, problem-solving, emotional regulation and empathy, alongside videos. Teachers also receive plans to support integration of Second Step content into academic lessons. Modelling, role-play and coaching are included in the intervention. Students receive homework to reinforce skills, and use group and collaborative work to practice skills.	Education as usual, with additional bullying resources
**Steps to Respect I**<br><br>Pacific Northwest, USA<br><br>Frey 2005[35]	3 schools (IG), 3 schools (CG); 1,126 students total<br><br>Students enrolled in years 4 through 7; followed up for one year in endpoint-based analyses<br><br>49.4% female, 50.6% male<br><br>70.0% White, 9% African American, 12.7% Asian, 7.0% Hispanic, 1.3% Native	This is an anti-bullying intervention with both school-wide and classroom components. The School-wide components create new disciplinary policies for bullying and improve monitoring of and intervention in bullying. Classroom curricula positive social norms and improve social–emotional skills for better engagement with bullying. The intervention was delivered by classroom teachers alongside schoolwide bullying policy teams. Biweekly lessons in the Steps to Respect curriculum are supported by 8-to-10 literature-based lessons presented over a 12 to 14 week period. This	Waitlist control

For peer review only

	American<br><br>SES indices not stated	intervention integrates academic and health education by developing literacy skills alongside furthering understanding of the Steps to Respect curricular themes.	
**Steps to Respect II**<br><br>North-Central California, USA<br><br>Brown 2011[40]	17 schools (IG), 16 schools (CG); 2,940 students total<br><br>Students enrolled in years 4 through 6; followed up for one year<br><br>51% female, 49% male (IG); 48% female, 52% male (CG)<br><br>52% White, 7% African American, 6% Asian, 43% Hispanic, 35% other or mixed race (IG); 53% White, 6% African American, 6% Asian, 41% Hispanic, 35% other or mixed race (CG)<br><br>School-level average of 40% on free or reduced-price lunch		Waitlist control
**Youth Matters**<br><br>Denver, USA<br><br>Jenson 2007[33]<br>Jenson 2010[46]<br>Jenson 2013[42]	14 schools, 702 students (IG), 14 schools, 462 students (CG)<br><br>Students enrolled in year 5 and followed up for three years<br><br>50.6% female, 49.4% male<br><br>59.1% Latinx, 14.7% African American, 16.8% American Indian, Asian American, or mixed, 9.3% Caucasian	Youth Matters promotes the development of healthy relationships and social competency and the development of social resistance. Classroom discussions around social issues promote positive social norms. Over four modules with 10 lessons, delivered over two years, students read age-appropriate stories, receive social-emotional learning and practice skills. The intervention was delivered by educational specialists from outside the school. Integration was achieved by using 30-40 page stories in each module intended to support schools in meeting academic standards in academic and health education.	Education as usual

For peer review only

	SES indices not reported		

For peer review only

Table 2. Appraisal of included studies

Intervention name	Random generation of allocation sequence	Concealed allocation	Blinding	Complete outcome data	Reporting not selective	Controlled for confounding	Accounted for clustering	Reduced other forms of bias	Suitable control group
Bullying Literature Project	NC	NS	NS	Yes	NC	NC	NS	NS	NC
Bullying Literature Project—Moral Disengagement	NS	NS	NS	Yes	Yes	Yes	No	Yes	Yes
Learning to Read in a Healing Classroom	Yes	Yes	NS	No	Yes	Yes	Yes	Yes	Yes
Linking the Interests of Families and Teachers (LIFT)	Yes	NS	Yes	Yes	NC	Yes	Yes	Yes	NC
Positive Action Hawaii	NC	NS	NS	Yes	NC	NC	Yes	Yes	Yes
Positive Action Chicago	Yes	NS	NS	Yes	NC	Yes	Yes	Yes	Yes
Promoting Alternative Thinking Strategies (PATHS)	NC	NS	NS	Yes	NC	NC	Yes	Yes	NC
Reading Writing, Respect and Resolution (4Rs)	Yes	NS	NS	Yes	No	Yes	Yes	Yes	Yes
Second Step	Yes	NS	NS	Yes	No	Yes	Yes	Yes	Yes
Steps to Respect I	NC	NS	NS	Yes	NC	NC	Yes	Yes	NC
Steps to Respect II	NC	NS	NS	Yes	NC	NC	Yes	Yes	Yes
Gatehouse	NC	NS	NS	Yes	NC	Yes	Yes	Yes	Yes

For peer review only

Youth Matters	NC	NS	NS	Yes	NC	Yes	Yes	NS	Yes

Legend: NC = not clear; NS = not stated

For peer review only

Table 3. Key themes in the intervention components analysis.

Key theme	Components within theme	Bullying Literature Project	Gatehouse	Learning to Read in a Healing Classroom	LIFT	Positive Action	PATHS	4Rs	Second Step	Steps to Respect	Youth Matters
Approach to integration	Literature: did interventions use literature and language arts as the key vehicle for delivery? Local development: did interventions support teachers to link health education across academic subjects in each school? Linking to developmental concerns: did interventions link academic education and personal health and development?	**Literature:** use of children's books as basis for lessons	**Literature:** English classes used as a key vehicle for delivery of cognitive behavioural content relating to emotional learning and self-regulation **Linking to developmental concerns:** underlying the theory of change was a connection between school health and wellbeing and academic attainment, and the need to create 'healthy' schools	**Literature:** use of reading and language arts lessons to support socio-emotional learning by providing lesson plans	**Linking to developmental concerns:** study skills were presented alongside socio-emotional learning skills, such as empathy and how to play with peers. This content was restricted to the year 6 arm of the intervention	**Local development:** teachers are supported to integrate health education lessons (both social-emotional learning and health and wellbeing, e.g. hygiene) throughout academic learning **Linking to developmental concerns:** key aspect of theory of change is linking academic achievement with physical and mental health and wellbeing, character development	**Linking to developmental concerns:** content on social-emotional learning was presented alongside study skills in later years of the programme **Literature:** English (but also history and social studies classes) was used as a key opportunity to reinforce concepts taught in discrete manualised social-emotional learning lessons	**Literature:** the intervention centres on a literacy-based curriculum relating conflict resolution and social-emotional learning to children's literature	**Local development:** social-emotional learning is integrated into academic lessons alongside a manualised programme of content relating specifically to bullying, problem-solving, emotional regulation content and multimedia resources	**Literature:** the classroom component of this intervention relates to a programme of literature-based lessons designed to convey anti-bullying messages	**Literature:** stories are used to discuss healthy relationships, resistance to bullying and aggressive behaviours, and to practice skills, including via projects relating to literacy lessons
Domains of integration	Classroom: did interventions focus on the classroom? Classroom and whole-school: did interventions include whole-school change components	**Classroom:** focus on classroom teaching only via book-reading and accompanying activities	**Classroom and whole-school domains:** in addition to classroom learning strategies, a school health team supported by eternal	**Classroom and whole-school domains:** in addition to lessons plans to support classroom teaching, pedagogic	**Classroom, whole-school and external domains:** in addition to supporting study skills alongside social-emotional learning,	**Classroom, whole-school and external domains:** in addition to extensively manualised lessons, a school climate team was assembled as part of the	**Classroom, whole-school and external domains:** manualised lessons relating to social-emotional learning and self-regulation are	**Classroom:** teachers receive substantial professional development to implement the intervention using specific materials	**Classroom:** intervention delivered in the classroom context specifically	**Classroom and whole-school domains:** in addition to classroom literacy-based learning, a whole-school	**Classroom and whole-school domains:** lessons delivered in the classroom context, but whole-school events

For peer review only - http://bmjopen.bmj.com/site/about/guidelines.xhtml

	alongside classroom components? Classroom, whole-school and external domains: did interventions also include parent engagement alongside classroom and whole-school components?		consultants sought to identify ways to improve school climate to promote health and wellbeing	circles facilitated school meetings led to exchange of ideas on how to improve school climate	parents received a series of parenting classes and teachers were encouraged to communicate with parents via a phone line recorded message	intervention with a schoolwide 'champion' for intervention implementation. Parents are also involved through homework and 'take-home' assignments, as well as community engagement, though this was not a feature in the Chicago trial	accompanied with school-wide implementation to promote generalised positive norms and parent information	prepared as part of the intervention		policy team developed schoolwide responses to bullying	'showcasing' work part of the intervention activities
Degree of integration	Did interventions include full or partial integration of health education alongside academic education?	**Full integration:** lessons designed to develop literacy skills	**Full integration:** the use of 'critical literacy' to convey social-emotional learning was seamlessly integrated into English classes	**Full integration:** lessons designed to integrate social-emotional learning into enhanced provision of reading and literacy	**Partial integration:** the intervention was set apart from other academic learning	**Partial integration:** discrete lessons relating to Positive Action are presented as part of the intervention	**Partial integration:** manualised intervention lessons presented alongside academic content	**Full integration:** learning is presented alongside literature and reading lessons	**Partial integration:** separate lessons for intervention content are presented alongside integration	**Full integration:** lessons designed to address key literacy goals	**Full integration:** intervention 'led' by literacy and literature content
Timing of integration	Were interventions one year or multiple years in duration?	One year	Multiple years	Multiple years	One year	Multiple years	Multiple years	Multiple years	Multiple years	Multiple years/one year	Multiple years

For peer review only

Table 4. Measures used in included studies and effect estimates

Evaluation	Measure	Notes	Effect estimate
**Violence perpetration**			
4Rs	Aggression	Frequency score on 13 aggressive behaviours assessed by teacher report in last month, including physical aggression and threatening of others	**KS2**<br>End of first year: regression-estimated *b*=0.02, SE=0.05, based on a 1-4 scale<br>End of second year: *d*=-0.21, *p*<0.05
Bullying Literature Project	Physical bullying	Assessed by teacher and student report; mean of frequency scores relating to reports of violence	**KS2**<br>Teacher report: IG: M=1.12, SD=0.47, n=95 vs. CG: 1.19, SD=0.47, n=55; *p*=0.67<br>Student report: 1.20, 0.44, n=90 vs. 1.14, 0.36, n=42; *p*=0.84
Bullying Literature Project—Moral Disengagement	Bullying	Assessed by student report; mean of frequency scores relating to physical and emotional bullying	**KS2**<br>No significant difference from time by treatment interaction: F(1, 80)=0.83, p=.431
LIFT	Change in child physical playground aggression	Measured by observation; includes physical bullying by observed children	**KS2**<br>'Statistically significant' differences: *d*=-0.14 at mean, 1 SD and 2 SD above the pre-intervention mean
PATHS	Aggression	Assessed by teacher and student report; mean of frequency scores relating to verbal and physical aggression	**KS2**<br>Student report: decrease at end of first year *d*=-0.048, 95% CI (-0.189, 0.092); start of second year (-0.064, [-0.205, 0.076]); end of second year (-0.048, [-0.188, 0.093]); but increase end of the third year (0.082, [-0.060, 0.224])<br>Teacher report: increase at end of the first year (0.036, [-0.105, 0.178]), start of second year (0.035, [-0.107, 0.178]) but decrease end of second year (-0.005, [-0.146, 0.136]) and end of third year (-0.199, [-0.338, -0.060])

Positive Action Chicago	Bullying	Student report: count of bullying behaviours relating to verbal or physical aggression behaviours in the past two weeks<br>Parent report: count of observed verbal or physical aggression behaviours in past 30 days	**KS2**<br>Student report IRR [incidence rate ratio] =0.59, 95% CI (0.37, 0.92)<br>**KS3**<br>Student report: *d*=-0.39<br>Parent report: *d*=-0.31
	Violence-related behaviours	Count of lifetime behaviours: carried a knife, threatened to cut or stab someone, cut of stabbed someone on purpose, been asked to join a gang, hung out with gang members, been a member of a gang	**KS2**<br>IRR=0.63, 95% CI [0.45, 0.88]<br>**KS3**<br>IRR=0.38, 95% CI [0.18, 0.81], or *d*=-0.54
Positive Action Hawaii	Count of violent behaviours	Teacher, student report	**KS2**<br>Teacher report: IRR=0.54, 90% CI [0.30, 0.77]<br>Student report: IRR=0.42, 90% CI [0.24, 0.73]
	Cut or stabbed others	Student report, lifetime prevalence	**KS2**<br>OR=0.29, 90% CI [0.16, 0.52]
	Shot another person	Student report, lifetime prevalence	**KS2**<br>OR=0.24, 90% CI [0.14, 0.40]
	Physically hurts others	Teacher report	**KS2**<br>OR=0.61, 90% CI [0.38, 0.97]
	Gets into a lot of fights	Teacher report	**KS2**<br>OR=0.63, 90% CI [0.47, 0.84]
Second Step	Physical aggression perpetration	Student report, endorse any fighting behaviours in last 30 days	**KS3**<br>End of first year: OR=0.70, $p<0.05$<br>End of second year: OR=0.80, 95% CI [0.59, 1.08]<br>End of third year: $\beta=0.005$, SE=0.012
	Sexual harassment and violence perpetration	Student report, endorse any verbal sexual violence or groping behaviours or forced sexual contact	**KS3**<br>End of first year: OR=1.04, $p>0.05$<br>End of second year: Illinois schools 0.72 [0.54, 0.95], Kansas schools 0.99 [0.71, 1.48]
Steps to Respect I	Bullying	Playground observation of students	**KS2**<br>Decrease in intervention group: $F(91.3)=5.02$, $p<0.01$
	Direct	Mean of student reported frequency scores of direct	Decrease not significant in intervention group

For peer review only

	aggression	bullying	compared to control: $F(68.7)=2.05$, $p>0.05$
Steps to Respect II	Bullying perpetration	Measured by student report; proportion of students with at least one bullying behaviour	**KS2**<br>Intervention group not significantly lower than control group: $t(29)=-1.06$
	Physical bullying perpetration	Measured by teacher report; proportion of students with at least one physical bullying behaviour	**KS2**<br>Significantly less in intervention group: OR=0.61, $t(29)=-3.12$, $p<0.01$
Youth Matters	Bullying	At least two or three times a month on at least one bullying behaviour	**KS2**<br>OR=0.85, 95% CI [0.29, 1.47], $p=0.585$
	Bully, victim, or bully-victim	Classification of students based on questionnaire responses into one of three categories	Bully or bully-victim<br>**KS2**<br>end of first year IG: 21%, n=356 vs CG: 22%, n=392; end of second year 19%, n=244 vs 23%, n=293<br>**KS3**<br>both groups 16%; IG n=283, CG n=289
**Violence victimisation**			
Bullying Literature Project	Physical bullying	Assessed by teacher and student report; mean of frequency scores relating to reports of violence	**KS2**<br>Teacher report: IG: M=1.04, SD=0.23, n=95 vs. CG: 1.04, SD=0.21, n=55; $p=0.39$<br>Student report: (1.35, 0.54, n=90 vs. 1.43, 0.66, n=42; $p=0.57$
Bullying Literature Project—Moral Disengagement	Bullying victimisation	Assessed by student report; mean of frequency scores relating to physical and emotional bullying	**KS2**<br>Student report: IG: M=1.76, SD=0.81 to M=1.60, SD=0.66, n=42 vs. CG: M=1.23, SD=0.38 to M=1.38, SD=0.53, n=42; $F(1, 80)=7.42$, $p=0.047$
Gatehouse	Bullying victimisation	Assessed by student report; any of being teased, having rumours spread about them, deliberate exclusion or experience of threats or violence	**KS4**<br>End of first year OR=1.03, 95% CI [0.86, 1.26]<br>End of second year OR=1.03, [0.78, 1.34]<br>End of third year OR=0.88, [0.68, 1.13]
Learning to Read in a Healing	Victimisation	Assessed by student report; average of frequency scores of peer verbal and physical bullying	**KS2**<br>weighted $d=-0.01$, SE=0.06

For peer review only

Classroom			
PATHS	Victimisation	Assessed by student report; sum of frequency scores of victimisation in last two weeks	**KS2**<br>Increase at the end of the first intervention year (*d*=0.044, 95% CI [-0.098, 0.185]); the start (0.074, [-0.067, 0.216]) and end (0.092, [-0.050, 0.234]) of the second year; and the end of the third year (0.089, [-0.053, 0.231])
Second Step	Peer victimisation	Student report, endorse any physical or verbal victimisation in last 30 days	**KS3**<br>End of first year OR=1.01, p>0.05<br>End of second year OR=0.94, 95% CI [0.75, 1.18]
	Sexual harassment and violence victimisation	Student report, endorse any victimisation by verbal sexual violence or groping behaviours or forced sexual contact	**KS3**<br>End of first year OR=1.01, *p*>0.05<br>End of second year OR=0.91, [0.72, 1.15]
Steps to Respect I	Target of bullying	Playground observation of students	**KS2**<br>IG: M=0.9, SD=0.82 vs. CG: M=1.01, SD=0.83; *F*(72.4)=3.74, *p*<0.10
	Victimisation	Assessed by student report; mean of frequency scores for physical and verbal victimisation items	**KS2**<br>IG: M=0.80, SD=1.51 vs CG: M=0.86, SD=1.44; *F*<1
Steps to Respect II	Victimisation	Assessed by student report; mean of frequency scores for physical and verbal victimisation items	**KS2**<br>IG: M=2.11, SD=1.03 vs. CG: M=2.18, SD=1.06; *t*(29)=-1.15
Youth Matters	Victimisation	Assessed by student report; mean of frequency scores for physical and verbal victimisation items, and also at least two or three times a month victimisation at least one bullying behaviour	**KS2**<br>difference=-0.171, SE=0.083, *p*=0.049; OR=0.61, *p*=0.098<br>**KS3**<br>regression-estimated difference=-0.123, SE=0.068, *p*=0.08
	Bully, victim, or bully-victim	Classification of students based on questionnaire responses into one of three categories	Victim or bully-victim<br>**KS2**<br>No difference between groups

For peer review only

			**KS3**<br>IG: 36%, n=283 vs CG: 45%, n=289

For peer review only

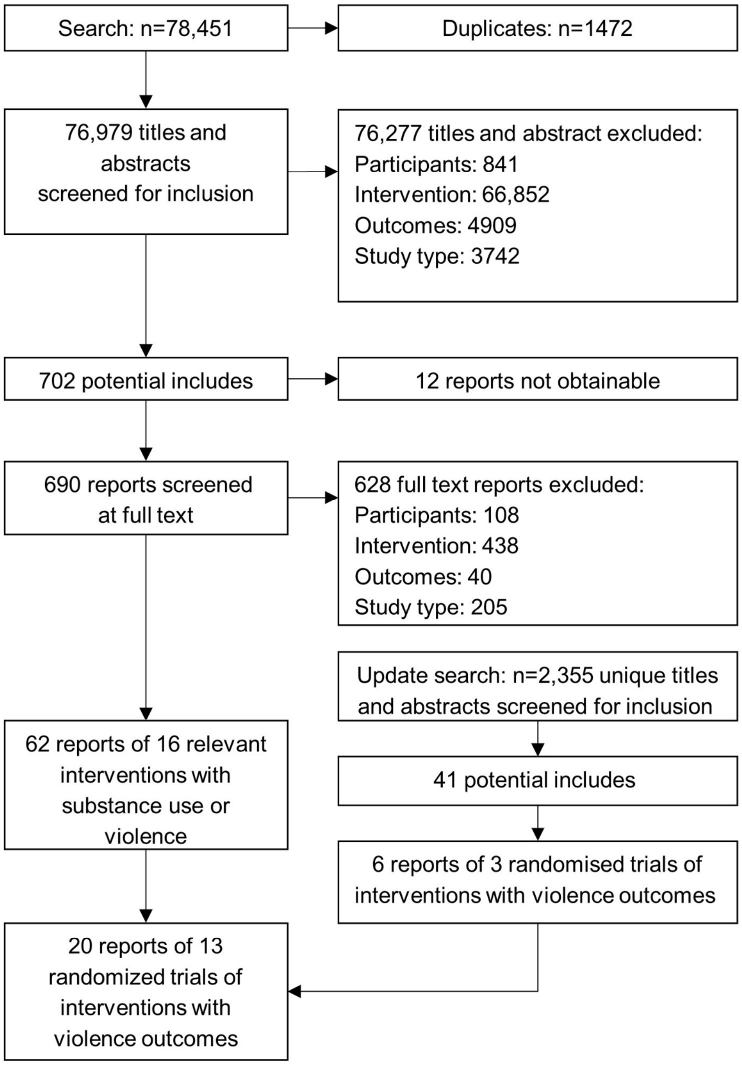

PRISMA flowchart

154x190mm (300 x 300 DPI)

PROSPERO
International prospective register of systematic reviews

National Institute for Health Research

How can interventions integrating health and academic education in schools help prevent substance misuse and violence, and reduce health inequalities among young people? Systematic review and evidence synthesis

Chris Bonell, James Thomas, Adam Fletcher, Rona Campbell, GJ Melendez-Torres, Claire Stansfield, Tara Tancred

Citation

Chris Bonell, James Thomas, Adam Fletcher, Rona Campbell, GJ Melendez-Torres, Claire Stansfield, Tara Tancred. How can interventions integrating health and academic education in schools help prevent substance misuse and violence, and reduce health inequalities among young people? Systematic review and evidence synthesis. PROSPERO 2015 CRD42015026464 Available from: http://www.crd.york.ac.uk/PROSPERO/display_record.php?ID=CRD42015026464

Review question

RQ1. What types of curriculum interventions integrating health and academic education in schools addressing substance use and violence have been evaluated?
RQ2. What theories of change inform these interventions and what do these suggest about their potential mechanisms and effects?
RQ3. What characteristics of interventions, deliverers, participants and school contexts facilitate or limit successful implementation and receipt of such interventions, and what are the implications of these for delivery in the UK?
RQ4. How effective are such interventions in reducing alcohol consumption, smoking, drug use and violence, and increasing attainment, when compared to usual treatment, no treatment, or other interventions, and does this vary according to students' socio-demographic characteristics?
RQ5. What characteristics of interventions, deliverers, school contexts and students appear to moderate or are necessary and sufficient for the effectiveness of such interventions?

Searches

Our search strategy will be informed by those used in previous systematic reviews focused on school interventions addressing alcohol, smoking, drug use and violence. The studies sought by this review are not likely to be reliably indexed in databases with controlled vocabularies. So we anticipate our searches involving a large number of free text terms. We will take the following three key concepts from the inclusion criteria to develop the search string: health education; integration with academic learning; and children and young people or schools. The combination of these concepts is sensitive enough to include all available studies regardless of study design. The three concepts will be linked by the Boolean operator "AND". Our searches will involve different free text and controlled vocabulary terms for each of these two concepts linked by the Boolean operator "OR". In our use of terms relating to health education, we will use a very broad array of terms to minimise the risk of publication bias. We will not restrict the searches by date, language or publication type. We will search the following databases from inception to present: ASSIA; Australian Educational Index; BiblioMap (Database of health promotion research); British Educational Index; Cochrane Central Register of Controlled Trials; Cochrane Database of Systematic Reviews; Database of Abstracts of Reviews of Effects; Database of Promoting Health Effectiveness Reviews; Econlit; ERIC; Health Technology Assessments; IBSS (International Bibliography of the Social Sciences); Medline; NHS Economic Evaluation Database; Proquest Dissertation Abstracts; PsycInfo; Social Policy and Practice including Child Data & Social Care Online; Social Science Citation Index/Web of Knowledge; ; and Trials Register of Promoting Health Interventions. We will carefully search reference lists from all studies that meet the inclusion criteria. We will hand-search journals that published included studies which we found only via reference checking and which are not indexed on databases we have searched (initially for the last 5 years and if these elicit >1 new included studies, for a further 5 years). We will search the following websites: the Campbell Library;

PROSPERO
International prospective register of systematic reviews

National Institute for Health Research

Digital Education Research Archive; OpenGrey (System for Information on Grey Literature in Europe); Database of Educational Research; International Clinical Trials Registry Platform; Schools and Students Health Education Unit Archive. We will contact subject experts to identify relevant ongoing or completed research. We will search all available clinical trials registers (e.g. clinicaltrials.gov) for relevant ongoing and unpublished trials.

Types of study to be included

In order to address RQ 1 and 3, we will include studies reporting on process evaluations. This would include studies reporting on planning, delivery, receipt or causal pathways using quantitative and/or qualitative data. These studies may report exclusively on process evaluations or report process alongside outcome data. In order to address RQ 1 and 4, we will include studies reporting on outcome evaluations, using randomized controlled trials allocating schools, classes or individuals. Controls will be students, classes or schools allocated randomly to a control group in which no or usual school health and academic education is delivered, or to a control group including another 'active' intervention. In order to address RQ2 we will draw on included process and outcome evaluations as defined above which include descriptions of intervention theories of change or logic models. In order to address RQ5, we will draw on syntheses of all of the above study types.

Condition or domain being studied

The proposed review focuses on substance use (alcohol consumption, smoking and drug use) and violence since these are important, inter-correlated outcomes which are addressed by interventions sharing common theories of change. Alcohol has been suggested to be the most harmful substance in the UK. Treating alcohol-related diseases costs the NHS in England an estimated £3.5 billion annually. The total annual societal costs of alcohol use in England are estimated at £21 billion. Alcohol related harms are strongly stratified by socioeconomic status (SES). Early initiation of alcohol use and excessive drinking are linked to later heavy drinking and alcohol-related harms and poor health. Alcohol use among young people is associated with truancy, exclusion, and poor attainment, as well as unsafe sexual behaviour, unintended pregnancies, youth offending, accidents/ injuries and violence. Preventing young people from taking up smoking is another key public health objective with 80,000 deaths due to smoking each year. In 2005-6, smoking cost the NHS £5.2 billion and wider costs amounted to £96 billion. Of smokers, 40% start in secondary school and early initiation is associated with heavier and more enduring smoking and greater mortality. Smoking among young people is a major source of health inequalities. Among UK 15-16 years olds 25% have used cannabis and 9% have used other illicit drugs. Early initiation and frequent use of 'soft' drugs may be a potential pathway to more problematic drug use in later life. Drugs such as cannabis and ecstasy are associated with increased risk of mental health problems, particularly among frequent users. Young people's drug use is also associated with accidental injury, self-harm, suicide and other 'problem' behaviours. The proposed review's other primary outcome is violence. The prevalence, harms and costs of violence among young people mean that addressing this is a public health priority. One UK study found that 10% of young people aged 11-12 reported carrying a weapon and 8% admitted attacking someone with intent to hurt them seriously. By age 15-16, 24% of students report that they have carried a weapon and 19% reported attacking someone with the intention to hurt them seriously. There are also links between aggression and anti-social behaviours in youth and violent crime in adulthood. As well as leading to further health inequalities, the economic costs to society of youth aggression, bullying and violence are high. For example, the total cost of crime attributable to conduct problems in childhood has been estimated at about £60 billion a year in England and Wales.

Participants/population

We will include studies conducted where a majority of participants are children and young people aged 4-18 years attending schools.

Intervention(s), exposure(s)

We will include school-based health curriculum interventions integrating health and academic education targeting young people age 4-18. Academic education is defined as: education in specific academic subjects; literacy; numeracy; or study skills. It does not include: social conduct in the classroom; relationships

For peer review only

with peers or staff; attitudes to education, school or teachers; or aspirations and life goals. Interventions may involve either incorporate health education into other, mainstream school subjects or aim for health education lessons to include academic education as well as teaching health knowledge and skills. Interventions may be delivered by teachers or other school staff such as teaching assistants, but may also be delivered by external providers, for example from the health, voluntary or youth service sectors. Our definition excludes interventions which: are delivered in mainstream subject lessons but do not aim to integrate health and academic education; train teachers in classroom management without student curriculum components; or are delivered exclusively outside of classrooms.

Comparator(s)/control

In order to address RQ 1 and 4, we will include studies reporting on outcome evaluations, using randomized controlled trials allocating schools, classes or individuals.
Controls will be students, classes or schools allocated randomly to a control group in which no or usual school health and academic education is delivered, or to a control group including another 'active' intervention.

Context

Schools serving students age 4-18 years.

Primary outcome(s)

We will include studies addressing one or more of the following primary review outcomes: smoking; alcohol use; legal or illegal drug use; and violence.

Timing and effect measures

We will include studies addressing one or more of the following primary review outcome measures: smoking (e.g. salivary cotinine, carbon monoxide levels, self-reported use of cigarettes); alcohol use (e.g. self-reported alcohol consumption via questionnaires or diaries); legal or illegal drug use (e.g. self-reported drug use); and violence (self-reported violence perpetration - for example, carried weapon, got into a fight - and victimisation). Informed by existing systematic reviews focused on substance use and violence among young people, outcome measures may draw on dichotomous or continuous variables, and self-report or observational data. They may use measures of frequency (monthly, weekly or daily), the number of episodes of use or an index constructed from multiple measures. Alcohol measures may examine alcohol consumption or problem drinking. Drug outcomes may examine drugs in general or specific illicit drugs, including drug convictions. Measures of violent and aggressive behaviour may examine the perpetration or victimization of physical violence including convictions for violent crime.
We will regard follow-up times of less than three months, three months to one year and more than one year post-intervention as different outcomes.

Secondary outcome(s)

Though not an inclusion criterion, we will assess academic attainment as a secondary outcome.

Timing and effect measures

Academic attainment might be measured as e.g. student standardised academic test scores, IQ tests or other validated scales; school academic performance.

Data extraction (selection and coding)

Selection of studies
Search results will be downloaded into EPPI-Reviewer 4. An inclusion criteria worksheet with guidance notes will be prepared and piloted by two reviewers screening the same 50 references. Where the two reviewers disagree, they will meet to discuss this and if possible reach a consensus. If the reviewers cannot reach consensus regarding inclusion of a specific article, judgement for selection will be referred to a third reviewer. If necessary, we will organise translation of papers published in languages in which we are not proficient. After piloting and any refinements, each reference will be screened on the basis of title and abstract for potential inclusion by one reviewer, using text-mining to prioritise screening the most relevant studies first. Full reports will be obtained for those references judged as meeting our inclusion criteria or where there is

For peer review only - http://bmjopen.bmj.com/site/about/guidelines.xhtml

insufficient information from the title and abstract to judge inclusion. A second round of screening will then occur focused on full study reports to determine which studies are included in the review. We will maintain a record of the selection process for all screened material.

Data extraction and management

Two reviewers will independently extract data from all studies meeting the inclusion criteria, using a piloted data extraction form with guidance developed for this review. Where the two authors disagree, they will meet to discuss this and if possible reach a consensus. If the reviewers cannot reach consensus regarding the particulars of data extraction for a specific study, judgement will be referred to a third reviewer.

Included studies will be described using the EPPI-Centre classification system for health promotion and public health research, supplemented by additional codes developed for this review. For all studies where relevant, we will extract information pertaining to: basic study details (individual and organizational participant characteristics, study location, timing and duration, research questions or hypotheses); study design and methods (design, allocation, blinding, sample size, control of confounding, accounting for data clustering, data collection, attrition, analysis); intervention characteristics (timing and duration, programme development, theoretical framework/logic model, content and activities, providers and details of any intervention offered to the control group); process evaluation of the intervention (feasibility, fidelity/quality, intensity, coverage/accessibility, acceptability, mechanism and context using an adapted version of an existing tool); outcome measures at follow-up(s) (reliability of measures, effect size both overall and where available by age, sex, socio-economic status and ethnic sub-group). The two reviewers will independently enter data from the data extraction forms into EPPI-Reviewer 4. If included studies are reported in languages that cannot be translated by the review team, a review author will complete the data extraction form in conjunction with a translator.

Published reports may be incomplete in a wide range of ways. For example: they may not report sufficient detail about their participants for our equity analysis; they may not present information on all the outcomes that were measured (possibly resulting in outcome reporting bias); they may not provide sufficient information about the intervention for accurate characterisation; and they may not report the necessary statistical information for the calculation of effect sizes. In all cases where there is a danger of missing data affecting our analysis, we will contact authors of papers wherever possible to request additional information. If authors are not traceable or sought information is unavailable from the authors within two months of contacting them, we will record that the study information is missing on the data extraction form, and this will be captured in our risk of bias assessment of the study.

Risk of bias (quality) assessment

We will assess the quality of theories of change using a modified version of the criteria developed in our ongoing NIHR-funded systematic review of positive youth development interventions, which for example assess the clarity with which constructs are defined and inter-related. We will assess the quality of the qualitative and quantitative elements of process evaluations using standard Critical Appraisal Skills Program and EPPI-Centre tools. These address the rigour of: sampling; data collection; data analysis; the extent to which the study findings are grounded in the data; whether the study privileges the perspectives of participants; the breadth of findings; and depth of findings. These are then used to assign studies to two categories of 'weight of evidence'. First, reviewers will assign a weight (low, medium or high) to rate the reliability or trustworthiness of the findings (the extent to which the methods employed were rigorous/could minimise bias and error in the findings). Second, reviewers will assign an additional weight (low, medium, high) to rate the usefulness of the findings for shedding light on factors relating to the research questions. Guidance will be given to reviewers to help them reach an assessment on each criterion and the final weight of evidence. The two reviewers will then meet to compare their assessments, resolving any differences through discussion and, where necessary, by calling on a third reviewer. For outcome evaluations, we will assess risk of bias within each included study using the tool outlined in the Cochrane Handbook for Systematic Reviews of Interventions. For each study, two reviewers will independently judge the likelihood of bias in seven domains: sequence generation; allocation concealment; blinding (of participants, personnel, or outcome assessors); incomplete outcome data; selective outcome reporting; and other sources of bias (e.g. recruitment bias in cluster-randomised studies); and intensity/type of comparator. Each study will subsequently be identified as 'high risk', 'low risk' or 'unclear risk' within each domain. In cases of

For peer review only - http://bmjopen.bmj.com/site/about/guidelines.xhtml

disagreement, the reviewers will meet to seek consensus but where they cannot, we will refer judgement to a third reviewer. We will assess reporting bias according to Sterne's guidance. We will reduce the effect of reporting bias by focusing synthesis on studies rather than publications, avoiding duplicated data. Following the Cho statement on redundant publications, we will attempt to detect duplicate studies and, if multiple articles report on the same study, we will extract data only once. We will prevent location bias by searching across multiple databases. We will prevent language bias by not excluding any article based on language.

Strategy for data synthesis

RQ1 and 2: Thematic synthesis of intervention descriptions and process data:
Using thematic synthesis methods we will undertake a number of syntheses. Intervention descriptions (RQ1) and theories of change (RQ2) will first be analysed to develop a taxonomy of interventions integrating health and academic education. Syntheses of theories of change (RQ2) and process evaluations (RQ3) will be used to understand potential mechanisms of action. Syntheses of process evaluations (RQ3) will be used to understand: characteristics of interventions, participants and context acting as potential barriers and facilitators of implementation and receipt (RQ2); and an assessment of potential applicability to the UK. These syntheses will not be restricted to studies judged to be of high quality. Instead conclusions drawing on poorer quality evidence will be given less interpretive weight. First, the reviewers will prepare detailed evidence tables to describe: the methodological quality of each study; details of the intervention examined; study site/population; and full findings. Second, the two reviewers will undertake pilot analysis of two studies. The reviewers will read and re-read data contained within the evidence tables relating to the two high-quality studies, applying line-by-line codes to capture the content of the data. They will draft memos explaining these codes. Coding will begin with in-vivo codes which closely reflect the words used in findings sections. The reviewers will then group and organise codes, applying axial codes reflecting higher-order themes. The two reviewers will meet to compare and contrast their coding of these first two high-quality studies, developing an overall set of codes. Third, the two reviewers will go on to code the remaining studies drawing in the agreed set of codes but developing new in-vivo and axial codes as these arise from the analytical process, and again writing memos to explain these codes. At the end of this process, the two reviewers will meet to compare their sets of codes and memos. They will identify commonalities, differences of emphasis and contradictions with the aim of developing an overall analysis which draws on the strengths of the two sets of codes and which resolves any contradictions or inconsistencies, drawing on a third reviewer if necessary to achieve this. Through this process will be developed an explanatory framework to understand factors affecting implementation. Results will be presented to PPI stakeholders who will determine which interventions they think are applicable to the UK.
RQ4: Synthesis of outcome data:
We will first produce a narrative account of the effectiveness of these types of interventions. This narrative synthesis will be ordered by outcome then within this by age group, intervention type and follow-up time. Outcomes will be categorised into violence, smoking tobacco, drinking alcohol, using other drugs and academic attainment. Age will be categorised by the key-stage age-ranges used in the English educational system. Categorisation by intervention type will be informed by our prior thematic synthesis of intervention descriptions and theories of change through which we will have produced a taxonomy of interventions. This taxonomy may refer to: whether interventions incorporate health education into other, mainstream school subjects or aim for health education lessons to include teaching of academic as well as health knowledge and skills; lesson frequency; style of delivery; or other aspects of interventions which appear to be critical from our preliminary synthesis. We will describe study results in the 'characteristics of included studies' table, or enter the data into additional tables. We will then produce forest plots for each of our review outcomes, with separate plots for different outcomes and follow-up times, age groups and intervention types. Plots will include point estimates and standard errors for each study, such as risk ratios for dichotomous outcomes or standardised mean differences for continuous outcomes. Once we know the number of studies and the extent of heterogeneity among the studies (as determined both by a Cochran's Q test and inspection of the I2), we will make a decision whether to calculate pooled effect sizes. The results of statistical tests will be evaluated in accordance with the Cochrane handbook. If an indication of substantial heterogeneity is determined (e.g. study-level I^2 value greater than 50%) that cannot be explained through meta-regressions, then we will not produce a pooled estimate and will present only the narrative summary.

For peer review only

PROSPERO
International prospective register of systematic reviews

National Institute for Health Research

When studies are found to be statistically heterogeneous, we will use a random-effects model; otherwise we will use a fixed-effects model. When using the random-effects model, we will conduct a sensitivity check by using the fixed-effect model to reveal differences in results. If we do produce pooled estimates, we will consider using a multilevel meta-analysis model to synthesise effect sizes. This is because outcome evaluations are likely to include multiple measures of conceptually related outcomes and multi-level meta-analysis improves on previous strategies for dealing with multiple relevant effect sizes per study, such as meta-analysing within studies or choosing one effect size, by including all relevant effect sizes but adjusting for inter-dependencies within studies. Unlike multivariate meta-analysis, it does not require the variance-covariance matrix of included effect sizes to be known. We will estimate separate models for substance use, violence and educational attainment outcomes. We will estimate separate models for substance use, violence and educational attainment outcomes, and for different age-ranges. We will examine substance use outcomes together in one analysis, as well as separated into smoking, alcohol, illicit drug use and any ‘omnibus’ measures of substance use. We will regard follow-up times of less than three months, three months to one year and more than one year post-intervention as different outcomes. We will run these models for interventions overall and where sufficient studies are found we will run separate models for different intervention categories and comparators. This categorisation will be informed by the taxonomy derived from our prior synthesis of intervention descriptions and theories of change. Where meta-analyses are performed, we will include pooled effect sizes in forest plots, with the individual study point estimates weighted by a function of their precision.
Prior to synthesis, we will check for correct analysis (where appropriate) by cluster and report values of: intra-cluster correlation coefficients (ICC), cluster size, data for all participants or effect estimates and standard errors. Where proper account has not been taken of data clustering, we will correct for this by inflating the standard error by the square root of the design effect. Where ICCs are not reported, we will contact authors to request this information or impute one, based on values reported in other studies. Where imputation is necessary, we will undertake sensitivity analyses to assess the impact of a range of possible values. In other instances of missing data (such as missing population information), it may not be possible to include a study in a particular analysis if, for example, it is impossible to classify the population using our equity tool. We will use the GRADE approach as described in the Cochrane Handbook for Systematic Reviews of Interventions to present the quality of evidence and ‘Summary of findings’ tables. The downgrading of the quality of a body of evidence for a specific outcome will be based on five factors: limitations of study; indirectness of evidence; inconsistency of results; precision of results; and publication bias. The GRADE approach specifies four levels of quality (high, moderate, low and very low). If sufficient studies are found, we will draw funnel plots to assess the presence of possible publication bias (trial effect versus standard error). While funnel plot asymmetry may indicate publication bias, this can be misleading with a small number of studies. We will discuss possible explanations for any asymmetry in the review in light of our number of included studies. We will undertake a sensitivity analysis to explore whether the findings of the review are robust in light of the decisions made during the review process. We will also assess the impact of risk of bias in the included studies via restricting analyses to studies deemed to be at low risk of selection bias, performance bias and attrition bias. Where data allow, we will undertake additional exploratory meta-analyses to determine intervention effects on theorised intermediate outcomes (such as knowledge, skills, social norms) to examine the plausibility that these might mediate or otherwise precede behavioural effects. Such analyses will be informed by the synthesis of theories of change and process evaluation findings to avoid data-dredging.

Analysis of subgroups or subsets

If we consider that we have unexplained statistical heterogeneity in any of our study groupings, we will investigate this further using subgroup and sensitivity analyses. We will analyse the effectiveness of the subset of interventions identified by stakeholders as relevant to the UK context. Where possible we will examine intervention effects by participant sub-groups (for example in terms of age, socioeconomic status, sex and ethnicity) and contexts (for example in terms of school-level deprivation) in order to examine potential impacts on health inequalities. This will draw on existing methods involving an ‘equity lens’ employing meta-analyses of subgroup effects from included studies and/or meta-regression drawing on studies with different participant or site characteristics to assess whether these moderate effects.
RQ5: Meta-regression and qualitative comparative analysis:

For peer review only - http://bmjopen.bmj.com/site/about/guidelines.xhtml

PROSPERO
International prospective register of systematic reviews

NHS
National Institute for Health Research

If at least ten studies are found, we will employ meta-regression using Stata to investigate what factors moderate intervention effects in order to examine what characteristics of intervention, deliverers, contexts and students moderate effectiveness (RQ5). It may not be feasible to apply this method if we judge there are too many confounders or insufficient data, or if meta-regression is unable to account for interdependencies in complex interventions. Hence, we will complement meta-regression with qualitative comparative analysis, adapted for use in research synthesis to assess necessary and sufficient conditions for intervention effectiveness. As with our current review of positive youth development, the use of initial hypotheses derived from work addressing RQ 2 and 3 will protect us from 'dredging' the data for spurious statistically significant results. The required steps of 'qualitatively anchoring' outcomes in qualitative comparative analysis will ensure that changes in outcomes are meaningful and not simply statistical artefacts with little relevance for decision-making. We should stress that meta-regression and qualitative comparative analysis will be exploratory, hypothesis-building analyses since these will draw on observational rather than experimental comparisons.

Contact details for further information

Chris Bonell
c.bonell@ioe.ac.uk

Organisational affiliation of the review

University College London Institute of Education
http://www.ioe.ac.uk/index.html

Review team members and their organisational affiliations

Professor Chris Bonell. UCL Institute of Education
Professor James Thomas. UCL Institute of Education
Dr Adam Fletcher. Cardiff University
Professor Rona Campbell. University of Bristol
Dr GJ Melendez-Torres. University of Warwick
Ms Claire Stansfield. UCL Institute of Education
Dr Tara Tancred. UCL Institute of Education

Collaborators

Dr Rob Anderson. University of Exeter

Anticipated or actual start date

01 January 2016

Anticipated completion date

31 August 2017

Funding sources/sponsors

National Institute for Health Research Public Health Research Programme Grant Number 14/52/15

Conflicts of interest

None known

Language

English

Country

England, Wales

Published protocol

http://www.crd.york.ac.uk/PROSPEROFILES/26464_PROTOCOL_20160011.pdf

For peer review only

PROSPERO
International prospective register of systematic reviews

National Institute for Health Research

Stage of review
Review_Ongoing

Subject index terms status
Subject indexing assigned by CRD

Subject index terms
Drug Users; Health Education; Humans; Prescription Drug Misuse; Schools; Socioeconomic Factors; Substance-Related Disorders; Violence

Date of registration in PROSPERO
22 September 2015

Date of publication of this version
11 January 2016

Revision note for this version
Modifications made 11/1/16Searches amended slightly - reflecting advice of information scientist Claire Stansfield.Inclusion criteria modified slightly - interventions integrating health and academic biology education no longer excluded

Details of any existing review of the same topic by the same authors

Stage of review at time of this submission
The review has not started

Stage	Started	Completed
Preliminary searches	No	No
Piloting of the study selection process	No	No
Formal screening of search results against eligibility criteria	No	No
Data extraction	No	No
Risk of bias (quality) assessment	No	No
Data analysis	No	No

Revision note
Modifications made 11/1/16Searches amended slightly - reflecting advice of information scientist Claire Stansfield.Inclusion criteria modified slightly - interventions integrating health and academic biology education no longer excluded

Versions
22 September 2015
20 November 2015
11 January 2016

PROSPERO
This information has been provided by the named contact for this review. CRD has accepted this information in good faith and registered the review in PROSPERO. CRD bears no responsibility or liability for the content of this registration record, any associated files or external websites.

For peer review only

PROSPERO
International prospective register of systematic reviews

National Institute for Health Research

For peer review only

Additional search and synthesis methods

We searched 19 electronic databases. The original list of databases was amended after consultation with our information scientist, as informed by initial searches.

- ASSIA via Proquest
- Australian Educational Index via Proquest
- BiblioMap (Database of health promotion research) via EPPI-Centre
- British Educational Index via EBSCOhost
- Cochrane Central Register of Controlled Trials via the Cochrane Library
- Cochrane Database of Systematic Reviews via the Cochrane Library
- Database of Abstracts of Reviews of Effects via the Cochrane Library
- Database of Promoting Health Effectiveness Reviews (DoPHER) via EPPI-Centre
- Dissertation Abstracts (UK theses, all dates; global theses 2010-2015) via Proquest
- Econlit via EBSCO
- Educational Research Index Citations via EBSCO
- Health Technology Assessment Database via the Cochrane Library
- International Bibliography of the Social Sciences via Proquest
- MEDLINE via OVID
- NHS Economic Evaluation Database
- PsycINFO via OVIDa
- Social Policy and Practice Including Child Data & Social Care Online via OVID
- Social Science Citation Index via Web of Knowledge
- Trials Register of Promoting Health Interventions via EPPI-Centre

We also searched the following 32 websites:

- Cambridge Journals
- Centers for Disease Control and Prevention: Smoking & Tobacco Use
- Child and Adolescent Research Unit
- Childhoods Today
- Children in Scotland
- Children in Wales
- Community Research and Development Information Service
- Database of Educational Research (EPPI-Centre)
- Drug and Alcohol Findings Effectiveness Bank
- Google
- Google Scholar
- Government of Wales
- Government of Scotland
- Joseph Rowntree Foundation
- National Criminal Justice Reference Service
- National Society of the Prevention of Cruelty to Children
- National Youth Agency
- Northern Ireland Executive
- OpenGrey
- Personal Social Services Research Unit
- Project Cork
- UCL-IOE Digital Education Resource Archive
- UK Clinical Research Net Study Portfolio
- University of Illinois at Urbana Champaign
- US Centre for Substance Abuse Prevention
- Social Issues Research Centre
- The Campbell Library
- The Children's Society
- The Open Library
- The Schools and Students' Health Education Unit Archive
- WHO International Clinical Trials Registry Platform
- Young Minds: Child & Adolescent Mental Health

For peer review only

PsycINFO search string

1. ((substance? or drug? or drinking or alcohol* or solvent?) adj1 ("use" or abus* or misuse*)).ti,ab.
2. ((substance? or drug? or drinking or alcohol* or solvent?) adj1 (usage or intake or using or taking or behavio* or user?)).ti,ab.
3. (drinking adj1 (alcohol* or behavio*)).ti,ab.
4. Alcohol.ti,ab.
5. (smoke or smoking or tobacco or cigarette? or smoker? or cannabis or marijuana).ti,ab.
6. (aggression or aggressive or bully* or delinquen* or "conduct problem*" or "conduct disorder?" or "antisocial" or "anti social" or violence or violent or (volatile adj behavio*) or victimi* or hostile or hostility or perpetrat*).ti,ab.
7. (Externalising or externalizing).ti,ab.
8. emotion*.ti,ab.
9. PSHE.ti,ab.
10. ("Health literacy" or "health education" or "health promotion" or "preventive health" or "primary prevention" or "health information" or "promoting health" or "health promoting" or "health promotion" or "health maintenance").ti,ab.
11. "Public health".ti,ab.
12. ("wellbeing" or "well being").ti,ab.
13. "mental health".ti,ab.
14. 1 or 2 or 3 or 4 or 5 or 6 or 7 or 8 or 9 or 10 or 11 or 12 or 13
15. ((curric* or lesson? or classes or classroom? or subject? or intervention? or program* or education or initiative? or learn or learning or teach or teaching or outcome* or attainment or achievement or assessment or effect* or impact* or score? or scoring* or skill? or knowledge or competen* or performance) adj3 (Academic or academically or Scholastic or scholar* or Mainstream or "main stream")).ti,ab.
16. ((curric* or lesson? or classes or classroom? or subject? or learn or learning or teach or teaching or attainment or achievement or assessment or score? or scoring* or skill? or knowledge or competen* or performance) adj3 School?).ti,ab.
17. ((intervention? or program* or initiative? or effect* or impact* or education) adj1 School?).ti,ab.
18. (class adj1 (Academic or academically or Scholastic or scholar* or School? or Mainstream or "main stream")).ti,ab.
19. ((curric* or lesson? or classes or classroom? or subject? or education or learn or learning or teach or teaching or attainment or achievement or score? or scoring* or skill? or knowledge or competen*) adj3 (study or core or generic)).ti,ab.
20. (class adj1 (study or core or generic)).ti,ab.
21. ((curric* or lesson? or classes or classroom? or subject? or attainment or achievement or assessment or score? or scoring* or competenc* or performance) adj3 ((Education not ("patient education" or "continuing education")) or educational)).ti,ab.
22. (class adj1 ((Education not ("patient education" or "continuing education")) or educational)).ti,ab.
23. (outcome* adj1 (education or educational)).ti,ab.
24. ((curric* or lesson? or classroom? or classes or subject? or intervention? or program* or initiative? or education or teach* or outcome* or attainment or achievement or assessment or effect* or impact* or score? or scoring* or skill? or knowledge or competen* or performance) adj3 (learn or learning)).ti,ab.
25. (class adj1 (learn or learning)).ti,ab.
26. ((curric* or lesson? or classes or classroom or class or subject? or education or teach* or learning or teach or teaching or learn or attainment or achievement or assessment or score? or scoring* or skill? or knowledge or competen* or performance) adj3 (art or arts or math* or science? or humanities or chemistry or physics or language* or geography or (history not ("medical history" or "health history" or "familial history" or "family history")) or numeracy or (literacy not "health literacy") or grammar or grammer or reading or writing)).ti,ab.
27. (((curric* or lesson? or classroom or classes or subject? or skill?) adj3 literature) or "literature class").ti,ab.
28. ("Education reform" or "Instructional support" or "School reform" or "Classroom organi*" or (Commit* adj3 (school or education or learning)) or (Engag* adj3 (school or education or learning)) or "Character development" or "Whole school" or "School level" or "School wide" or schoolwide).ti,ab.
29. ((Comprehensive adj3 school) and (intervention? or program* or initiative? or outcome* or effect* or impact*)).ti,ab.
30. ((Integrat* or Combin* or Infuse or infused or infusion or sustainable) adj3 (curric* or lesson? or classes or classroom or syllabus or subject? or education or learn or learning or teach or teaching)).ti,ab.
31. (((Integrat* or Combin* or Infuse or infused or infusion or sustainable) adj3 (intervention* or program* or initiative*)) and school?).ti,ab.
32. ((school or education or core or generic or teaching or learning) adj3 syllabus).ti,ab.

For peer review only

33. 15 or 16 or 17 or 18 or 19 or 20 or 21 or 22 or 23 or 24 or 25 or 26 or 27 or 28 or 29 or 30 or 32
34. (child* or schoolchild* or youth* or "young people*" or "young person" or teen* or adolescen* or juvenile* or preadolescen* or boy? or girl?).ti,ab.
35. (curric* or lesson? or classes or classroom? or subject? or school? or syllabus or "junior high" or "senior high" or "junior education" or "elementary education" or "primary education").ti,ab.
36. 34 and 35
37. ("secondary school?" or "primary school?" or "comprehensive school?" or "school education" or "high school?" or "grammar school?" or "private school?" or "public school?" or "mainstream school*" or "compulsory education" or "statutory education" or "middle school?" or "junior school?" or "senior school?" or "primary education" or "secondary education" or "elementary school?" or "elementary education" or "mainstream education" or "compulsory school*" or "statutory school*" or "sixth form college?" or "post-16 education" or "junior high" or "senior high" or "reception class" or "post primary").ti,ab.
38. ((school? or junior? or elementary or senior? or primary or "sixth form" or grade) adj10 student?).ti,ab.
39. pupil?.ti,ab.
40. 36 or 37 or 38
41. (University or universities or freshmen or sophomore? or "higher education" or "tertiary education" or ((registrar* or workplace? or clinical or medical or nursing or nurse? or doctor? or continuing or adult? or patient?) adj1 (education or educating or profession* or student?)) or "professional education").ti.
42. 40 not 41
43. 14 and 33 and 42
44. "Elementary School Students"/ or "Intermediate School Students"/ or "Primary School Students"/ or "Middle School Students"/ or "High School Students"/ or "Junior High School Students"/ or "Kindergarten Students"/ or "High School Education"/ or "Middle School Education"/ or "Secondary Education"/ or "Junior High Schools"/ or "High Schools"/ or "Schools"/ or "Elementary Schools"/ or "Middle Schools"/
45. "Drug Abuse Prevention"/ or "Health Education"/ or "Drug Education"/ or "Health Promotion"/ or "Public Health"/ or "Health Promotion"/ or "Preventive Medicine"/ or Health behaviour/ or Harm reduction/ or Health literacy/ or exp Health screening/ or Primary Mental health prevention/ or Prevention/ or Public health/ or Lifestyle changes/ or Lifestyle/ or Health literacy/
46. "Tobacco Smoking"/ or "Smoking Cessation"/ or "Marijuana Usage"/ or "Drinking Behavior"/ or "Social Drinking"/ or "Binge Drinking"/ or "Underage Drinking"/ or "Alcohol Abuse"/ or "Alcohol Drinking Patterns"/ or "Alcohol Intoxication"/ or "Alcoholism"/ or "Heroin Addiction"/ or "Drug Addiction"/ or "Drug Dependency"/ or "Drug Usage"/ or "Inhalant Abuse"/ or "Drug Abuse"/ or "Glue Sniffing"/ or "Predelinquent Youth"/ or "Cyberbullying"/ or "School Violence"/ or "Teasing"/ or "Juvenile Delinquency"/ or "Physical Abuse"/ or "Verbal Abuse"/ or "Violence"/ or "Harassment"/ or "Antisocial Behavior"/ or "Bullying"/ or "Perpetrators"/ or "Threat"/ or "Victimization"/ or "Relational Aggression"/ or "Aggressive Behavior"/ or "Behavior Problems"/ or "Behavior Disorders"/ or "Conduct Disorder"/ or "Drug Education"/ or "Drug Abuse Prevention"/ or "Harm Reduction"/
47. emotions/ or emotional development/
48. emotional adjustment/ or emotional disturbances/ or emotional control/
49. mental health/ or primary mental health prevention/ or well being/
50. "Curriculum"/ or "Curriculum Based Assessment"/ or "Curriculum Development"/ or "School Learning"/ or "Classroom Environment"/ or "Academic Environment"/ or "Teacher Effectiveness"/ or "Teacher Effectiveness Evaluation"/ or "Educational Program Evaluation"/ or "Course Evaluation"/ or "learning environment"/
51. 14 or 45 or 46 or 47 or 48 or 49
52. 33 or 50
53. 42 or 44
54. 51 and 52 and 53

For peer review only

PRISMA 2009 Checklist

Section/topic	#	Checklist item	Reported on page #
**TITLE**			
Title	1	Identify the report as a systematic review, meta-analysis, or both.	1
**ABSTRACT**			
Structured summary	2	Provide a structured summary including, as applicable: background; objectives; data sources; study eligibility criteria, participants, and interventions; study appraisal and synthesis methods; results; limitations; conclusions and implications of key findings; systematic review registration number.	2
**INTRODUCTION**			
Rationale	3	Describe the rationale for the review in the context of what is already known.	3-4
Objectives	4	Provide an explicit statement of questions being addressed with reference to participants, interventions, comparisons, outcomes, and study design (PICOS).	4
**METHODS**			
Protocol and registration	5	Indicate if a review protocol exists, if and where it can be accessed (e.g., Web address), and, if available, provide registration information including registration number.	6
Eligibility criteria	6	Specify study characteristics (e.g., PICOS, length of follow-up) and report characteristics (e.g., years considered, language, publication status) used as criteria for eligibility, giving rationale.	6
Information sources	7	Describe all information sources (e.g., databases with dates of coverage, contact with study authors to identify additional studies) in the search and date last searched.	Online File 1
Search	8	Present full electronic search strategy for at least one database, including any limits used, such that it could be repeated.	Online File 1
Study selection	9	State the process for selecting studies (i.e., screening, eligibility, included in systematic review, and, if applicable, included in the meta-analysis).	7
Data collection process	10	Describe method of data extraction from reports (e.g., piloted forms, independently, in duplicate) and any processes for obtaining and confirming data from investigators.	7
Data items	11	List and define all variables for which data were sought (e.g., PICOS, funding sources) and any assumptions and simplifications made.	Online File 1
Risk of bias in individual studies	12	Describe methods used for assessing risk of bias of individual studies (including specification of whether this was done at the study or outcome level), and how this information is to be used in any data synthesis.	7
Summary measures	13	State the principal summary measures (e.g., risk ratio, difference in means).	N/A
Synthesis of results	14	Describe the methods of handling data and combining results of studies, if done, including measures of consistency (e.g., I^2) for each meta-analysis.	8

For peer review only

For peer review only - http://bmjopen.bmj.com/site/about/guidelines.xhtml

PRISMA 2009 Checklist

Section/topic	#	Checklist item	Reported on page #
Risk of bias across studies	15	Specify any assessment of risk of bias that may affect the cumulative evidence (e.g., publication bias, selective reporting within studies).	8
Additional analyses	16	Describe methods of additional analyses (e.g., sensitivity or subgroup analyses, meta-regression), if done, indicating which were pre-specified.	8
**RESULTS**			
Study selection	17	Give numbers of studies screened, assessed for eligibility, and included in the review, with reasons for exclusions at each stage, ideally with a flow diagram.	9, Figure 1
Study characteristics	18	For each study, present characteristics for which data were extracted (e.g., study size, PICOS, follow-up period) and provide the citations.	8-10, Table 1-3
Risk of bias within studies	19	Present data on risk of bias of each study and, if available, any outcome level assessment (see item 12).	Table 2
Results of individual studies	20	For all outcomes considered (benefits or harms), present, for each study: (a) simple summary data for each intervention group (b) effect estimates and confidence intervals, ideally with a forest plot.	11-16
Synthesis of results	21	Present results of each meta-analysis done, including confidence intervals and measures of consistency.	N/A
Risk of bias across studies	22	Present results of any assessment of risk of bias across studies (see Item 15).	N/A
Additional analysis	23	Give results of additional analyses, if done (e.g., sensitivity or subgroup analyses, meta-regression [see Item 16]).	N/A
**DISCUSSION**			
Summary of evidence	24	Summarize the main findings including the strength of evidence for each main outcome; consider their relevance to key groups (e.g., healthcare providers, users, and policy makers).	16
Limitations	25	Discuss limitations at study and outcome level (e.g., risk of bias), and at review-level (e.g., incomplete retrieval of identified research, reporting bias).	17
Conclusions	26	Provide a general interpretation of the results in the context of other evidence, and implications for future research.	17-18
**FUNDING**			
Funding	27	Describe sources of funding for the systematic review and other support (e.g., supply of data); role of funders for the systematic review.	18

From: Moher D, Liberati A, Tetzlaff J, Altman DG, The PRISMA Group (2009). Preferred Reporting Items for Systematic Reviews and Meta-Analyses: The PRISMA Statement. PLoS Med 6(7): e1000097. doi:10.1371/journal.pmed1000097

For more information, visit: **www.prisma-statement.org**.

For peer review only - http://bmjopen.bmj.com/site/about/guidelines.xhtml
